# Supramolecular Polymer Brushes Grafted via Atom Transfer Radical Polymerization from Surfaces Presenting Non-covalent, Host–Guest Complex-Based Initiators

**DOI:** 10.1021/acs.macromol.5c00058

**Published:** 2025-03-19

**Authors:** Friederike K. Metze, Harm-Anton Klok

**Affiliations:** † Institut des Matériaux and Institut des Sciences et Ingénierie Chimiques, Laboratoire des Polymères, Bâtiment MXD, Station 12, École Polytechnique Fédérale de Lausanne (EPFL), CH-1015 Lausanne, Switzerland; ‡ National Center of Competence in Research Bio-inspired Materials, Chemin des Verdiers 4, CH-1700 Fribourg, Switzerland

## Abstract

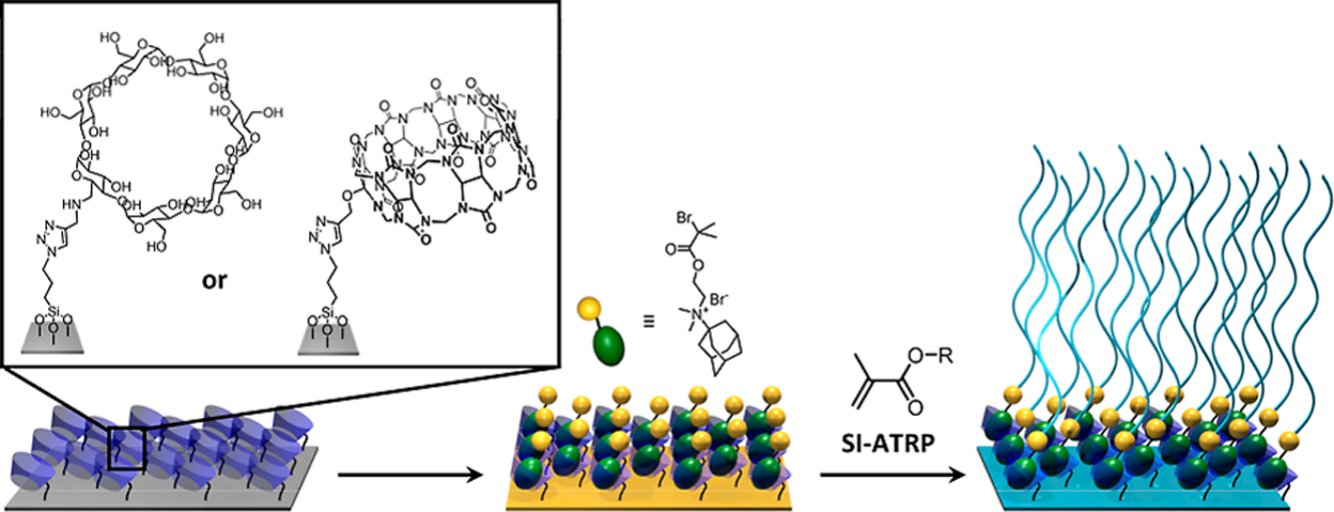

This paper describes the synthesis of supramolecular polymer brushes via surface-initiated polymerization from adamantane-functionalized initiators that are noncovalently bound to β-cyclodextrin- or cucurbit[7]uril-modified substrates. Surface-initiated atom transfer radical polymerization in aqueous media allowed the growth of various hydrophilic polymer brushes with film thicknesses of up to 40 nm from β-cyclodextrin functionalized surfaces. The adamantane moiety not only forms a host–guest complex with β-cyclodextrin, but also with cucurbit[7]uril, which provides opportunities to study the effect of the binding strength of these supramolecular motifs on the film thickness and grafting density of the resulting polymer brushes. Comparison of supramolecular polymer brushes grown from β-cyclodextrin and cucurbit[7]uril-based noncovalent initiators reveals differences in grafting density that are much smaller than expected based on the differences in the solution binding constant of the corresponding host–guest complexes. Both the β-cyclodextrin as well as the cucurbit[7]uril-anchored supramolecular brushes were remarkably robust toward detachment of the polymer grafts. These observations are attributed to the fact that the rates of formation and dissociation of the host–guest complexes are much faster as compared to diffusion of free, detached polymer chains through the polymer brush film. As a result, the surface-grafted polymer brush presents a steric barrier that prevents detachment of individual chains, and also allows surface-initiated polymerization from substrates to which initiators are bound via putatively weak β-cyclodextrin-based host–guest complexes.

## Introduction

Tethering polymers via one of their chain ends to a surface results in the formation of thin films that are referred to as polymer brushes.^1−11^ While polymer brushes (most) often are obtained by covalent anchoring of polymer chains to the surface, noncovalent interactions can also be used. Noncovalently anchored, supramolecular polymer brushes can be prepared via a number of strategies.^12^ One well-established approach for the preparation of supramolecular polymer brushes is the physisorption of block copolymers.^13−21^ Supramolecular polymer brushes have also been prepared by harnessing π–π,^22−29^ electrostatic and hydrogen bonding interactions^30−33^ to either graft appropriately chain end functional polymers to the surface of interest, or to immobilize initiators that enable grafting of polymers from the surface via e.g. controlled radical polymerization methods.

Another strategy toward supramolecular polymer brushes involves the use of well-defined host–guest complexes to chain-end anchor polymer chains to a surface either via grafting-onto or grafting-from approaches. There is a wide variety of molecular receptors that are able to selectively form stable inclusion complexes, which potentially can be used to prepare supramolecular polymer brushes. The binding constants of the host–guest complexes that are available span several orders of magnitude, and for some receptors depend on, and can be tuned by varying the nature of the guest.^12,34−36^ This is attractive as it provides potential opportunities to engineer the reversibility and responsiveness of supramolecular polymer brushes.

Two classes of molecular receptors that are attractive for the preparation of supramolecular polymer brushes are the cucurbit[*n*]urils (CB[*n*]) and the cyclodextrins (CD). Cucurbit[*n*]urils are macrocyclic hosts composed of glycoluril units that are able to form very stable inclusion complexes in aqueous solution with a range of guest molecules. The CB[7] homologue, for example, forms inclusion complexes with adamantane (Ada)-based guests, which are characterized by exceptionally high binding constants (log *K*_A_ ∼ 10^12^).^37−39^ Recently, the preparation of polymethacrylate brushes via surface-initiated atom transfer radical polymerization from surface-attached 2-bromoisobutyrate-functionalized CB[7]-adamantane host–guest complexes was reported.^40^ The CB[8] cucurbit[*n*]uril homologue has been used to produce polymer brushes via the “grafting to” approach.^41^ Cyclodextrins, and in particular the 7-membered β-cyclodextrin (β-CD) homologue, are an interesting complement to the CB[*n*] receptors. β-CD can form complexes with some of the same guest molecules as CB[7]. The binding constants of adamantane—β-CD host–guest complexes, however, are significantly smaller (typically log *K*_A_ ∼3–4 vs ∼12) as compared to those of the corresponding CB[*n*] host–guest complexes.^42−44^ While CD-based host–guest complexes have been successfully used to produce polymer brushes via the “grafting to” strategy,^45−48^ the use of this receptor motif to graft supramolecular polymer brushes via surface-initiated polymerization has not yet been explored. As both molecular receptors are able to form inclusion complexes with adamantane guests (yet with different binding constants), the use of β-CD and CB[7]-based noncovalent initiators provides an attractive model system to study the effect of the host–guest binding constant on the growth of polymer brushes via surface-initiated polymerization. The binding constant of the host–guest complex may impact the surface concentration of initiators for surface-initiated polymerization (and as a consequence grafting density and polymer brush film thickness), the robustness of the noncovalently tethered polymer grafts, as well as the decomplexation of the host–guest complexes and the putative degrafting of the supramolecular polymer brushes. Although several reports have described the use of well-defined host–guest complexes to prepare supramolecular polymer brushes, only little effort has been made to study the effect of the binding strength of these supramolecular motifs on the film thickness and grafting density of the resulting polymer brushes.

**Scheme 1:**
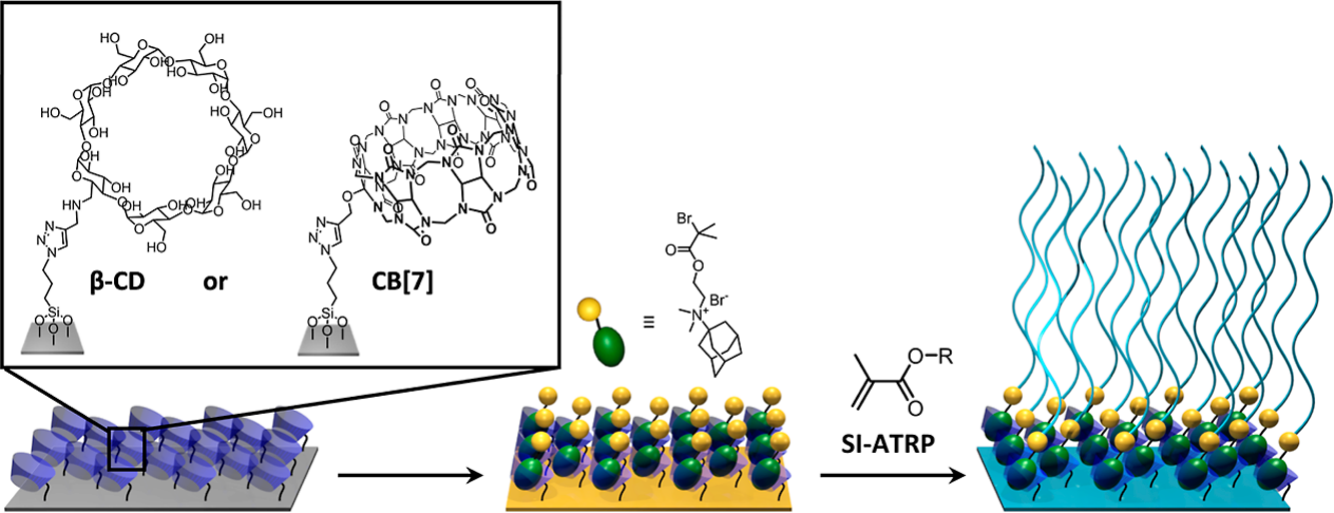
Grafting of Supramolecular Polymer Brushes From Surface-Anchored Adamantane@β-CD or Adamantane@CB[7] Host–Guest Complexes that Present Initiators for Atom Transfer Radical Polymerization (ATRP)

This paper first investigates the use of the Ada@β-CD host–guest complex to produce surface-attached initiators for the surface-initiated atom transfer radical polymerization (ATRP) of a variety of water-soluble methacrylate monomers (Scheme 1). In the second part of this study, the effect of the binding constant of the host–guest complex on the growth of supramolecular brushes will be investigated by comparing the surface-initiated atom transfer radical polymerization (SI-ATRP) of four different methacrylates from surfaces that present CB[7] and β-CD bound ATRP initiators. The results of these experiments provide first insight into the effect of the binding constant of the host–guest complex on the grafting density and stability of supramolecular brushes grown from these surfaces.

## Results and Discussion

**Scheme 2:**
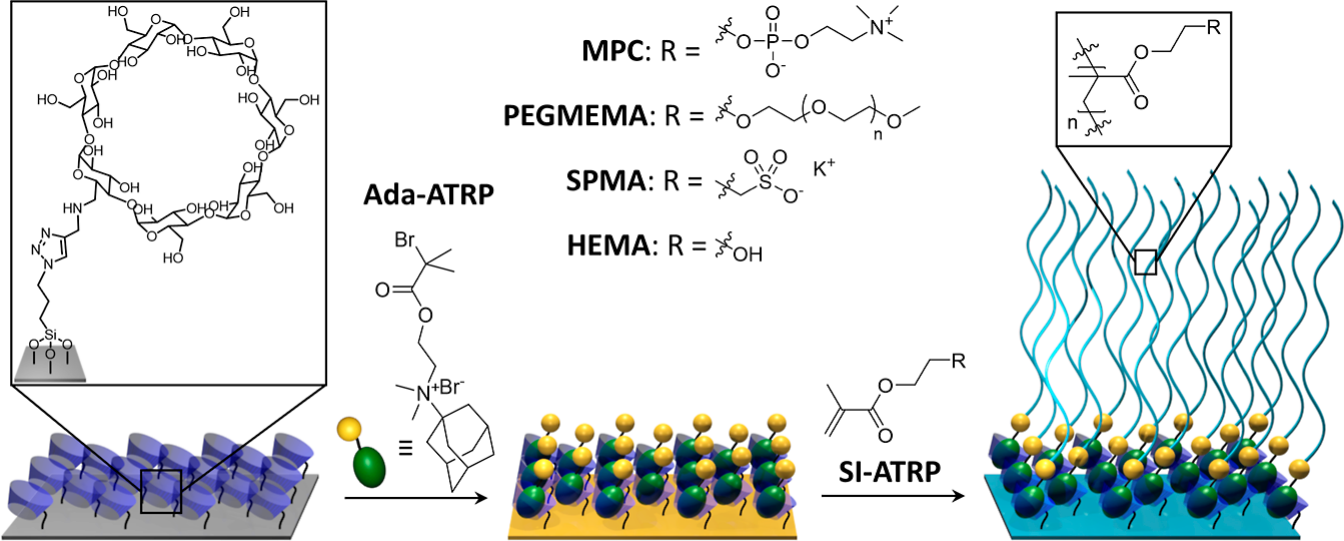
Synthesis of Supramolecular Polymethacrylate Brushes via Grafting From β-CD-Immobilized Non-covalent Initiators

The synthesis of supramolecular polymer brushes via surface-initiated atom transfer radical polymerization (ATRP) using β-CD-immobilized noncovalent initiators is illustrated in Scheme 2. Polymer brush growth starts with the preparation of a β-CD modified substrate. This is followed by the formation of a surface-bound host–guest complex between the β-CD moieties and an ATRP-initiator modified quaternary ammonium adamantane derivative (Ada-ATRP), and subsequent growth of supramolecular polymer brushes via SI-ATRP. To allow for noncovalent immobilization of the ATRP initiator, the 2-bromoisobutyrate moiety was modified with a quaternary ammonium adamantane (Ada) group. Both β-CD and CB[7] are suitable hosts to bind ammonium adamantane guests. While CB[7] binds Ada in water with a binding constant of log *K*_A_ = 12.23,^37,49^ β-CD forms host–guest complexes with Ada in water that are characterized by a binding constant of log *K*_A_ = 3.92.^44,50^ Both the binding of the Ada-ATRP guest to the surface-immobilized β-CD host, as well as the subsequent SI-ATRP reactions were performed in aqueous solution since the binding constants of the β-CD host–guest complexes decrease in the presence of organic solvents.^51^

**Scheme 3:**
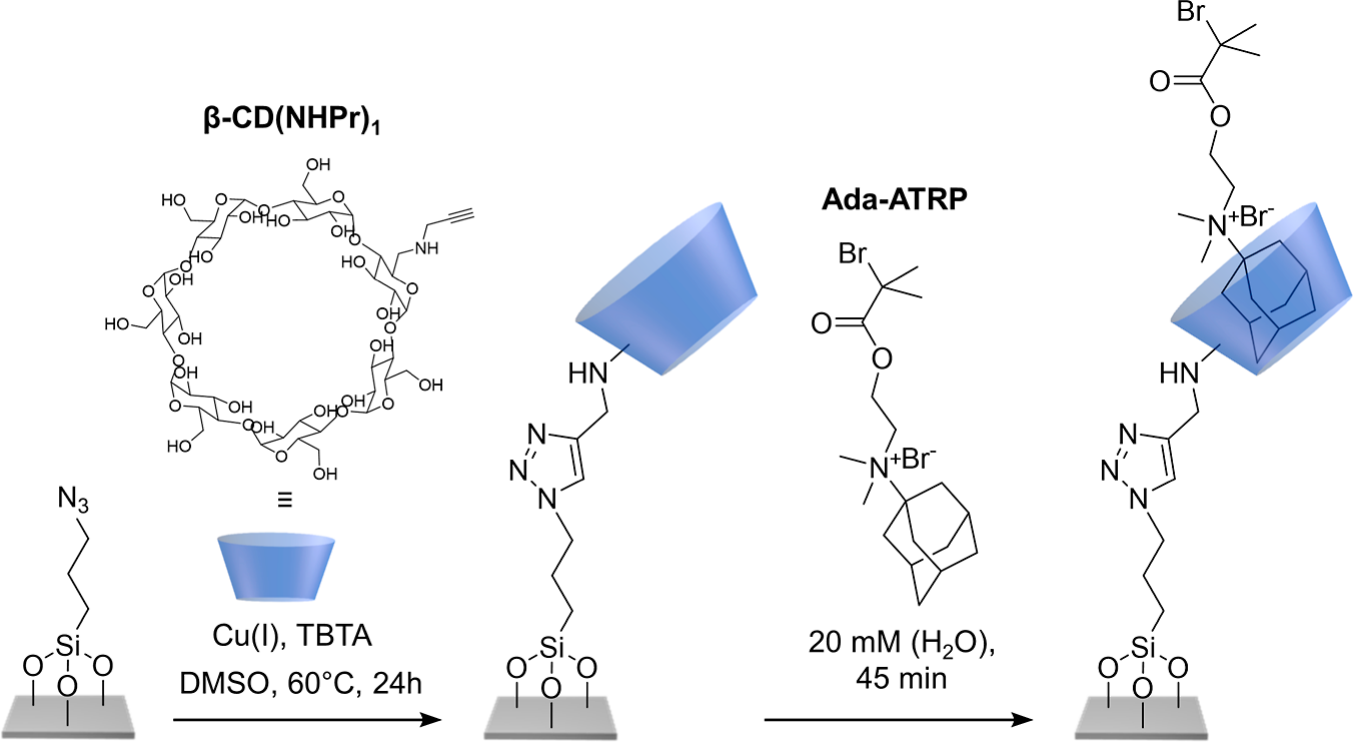
Synthesis of Surface-Attached 2-Bromoisobutyrate-Functionalized Adamantane (Ada-ATRP)—β-CD Host–Guest Complexes

**Figure 1:**
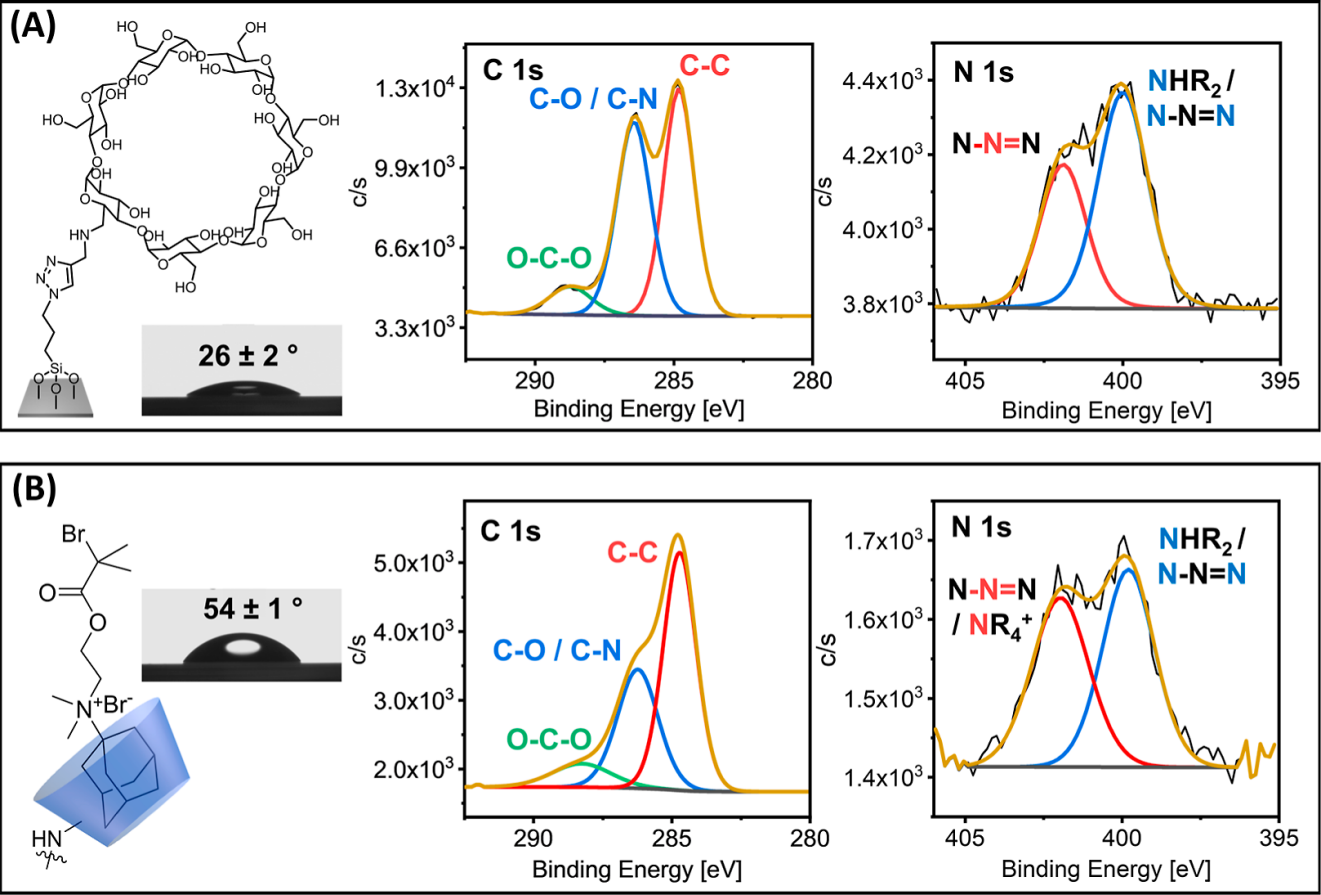
Chemical structures, water contact angles, and C 1s and N 1s XPS high resolution scans of silicon substrates that present (A) surface-attached β-CD-moieties, and (B) surface-attached Ada-ATRP@β-CD host–guest complexes.

Scheme 3 presents the preparation of the surface-anchored noncovalent Ada-ATRP@β-CD initiator. In a first step, a monopropargyl β-CD derivative (β-CD(NHPr)_1_), which was obtained following a literature procedure,^52^ was immobilized via copper-catalyzed azide–alkyne-cycloaddition on an azidopropyl triethoxysilane modified silicon wafer. After surface-attachment of the β-CD derivative, the water contact angle decreased from 63 ± 3° for the azido-modified silicon substrate to 26 ± 2° for the β-CD functionalized surface (Figure 1A), which reflects the hydrophilic character of the cyclodextrin moieties. After immobilization of the host molecule, the wafers were immersed into a 20 mM aqueous solution of Ada-ATRP for 45 min to form the surface-attached supramolecular initiator. Formation of the Ada-ATRP@β-CD complex resulted in an increase of the water contact angle to 54 ± 1° as compared to 26 ± 2° for the β-CD functionalized surface, which is attributed to the hydrophobic 2-bromoisobutyryl group that is presented at the surface (Figure 1B).

The surface attachment of β-CD, and the subsequent formation of the Ada-ATRP@β-CD complex was also monitored with XPS (Supporting Information Figures S1 and S2). Figure 1A presents high resolution C 1s and N 1s XPS scans of the β-CD(NHPr)_1_-modified silica surfaces. The corresponding survey XPS spectrum is shown in Supporting Information Figure S1A. The C 1s signal can be deconvoluted into three residuals, which can be assigned to aliphatic (C–C) carbon atoms at 284.8 eV, **C**–O/**C**–N carbon atoms at 286.3 eV and O–**C**–O carbon atoms at 288.2 eV. The experimental relative area ratio of the **C**–O/**C**–N and O–**C**–O residuals is 5.6:1, which is in agreement with the theoretical value. If all surface-bound azide groups would have reacted with the alkyne-modified β-CD(NHPr)_1_, an XPS C 1s O–**C**–O/**C**–**C** area ratio of 3.5 would be expected. Analysis of the C 1s high resolution scan in Figure 1A, however, reveals an O–**C**–O/**C**–**C** area ratio of 0.2, from which it can be estimated that ∼7.3% of the azido groups have reacted with β-CD(NHPr)_1_. Given that (3-azidopropyl)triethoxysilane has a surface area of 20 Å^2^/molecule^53^ and β-CD has a surface area of l86 Å^2^/molecule,^54^ this suggests a β-CD surface concentration that is ∼70% of the maximum possible surface concentration. The high-resolution N 1s signal of the β-CD-modified substrate can be deconvoluted into two residuals. One at 400.0 eV, which can be assigned to the secondary amine and the C–**N**=N triazole nitrogens, and a second one at 402.0 eV, which can be assigned to the N–**N**=N triazole nitrogen.

To study binding of the ATRP initiator-modified quaternary ammonium adamantane guest by β-CD, solution ^1^H NMR experiments were performed. Comparison of the ^1^H NMR spectra of β-CD, Ada-ATRP and the Ada-ATRP@β-CD host–guest complex reveals downfield shifts of the adamantane protons as well as highfield shifts of the interior β-CD protons, which is consistent with binding of the quaternary ammonium adamantane moiety of Ada-ATRP by β-CD (Supporting Information Figure S3).^55,56^ To visually monitor the formation of the surface-attached β-CD host–guest inclusion complex, a fluorescent adamantane derivative (Ada-Flu) was synthesized. As for Ada-ATRP, inclusion of the quaternary ammonium adamantane moiety of Ada-Flu by β-CD was confirmed by solution ^1^H NMR experiments that compared the NMR spectra of Ada-Flu, β-CD and the Ada-Flu@β-CD host guest complex (Supporting Information Figure S4). Fluorescent microscopy analysis of β-CD-functionalized silicon wafers, which were immersed in a 1 mM solution of Ada-Flu, washed with water, and dried, indicated the presence of the surface-bound Ada-Flu guest moieties (Supporting Information Figure S5). Silicon wafers presenting the Ada-ATRP@β-CD supramolecular initiator were also characterized by XPS. Figure 1B presents the C 1s and N 1s high resolution scans of these ATRP initiator modified surfaces. The corresponding survey XPS spectrum is shown in Supporting Information Figure S1B. In the C 1s high resolution scan, the **C**–**C**/**C**–O ratio increases from 1.1:1 to 2.1:1, indicating the presence of more aliphatic carbon species on the surface. In the N 1s region, the signal intensity at 402.0 eV increases relative to the signal at 400.0 eV, which reflects the binding of the quaternary ammonium group of Ada-ATRP.

**Scheme 4:**
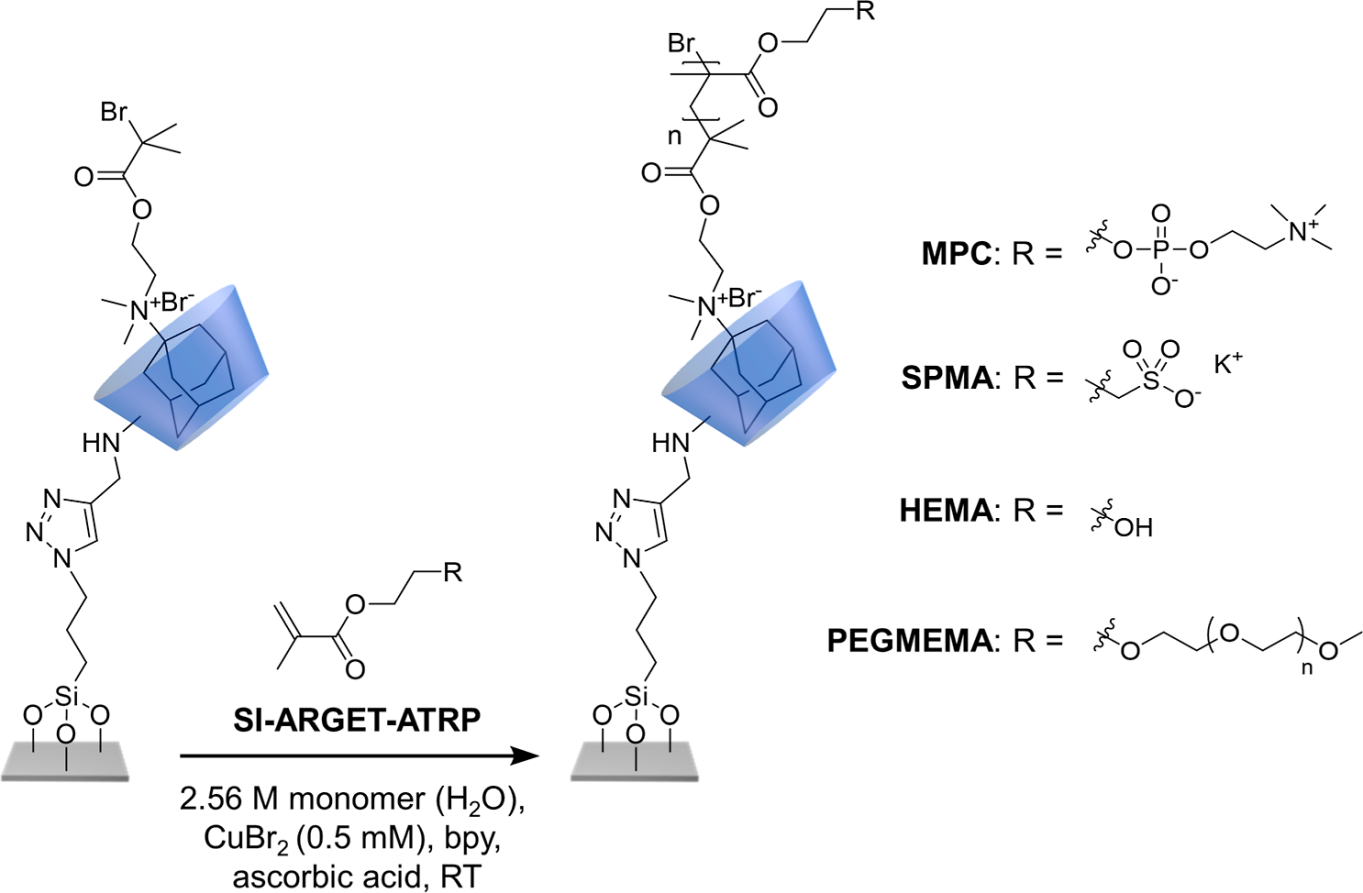
Growth of Supramolecular Polymer Brushes via SI-ARGET-ATRP From Surface-Attached Ada-ATRP@β-CD Initiators

To investigate the ability of the surface-bound Ada-ATRP@ β-CD host–guest complexes to grow polymer brushes, surface-initiated atom transfer radical polymerization experiments were performed with four water-soluble monomers, viz. 2-methacryloyloxyethyl phosphorylcholine (MPC), 3-sulfopropyl methacrylate potassium salt (SPMA), poly(ethylene glycol) methyl ether methacrylate (PEGMEMA), and 2-hydroxyethyl methacrylate (HEMA) (Scheme 4). Since the binding constants of the β-CD host–guest complexes significantly decrease in the presence of organic solvents, these polymerizations were performed in aqueous solution.^51^ These polymerizations were performed following an established activators regenerated by electron transfer (ARGET) surface-initiated atom transfer radical polymerization (SI-ATRP) protocol that uses CuBr_2_, 2,2′-bipyridine (bipy) and ascorbic acid as the catalyst system, and which has been previously successfully employed to graft polymer brushes from a variety of surfaces.^40,57^ Surface-initiated polymerizations were followed by monitoring the dry film thicknesses of the polymer brushes, which were determined using ellipsometry, as a function of the polymerization time.

**Figure 2:**
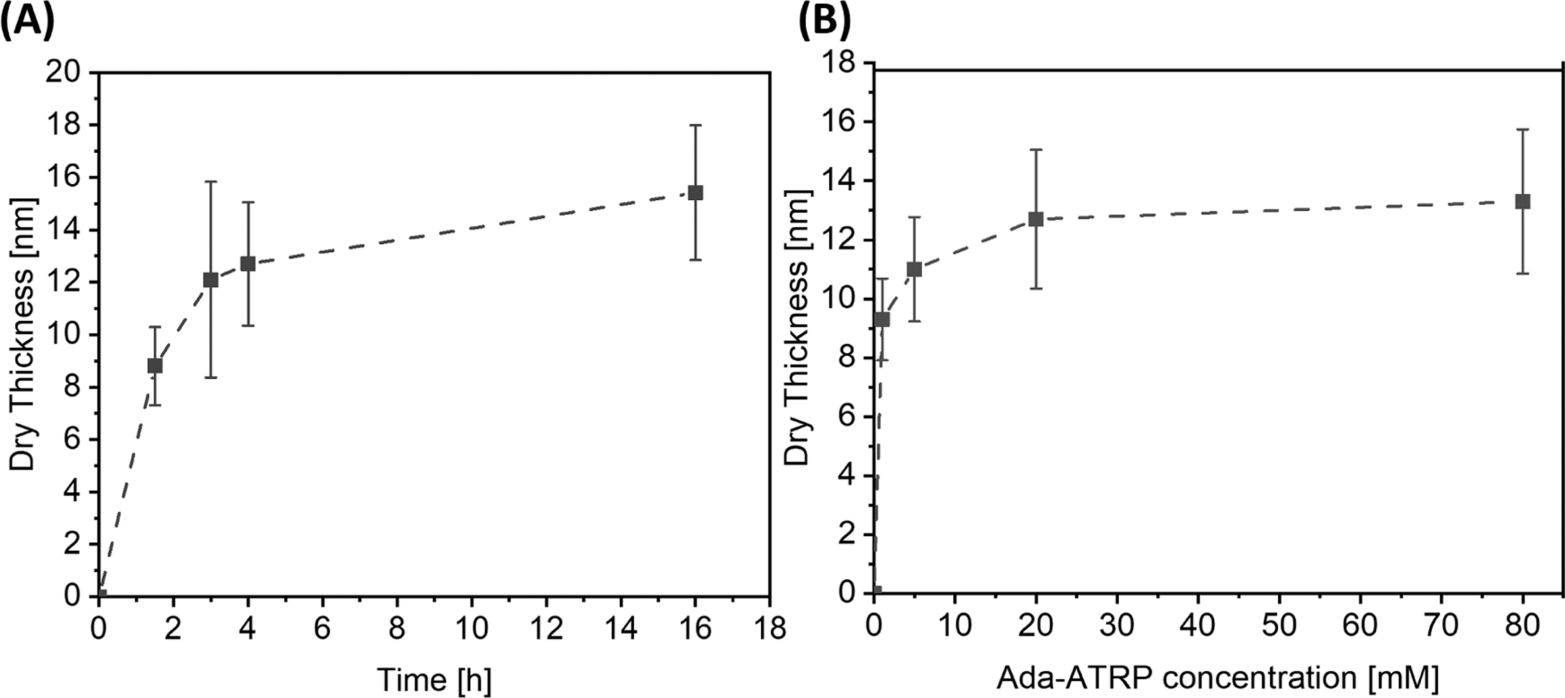
(A) Film thicknesses of PMPC brushes obtained via SI-ARGET-ATRP as a function of polymerization time using a monomer (MPC) concentration of 2.56 M from Ada-ATRP functionalized β-CD surfaces obtained with an Ada-ATRP concentration of 20 mM; (B) dry film thicknesses of PMPC brushes after 4 h of polymerization time as a function of Ada-ATRP concentration.

Figure 2A, as a representative example, shows the evolution of dry polymer brush film thickness as a function of polymerization time for the SI-ARGET ATRP of MPC from initiator-modified silicon surfaces that were obtained by exposing a β-CD-functionalized surface to a 20 mM aqueous solution of Ada-ATRP. After a rapid increase in the dry film thickness during the first 2 h, the rate of polymerization (which can be estimated from the slope of the film thickness versus polymerization time plot) decreases, and the thickness of the PMPC brushes reaches a plateau value of ∼14 nm. The evolution of dry film thickness with polymerization time illustrated in Figure 2A is characteristic for the growth of polymer brushes via SI-ATRP.^58,59^ Initially, this process allows for a linear increase in film thickness with polymerization time. At a certain point, however, this linear regime is followed by a decrease in the film growth rate, i.e. polymerization rate, and a plateauing off of the dry film thickness, which is indicative of the loss of polymerization active chain ends. Figure 2B presents the dry film thicknesses of PMPC brushes, which were grown for a period of 4 h from β-CD-functionalized surfaces that were treated with aqueous solutions containing 1–80 mM Ada-ATRP. Upon increasing the Ada-ATRP concentration that was used to prepare the Ada-ATRP@β-CD functionalized silicon substrates from 1, to 5, 20, and 80 mM, a gradual increase in PMPC film thickness from 9.3 ± 1.4 to 13.3 ± 2.5 nm was observed. As the experiments summarized in Figure 2B were conducted at identical polymerization times, the observed increase in brush thickness with increasing solution concentration of Ada-ATRP reflects the increase in ATRP initiator surface concentration, and concomitantly polymer brush grafting density as the solution concentration of Ada-ATRP is increased from 1 to 5, 20, and 80 mM. The data in Figure 2B can be fitted with a Langmuir isotherm (Supporting Information Figure S6A), which reveals a binding constant of log *K*_A_ = 2.48 ± 1.90, which is in the same range as the solution binding constant of log *K*_A_ = 3.92 ± 0.02, which has been reported for the β-CD - adamantane host–guest complex.^44^ In addition to the initiator surface concentration, the PMPC brush thickness also depends on the monomer concentration. When using initiator-modified substrates that were obtained using an Ada-ATRP solution concentration of 20 mM, a polymerization time of 4 h afforded a film thickness of 4.5 ± 0.9 nm using a monomer concentration of 0.64 M. Under the same conditions, a dry film thickness of 12.7 ± 2.4 nm was found for a monomer concentration of 2.56 M (see Supporting Information Figure S6B). This increase in film thickness with increasing monomer concentration reflects the dependence of the rate of the surface-initiated polymerization reaction on the monomer concentration.^58,59^ The ability to grow supramolecular polymer brushes via the SI-ARGET ATRP protocol that was used here, also provides a good starting point for the synthesis of these surface-grafted polymers via more refined methods, using e.g. metal free protocols,^60^ or flow-based processes.^61^

**Figure 3:**
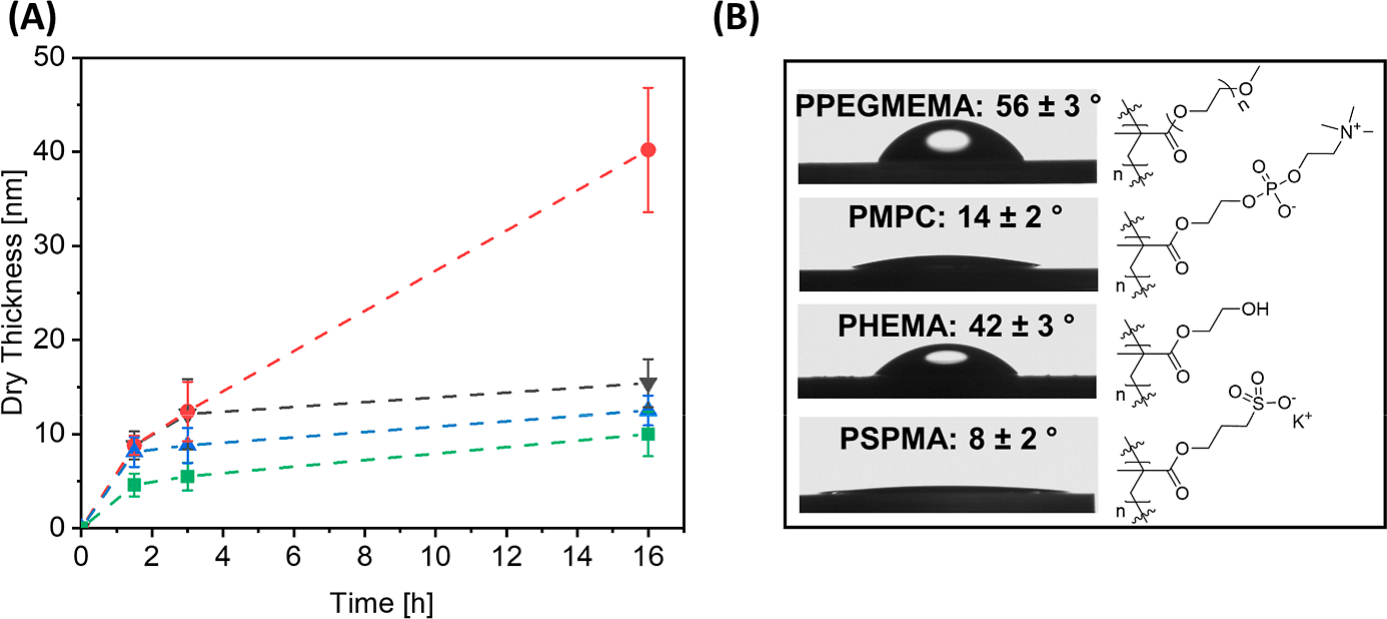
(A) Dry film thickness as a function of polymerization time for the SI-ARGET-ATRP of PEGMEMA (red ●), MPC (black ▼), HEMA (green ■) and SPMA (blue ▲) at a monomer concentration of 2.56 M; (B) chemical structures and water contact angles of PPEGMEMA (*d*_dry_ = 40 nm), PMPC (*d*_dry_ = 15 nm), PSPMA (*d*_dry_ = 13 nm), and PHEMA brushes (*d*_dry_ = 10 nm).

In addition to studying the feasibility of the surface-attached Ada-ATRP@β-CD initiators for the SI-ATRP of MPC, the grafting of polymer brushes generated from PEGMEMA, HEMA and SPMA was investigated. These experiments were performed using the same ARGET ATRP conditions that were used to synthesize PMPC brushes using a monomer concentration of 2.56 M, and surface-attached Ada-ATRP@β-CD initiators that were prepared with a 20 mM concentration of Ada-ATRP. Figure 3A compares the evolution of dry film thickness versus polymerization time for the SI-ARGET-ATRP of PEGMEMA, HEMA and SPMA with that of MPC. The slope of the initial, linear increase in film thickness as a function of polymerization time that is observed for all 4 monomers is a measure of the rate of the polymerization of the respective monomer. At a at certain polymerization time, all 4 growth profiles start to level off, which reflects a loss of living chain ends, and a loss of control over chain growth. After a polymerization time of 3 h, polymer brushes with dry film thicknesses of 5.5 nm (PHEMA), 8.8 nm (PSPMA), 12.1 nm (PMPC), and 12.4 nm (PPEGMEMA) are obtained. While the rate of polymerization for the SI-ARGET-ATRP of HEMA, SPMA and MPC decreases at longer polymerization times (reflected by an only minor further increase in the dry film thickness upon increasing the polymerization time to 16 h), the rate of polymerization for PEGMEMA remained relatively constant, and PPEGMEMA brushes with a dry film thickness of 40 nm were obtained after a polymerization time of 16 h. The supramolecular polymer brushes were characterized with water contact angle analysis and XPS. Figure 3B presents the water contact angles of the 4 polymer brushes, which are in agreement with literature values.^62−65^Supporting Information Figure S7 presents high resolution XPS scans of the supramolecular PPEGMEMA, PHEMA, PMPC, and PSPMA brushes, which are in agreement with their chemical composition.

**Figure 4:**
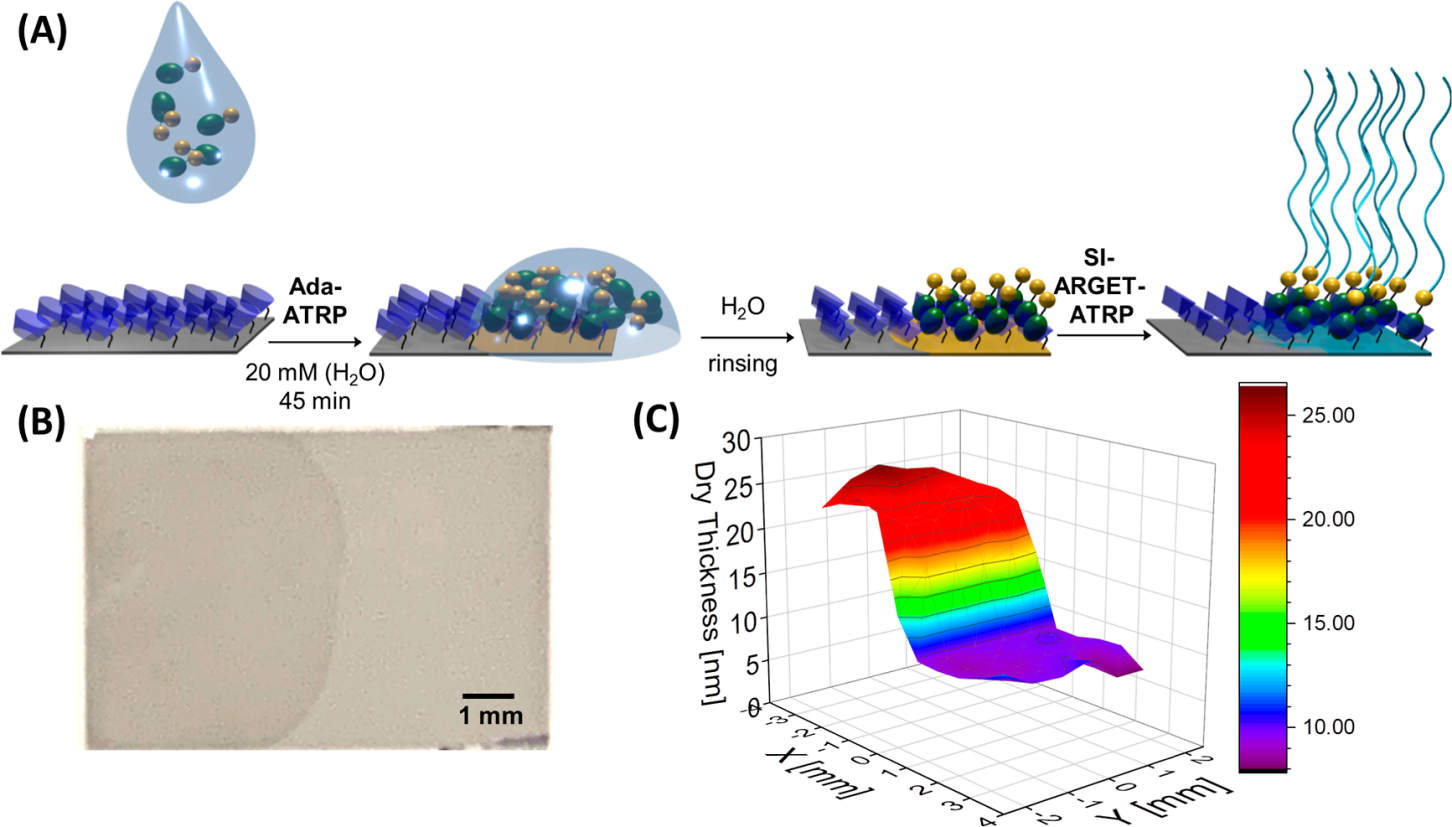
(A) Fabrication of a patterned supramolecular polymer brush via drop-casting of Ada-ATRP (20 mM in water) onto a β-CD-presenting substrate followed by ARGET-ATRP of MPC; (B) image of a 5.5 × 8 mm silicon wafer covered with a patterned PMPC brush. (C) Thickness map of the patterned PMPC brush modified wafer obtained with ellipsometry.

The supramolecular Ada@β-CD anchored polymer brushes discussed so far were grown from uniformly initiator-modified surfaces that were obtained by immersing β-CD modified silicon substrates in an aqueous Ada-ATRP solution. This process, however, also provides easy access to patterned polymer brushes. By casting a droplet of a 20 mM aqueous Ada-ATRP solution onto a β-CD modified silicon substrate, patterned ATRP initiator-modified surfaces can be prepared. Subsequent SI-ATRP from these surfaces results in the formation of patterned supramolecular polymer brushes (Figure 4A). As an example, Figure 4B shows a photomicrograph of a silicon wafer modified with a patterned PMPC brush that was prepared following the SI-ARGET-ATRP protocol described above, and a polymerization time of 16 h. In Figure 4B, the left (darker) side of the sample is the area that is modified with a PMPC brush, whereas the right (lighter) part of the sample represents the area that was not exposed to the Ada-ATRP solution. Figure 4C presents a thickness map of the full silicon wafer obtained with ellipsometry, which indicates a film thickness of ∼ 15 nm for the patterned PMPC brush.

**Figure 5:**
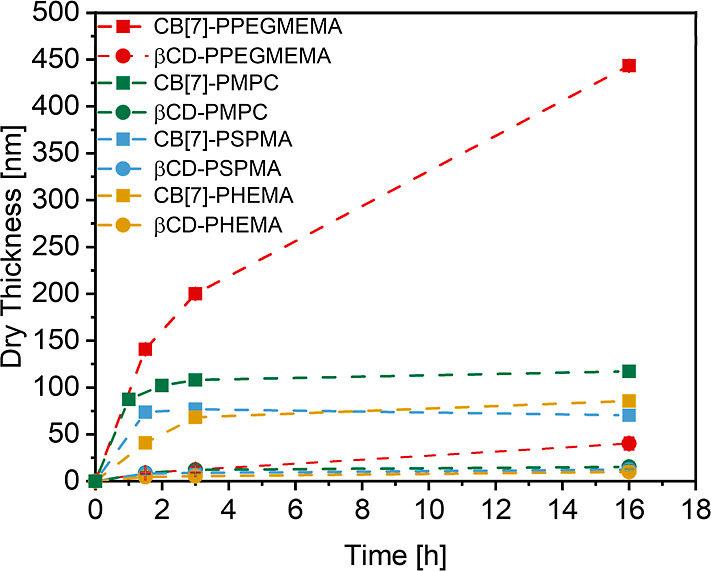
Growth profiles for the SI-ARGET ATRP of PEGMEMA, MPC, SPMA and HEMA from adamantane-functionalized ATRP initiators that are presented on CB[7]- and β-CD-modified silicon substrates.

An attractive feature of the Ada-ATRP initiator is that the quaternary ammonium adamantane moiety is able to form inclusion complexes with various hosts, which potentially allows to study the effect of the binding constant (log *K*_A_) of the host–guest complexes on the ability of these noncovalent initiators to grow polymer brushes. The results presented so far in this paper have demonstrated the feasibility of the Ada-ATRP@β-CD initiator for the synthesis of various polymer brush films in aqueous media. In a previous paper, we have shown that the Ada-ATRP initiator can also be noncovalently immobilized on cucurbit[7]uril (CB[7]) presenting surfaces, and be used to grow supramolecular PHEMA, PSPMA, PMPC and PPEGMEMA brushes.^40^Figure 5 compares the evolution of the dry film thickness as a function of polymerization time for PHEMA, PSPMA, PMPC and PPEGMEMA brushes grown from Ada-ATRP@CB[7] (taken from ref (40)), and Ada-ATRP@β-CD initiator-modified silicon substrates using the same polymerization conditions. Growth profiles that compare the polymerization of the different monomers from the β-CD and CB[7]-based initiators are presented in Supporting Information Figure S8.

**Figure 6:**
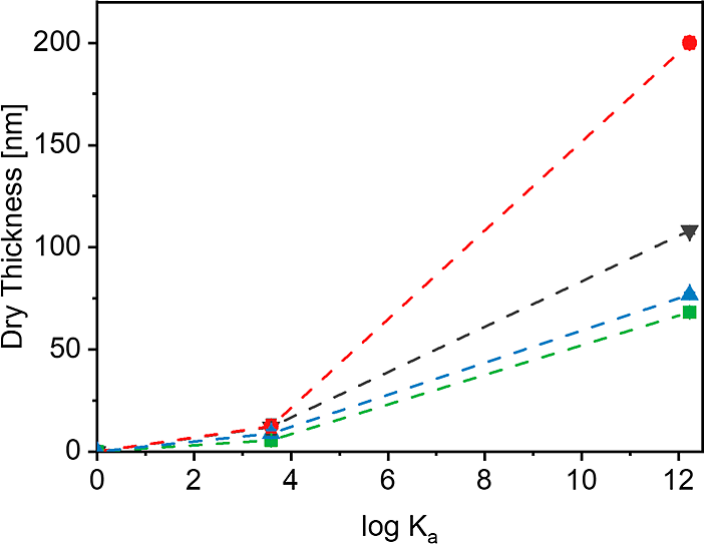
Dry film thickness vs binding constant for PPEGMEMA (red ●), PMPC (black ▼), PHEMA (green ■) and PSPMA (blue ▲) brushes prepared by SI-ARGET-ATRP from β-CD (log *K*_A_ = 3.92) and CB[7] (log *K*_A_ = 12.23)-bound noncovalent initiators using a monomer concentration of 2.56 M after a reaction time of 3 h.

The data presented in Figure 5 allow for a first assessment of the effect of log *K*_A_ of the noncovalent, surface-bound initiators on the growth of supramolecular polymer brushes via SI-ATRP. For this comparison, we will focus on the dry film thickness of brushes obtained after a polymerization time of 3 h (Table 1). Figure 6 plots these dry film thicknesses as a function of the binding constant (log *K*_A_) for the respective adamantane—host–guest complex. The 3 h time point was selected as it marks the moment where the growth profiles in Figure 5 start to plateau off, indicative of a loss of living chain ends, and a loss of control over chain growth. Since all brushes were synthesized using the same SI-ARGET ATRP protocol, we assume that surface-initiated polymerization of a given monomer from the β-CD and CB[7] initiator-modified surfaces will produce polymer grafts with comparable (number-average) molecular weights (*M*_n_). The dry film thickness (*h*) presented in Figure 6 and in Table 1 depends on the grafting density (σ) of the brush, and the number-average molecular weight (*M*_n_) of the polymer tethers as follows:^8^*h* = (σ *M*_n_)/(ρ N_A_) (where ρ is the bulk density of the polymer, and *N*_A_ is Avogadro’s number). Under the assumption that the use of identical SI-ARGET ATRP conditions generates polymer grafts of similar molecular weight from the β-CD and CB[7] initiator-modified surfaces, this implies that the differences in film thickness between these substrates reflect differences in grafting density. The data in Table 1 and in Figure 6 indicate that for a given monomer, the dry film thickness obtained for a brush generated from a CB[7]-based initiator modified surface is 5–19 times larger as compared to the dry film thickness of the same brush prepared using the β-CD-based initiator-modified surface. This suggests that the grafting densities of the β-CD initiator-grown polymer brushes are 5–19 times smaller as compared to the same brushes grown from the CB[7]-based initiator modified surfaces. Analyses of the grafting densities of PMPC brushes from swelling experiments using the self-consistent field theory developed by Milner et al. revealed σ = 0.014 ± 0.003 chains/nm^2^ for the β-CD grown brush, and 0.031 ± 0.002 chains/nm^2^ for the CB[7] grafted brush. These differences in grafting densities are striking, and small considering a difference of 8 orders of magnitude in the binding constants for the β-CD and CB[7]—adamantane host guest complexes. This highlights that the solution binding constant of the host–guest complex is not the only parameter that determines the surface concentration of the ATRP initiator (and concomitantly the polymer brush grafting density), and that other factors such as e.g. interactions between the guest and the substrate and/or the guest and the polymer may also play a role. Furthermore, the (growing) polymer brush layer may also provide a steric barrier that stabilizes the polymer film. This is due to the fact that the rates for the reversible formation and dissociation of the host–guest complexes are much faster as compared to diffusion of polymer grafts out of the polymer film. This is further illustrated by the results of experiments where β-CD and CB[7]-initiator grown PMPC brushes were incubated in Milli-Q water for 9 days (Supporting Information Figure S9). Analysis of the dry film thicknesses of the brushes only reveals a limited decrease in thickness, which is attributed to the steric hindrance imposed by the brush, which hampers diffusion of polymer grafts in the decomplexed state out of the brush. In another experiment, an Ada@CB[7] tethered PMPC brush was exposed to aqueous solutions containing up to 0.5 M of a competitive guest, viz. ammonium adamantane. Over a period of 24 h no significant decrease in film thickness was observed when the experiment was conducted at room temperature, and only a limited decrease in film thickness was observed at 60 °C (Supporting Information Figure S10), which is attributed to the fact that the polymer brush acts as a barrier for the ammonium adamantane to diffuse to the Ada@CB[7] complexes at the interface between the polymer brush and the substrate. The results in Supporting Information Figures S9 and S10 are also interesting as they illustrate that robust polymer brush films can even be grown from surfaces where the ATRP initiators are noncovalently bound using putatively weak host–guest complex interactions.

**Table 1:** Dry Film Thicknesses of Polymer Brushes Obtained via SI-ARGET-ATRP at a Polymerization Time of 3 h From Ada-ATRP Presenting β-CD and CB[7] Modified Silicon Substrates, Together With the Solution Log *K*_A_ Values of the Respective Host–Guest Complexes

host	log *K*_A_	dry film thickness after a polymerization time of 3 h (nm)			
		PHEMA	PMPC	PSPMA	PPEGMEMA
CB[7]	12.23^37,39^	68.2 ± 1.2	102.0 ± 0.9	76.8 ± 1.1	200.1 ± 3.0
β-CD	3.92^44,50^	5.5 ± 1.5	12.1 ± 3.7	8.8 ± 1.9	12.4 ± 3.1

## Conclusions

This report has described the successful use of surface-immobilized ATRP-initiator-modified Ada@β-CD host–guest complexes for the preparation of supramolecular polymethacrylate brushes. Using SI-ARGET-ATRP conditions, Ada@β-CD anchored PHEMA, PMPC, PSPMA and PPEGMEMA brushes with film thicknesses of up to 40 nm could be prepared. Furthermore, comparison of brushes grown from Ada@β-CD—based initiators with those grafted from Ada@CB[7]—based initiators under the same conditions indicated differences in grafting densities that are much smaller as expected based on the difference in the solution binding constant of these two host–guest complexes, as well as a remarkable stability of these supramolecular brushes toward detachment of the polymer grafts, in spite of the reversible nature of the host–guest complexes. These observations are attributed to the fact that the rates for the reversible formation and decomplexation of the host–guest complexes are much faster than the diffusion of the polymer chains in the decomplexed state out of the polymer brush film. As a consequence, the surface-grafted polymer brush presents a steric barrier that prevents detachment of individual polymer grafts, and also allows surface-initiated polymerization from surfaces where initiators are bound via putatively weak host–guest complexes. The fact that robustly anchored polymer brushes can be grown from host–guest complex-based, surface-immobilized initiators highlights the potential of supramolecular chemistry and self-assembly to produce initiator-modified substrates, and could pave the way to new strategies to control polymer brush grafting density, to produce patterned brushes and design reversibly tethered polymer brushes.

## Data Availability

The data underlying this study are available in the Zenodo repository at https://doi.org/10.5281/zenodo.14804108.

## Supporting Information Available

Synthetic protocols and characterization procedures, analytical data of synthesized compounds (PDF)
