# Supplementary material for: Supramolecular Polymer Brushes Grafted via Atom Transfer Radical Polymerization from Surfaces Presenting Non-covalent, Host–Guest Complex-Based Initiators
Source: Macromolecules. 2025 Mar 19;58(7):3554–63. doi: 10.1021/acs.macromol.5c00058 (PMC11984309; doi:10.1021/acs.macromol.5c00058)
Supplement: Supplementary file 1 — ma5c00058_si_001.pdf [file ma5c00058_si_001.pdf]

Supporting Information

Supramolecular Polymer Brushes Grafted via Atom Transfer Radical Polymerization from Surfaces Presenting Non-Covalent, Host – Guest Complex-Based Initiators

Friederike K. Metze[1,2] *and Harm-Anton Klok*[1,2]*

[1] École Polytechnique Fédérale de Lausanne (EPFL), Institut des Matériaux and Institut des Sciences et Ingénierie Chimiques, Laboratoire des Polymères, Bâtiment MXD, Station 12, CH-1015 Lausanne, Switzerland

[2] National Center of Competence in Research Bio-inspired Materials, Chemin des Verdiers 4, CH-1700, Fribourg, Switzerland

CORRESPONDING AUTHOR: harm-anton.klok@epfl.ch; Tel: + 41 21 693 4866

1. EXPERIMENTAL SECTION

1.1. MATERIALS

All chemicals were used without further purification unless described otherwise. Acetic acid (≥ 99%), 1-adamantylamine (97%), 1-amantadine hydrochloride (100%), 2-bromoethanol (95%), α-bromoisobutyryl bromide (98%), copper(I)bromide (99.995+%), copper(II)bromide (99.999 %), dimethyl formamide (99.8%), dimethyl sulfoxide (≥ 99%), formaldehyde (37% wt. in H_2O), formic acid (≥ 95%), glycerol (≥ 99.5%), glycoluril (97%), 2-hydroxyethyl methacrylate (HEMA), hydrochloric acid (37%), (L)-ascorbic acid (99%), 2-methacryloyloxyethyl phosphorylcholine (MPC) (97%), methanol (anhydrous, 99.8%), paraformaldehyde (95%), N,N,N′,N″,N″-pentamethyldiethylenetriamine (PMDETA) (99%), poly(ethylene glycol) methyl ether methacrylate (PEGMEMA) (average M_n 300), potassium peroxodisulfate (≥ 99.0%), propargyl bromide (80% wt. in toluene), p-tolylsulfonyl chloride (≥ 99%), pyridine (≥99.0%), sodium hydride (60% wt. dispersion in mineral oil), 3-sulfopropyl methacrylate potassium salt (SPMA) (98%), and tetrabutylammonium bromide (TBAB) (≥ 99%) were purchased from Sigma-Aldrich. 2,2′-Bipyridine (bipy) (99%), β-cyclodextrin (95%), fluorescein amine isomer I (96%), propargyl amine (95%) and tris((1-benzyl-1H-1,2,3-triazol-4-yl)methyl)amine) (TBTA) (97%) were purchased from ABCR. (3-Chloropropyl)triethoxysilane (99%) was purchased from Chemie Brunschwig. Potassium sulfate (≥ 99.0%) and sodium chloride (≥ 95%) were purchased from Fluka. Magnesium sulfate, sodium hydroxide and sodium sulfate were purchased from Reactolab SA. Ammonia (25%) was purchased from VWR. Prior to polymerization, the inhibitor was removed from liquid monomers by passing through a basic aluminium oxide column. In case of MPC, the inhibitor was removed by washing with cold acetonitrile. MilliQ water was purified with a Millipore Direct-Q 5 ultrapure water system. Toluene and dichloromethane (DCM) were dried and purified using a solvent purification system (PureSolv). Acetonitrile and diethyl ether were

dried by storing over 3 Å molecular sieves for at least 48 hrs. DMSO was dried by first, pre-drying over magnesium sulfate and second, drying twice over 3Å molecular sieves for a minimum of 48 hrs. Surface modifications were performed using silicon wafers or fused silica wafers (5.5 mm × 8 mm), covered by a ~ 2 nm silicon oxide layer. Silicon wafers were used to grow polymer brushes, and transparent fused silica wafers were used for fluorescence microscopy experiments.

1.2 METHODS

X-ray photoelectron spectroscopy (XPS). XPS analyses were carried out on a VersaProbe II instrument (Physical Electronics Inc) using the monochromated Kα X-ray line of an Aluminium anode. The pass energy was set to 46.95 eV with a step size of 0.2 eV. The samples were electrically isolated from the sample holder and charges were compensated. The spectra were referenced at 284.8eV using the C-C bound component of the C1s transition.

Fluorescence microscopy. Fluorescence microscopy images were taken with a Leica DM5500 Upright Microscope using the black and white camera DFC300G and an exposure time of 3 s. Fluorescence intensity was observed in the green channel. ImageJ was used to process microscopy images and to measure intensity values.

Ellipsometry. Dry film thicknesses were measured using a SemiLAB SE2000 instrument at an incident angle of 70°. The film thickness was fitted using a four-layer silicon/silicon oxide/polymer brush/air model. The refractive index n was given by the Cauchy approximation ($n = An + Bn/\lambda^2$), where λ represents the wavelength of the incident light. The following refractive indices were used for the fitting of the polymer brushes: PMPC (n = 1.4778),[1] PPEGMEMA (n = 1.46),[2] PHEMA (n = 1.51),[3] PSPMA (n = 1.46).[4] Per wafer, 25 points were measured and results were given as average ± standard deviation (SD) of these 25 values.

Thickness maps of the modified wafers were prepared by measuring the intersection points of 10 x 6 rows per wafer. The ellipsometry data was analyzed with the SAM suite software from SemiLAB. Reported film thicknesses are corrected for the silicon oxide layer (d(SiO_x) = 2 nm and the thickness of the respective host molecule monolayer (d(β-CD) = 4.9 ± 0.5 nm and d(CB[7]) = 3.6 ± 0.3 nm).

Water contact angle analysis (WCA). Water contact angles were determined using a DataPhysics OCA 35 instrument. For each measurement, a 5 µL water droplet was used. The contact angle was determined using the SCA20 software from DataPhysics. Final values are given as average value of all performed experiments ± SD.

Nuclear Magnetic Resonance (NMR) spectroscopy. ^{1}H NMR and ^{13}C NMR spectra were recorded on a Bruker AVANCE-400 Ultra Shield spectrometer, or on a Bruker AVANCEIIIHD-600 Ultra Shield spectrometer. Deuterated solvents (water (D_2O), dimethylsulfoxide (DMSO-*d*6), chloroform ($CDCl_3$) and methanol (MeOD-*d*4) were used and chemical shifts (δ) were measured against the residual ^{1}H proton peak of the solvent. NMR data was analysed using MNova from Mestrelab.

Matrix-Assisted Laser Desorption Ionisation-Time-of-Flight Mass Spectrometry (MALDI-TOF-MS). MALDI-TOF-MS analysis was performed using an Autoflex Speed II instrument from Bruker operated in positive reflection mode. In the case of β-CD$(NHPr)_1$, the product was diluted with water/acetonitrile 50:50 (v/v) at a concentration of 1 mg / 100 µL and the supernatant was used for the analysis. In the case of CB[7], CB[7]$(OH)_1$ and CB[7]$(OPr)_1$, the samples were diluted with water at a concentration of 1 mg / 100 µL. The matrix was α-cyano-4-hydroxycinnamic acid, which was mixed with acetonitrile/water/trifluoroacetic acid 50:49.9:0.1 (v/v). Sample/matrix were deposited as a 1:10 solution on the target.

High Resolution Mass Spectrometry (HR-MS). For HR-MS analyses, a Xevo G2-S QTOF mass spectrometer coupled to the Acquity UPLC Class Binary Solvent Manager and BTN Sample Manager (Waters, Corporation, Milford, MA) was used. Mass spectrometer detection was performed with positive ionization by using the ZSpray™ dual-orthogonal multimode ESI/APCI/ESCi® source. Samples were diluted in methanol acidified with 0.1% formic acid. The TOF mass spectra were acquired in the resolution mode over the range of m/z 50-1200. A mass accuracy better than 5 ppm was achieved using a leucine-encephalin solution as lock-mass infused continuously using the LockSpray source. Data were processed using MassLynx™ 4.1 software.

1.3 PROCEDURES

Synthesis of 3-azidopropyltriethoxysilane. 3-Azidopropyltriethoxysilane was synthesized following a slight modification of the procedure reported by Grim et al.[5] Sodium azide (0.39 g, 6.0 mmol, 1.5 eq) and TBAB (0.278 g, 0.8 mmol, 0.2 eq) were dissolved in 15 mL dry acetonitrile and 3-chloropropyl triethoxysilane (0.964 mL, 4.0 mmol, 1 eq) was added. The reaction mixture was stirred at 80 °C under nitrogen atmosphere for 48 hrs, and afterwards cooled down to room temperature. Diethyl ether (30 mL) was added, the mixture was filtered, and the solid residue washed with diethyl ether. The solvent was removed from the filtrate under reduced pressure and the crude product was passed through a syringe filter to remove residual solids and the pure product was obtained in form of a colorless liquid. Yield: 0.72 g (73%). ^{1}H NMR (400 MHz, $CDCl_3$): δ = 3.76 (q, J = 7.0 Hz, 6H,), 3.20 (t, J = 7.0 Hz, 2H), 1.71 – 1.57 (m, 2H), 1.16 (t, J = 7.0 Hz, 9H,), 0.67 – 0.55 (m, 2H) ppm (**Figure S11**). ^{13}C NMR (101 MHz, $CDCl_3$): δ = 58.45, 53.83, 22.66, 18.27, 7.61 ppm (**Figure S12**).

***N,N*-Dimethyladamantan-1-amine.** N,N-dimethyladamantan-1-amine was synthesized following the procedure of Guo et al.[6] Yield: 1.44 g (80%). ^{1}H NMR (400 MHz, $CDCl_3$): δ = 2.20 (d, *J* = 1.0 Hz, 6H), 2.07 – 1.97 (m, 3H), 1.62 (d, *J* = 3.0 Hz, 6H), 1.54 (ddt, *J* = 13.8, 11.5,

3.2 Hz, 6H) ppm (**Figure S13**). ^{13}C NMR (101 MHz, $CDCl_3$): δ = 52.50, 37.0, 36.08, 35.89, 28.58 ppm (**Figure S14**).

***N*-(2-Hydroxyethyl)-*N,N*-dimethyladamantan-1-aminium (Ada-OH).** Ada-OH was synthesized following a slight modification of the procedure reported by Guo et al.[6] *N,N*-dimethyladamantan-1-amine (1.40 g, 7.8 mmol, 1 eq) was dissolved in 11 mL isopropanol at 45 °C. Then, over a period of 50 min, 1-bromoethanol (3.50 g, 2.21 mL, 31.2 mmol, 4 eq) was added, and afterwards, the mixture was stirred at 84 °C for 40 hrs. After cooling to room temperature, the product was precipitated in cold acetone, filtered and washed with cold acetone. The product was obtained in form of colorless crystals. Yield: 1.21 g (51%). ^{1}H NMR (400 MHz, DMSO-*d6*): δ = 5.32 (t, *J* = 4.8 Hz, 1H), 3.89 (td, *J* = 7.0, 6.0, 3.5 Hz, 2H,), 3.33 – 3.25 (m, 2H), 2.94 (s, 6H), 2.23 (s, 3H), 2.01 (d, *J* = 3.1 Hz, 6H), 1.69 – 1.57 (m, 6H) ppm (**Figure S15**). ^{13}C NMR (101 MHz, DMSO): δ = 74.59, 59.22, 55.98, 44.54, 35.05, 34.04, 30.21 ppm (**Figure S16**).

***N*-(2-((2-Bromo-2-methylpropanoyl)oxy)ethyl)-*N,N*-dimethyladamantan-1-aminium (Ada-ATRP).** Ada-OH (1.00 g, 3.29 mmol, 1 eq) was dissolved in 50 mL dry acetonitrile, and pyridine (0.78 g, 0.80 mL, 9.87 mmol, 3 eq) was added. Then, the solution was stirred for one hour at 0 °C. α-Bromoisobutyryl bromide (3.02 g, 1.62 mL, 13.15 mmol, 4 eq) in 4 mL dry acetonitrile was added dropwise over a period of 1 hr at 0 °C under nitrogen atmosphere. Afterwards, the reaction mixture was allowed to reach room temperature, and was stirred for 24 hrs. The solvent was removed *in vacuo* and the crude product was dispersed in THF, filtered and washed with THF. The byproduct pyridine · HBr was removed via sublimation. The product was further purified via column chromatography with acetonitrile/water 20:1 (v/v) as eluent and silica gel as stationary phase. Yield: 0.69 g (46%). ^{1}H NMR (400 MHz, D_2O): δ = 4.68 – 4.59 (m, 2H), 3.79 – 3.59 (m, 2H), 2.95 (s, 6H), 2.26 (s, 2H), 2.04 (d, *J* = 3.1 Hz, 6H), 1.88 (s, 3H), 1.63 (q, *J* = 13.0 Hz, 3H) (**Figure S17**). ^{13}C NMR (101 MHz, D_2O): δ = 172.57,

76.70, 59.99, 55.97, 55.77, 43.99, 34.49, 34.10, 30.28, 29.55 ppm (**Figure S18**). MS (ES+): *m/z* calcd. for $C_{18}H_{31}BrNO_2^+$ = 372.15, found 372.15 (**Figure S19**).

***N,N*-dimethyl-*N*-(prop-2-yn-1-yl)adamantan-1-aminium**

Ada(OPr)$_1$). *N,N*-Dimethyladamantan-1-amine (300 mg, 1.7 mmol, 1 eq) and 600 mg propargyl bromide (80 wt.-% in toluene, 0.75 g, 5.1 mmol, 3 eq) were heated to 50 °C in 3 mL acetonitrile under nitrogen atmosphere and stirred for 9 hrs. After cooling down to room temperature, addition of 4 mL tetrahydrofuran precipitated a white solid, which was separated via filtration and washed with 100 mL of tetrahydrofuran. The product was obtained in form of a colourless powder. Yield: 0.25 g (49%). ^{1}H NMR (400 MHz, DMSO-*d*6): δ = 4.31 (d, *J* = 2.5 Hz, 2H), 4.02 (d, *J* = 2.5 Hz, 1H), 2.97 (s, 6H), 2.23 (s, 3H), 2.05 (d, *J* = 3.1 Hz, 6H), 1.63 (d, *J* = 3.1 Hz, 6H) ppm (**Figure S20**). ^{13}C NMR (101 MHz, DMSO) δ: = 83.19, 75.00, 74.31, 49.54(N-$\underline{C}H_2$), 45.31, 34.90, 34.64, 30.30 ppm (**Figure S21**).

5-Azidofluorescein. 5-azidofluorescein was prepared following the protocol by Shieh et al.[7]. Yield: 104 mg (56%). ^{1}H NMR (400 MHz, DMSO-d_6): δ = 10.15 (s, 2H), 7.65 (d, *J* = 2.1 Hz, 1H), 7.52 (dd, *J* = 8.1, 2.2 Hz, 1H), 7.30 (d, *J* = 8.3 Hz, 1H), 6.68 (d, *J* = 2.3 Hz, 2H), 6.64 – 6.52 (m, 4H) ppm (**Figure S22**). ^{13}C NMR (101 MHz, DMSO): δ = 168.27, 160.02, 152.34, 149.22, 142.25, 129.54, 128.45, 127.28, 126.11, 114.85, 113.10, 109.77, 102.72, 83.77 ppm (**Figure S23**).

Ada-Flu. 5-azidofluorescein (100 mg, 0.26 mmol, 1 eq), Ada(OPr)$_1$, (200 mg, 1.34 mmol, 5 eq) and PMDETA (45 mg, 0.26 mmol, 1 eq) were dissolved in 28 mL of a 13 : 1 (v/v) dimethylformamide/water mixture. After degassing via four freeze-pump-thaw cycles, copper(I)bromide (38 mg, 0.26 mmol, 1 eq) was added, and the reaction mixture was stirred at room temperature under nitrogen atmosphere for three days. The solvent was removed *in vacuo* and 50 mL tetrahydrofuran were added. The solid was separated via centrifugation. Again, the

solid was washed with 50 mL tetrahydrofuran, followed by centrifugation. The crude product was dried *in vacuo*. Afterwards, the solid was dispersed in 3 mL of methanol and purified with a silica column using methanol as eluent. The product was obtained in form of an orange powder. Yield: 59 mg (33 %). ^{1}H NMR (400 MHz, MeOD): δ = 8.91 (s, 1H), 8.35 (d, J = 2.3 Hz, 1H), 8.03 (dd, J = 8.3, 2.3 Hz, 1H), 7.38 (d, J = 8.3 Hz, 1H), 7.00 (d, J = 9.7 Hz, 2H), 6.47 (dq, J = 5.1, 2.3 Hz, 4H), 4.61 (s, 2H), 2.86 (s, 6H), 2.31 (s, 3H), 2.20 (d, J = 3.1 Hz, 6H), 1.73 (s, 6H) ppm (**Figure S24**). ^{13}C NMR (151 MHz, MeOD): δ = 170.84, 158.81, 136.98, 136.96, 134.03, 131.30, 130.71, 126.60, 122.49, 120.88, 120.46, 103.06, 75.43, 51.86, 48.02, 47.88, 47.74, 47.59, 47.45, 47.31, 47.17, 43.23, 34.78, 34.59, 30.73 ppm (**Figure S25**). MS (ES+): *m/z* calcd. for $C_{35}H_{35}N_4O_4^+$ = 591.26, found 591.30 (**Figure S26**).

Cucurbit[7]uril (CB[7]). CB[7] was synthesized using a modification of the protocol reported by Gomes et al.[8] Glycoluril (50 g, 0.35 mol, 1 eq) and paraformaldehyde (22.5 g, 0.75 mol, 2.1 eq) were mixed well in the powder state. Then, hydrochloric acid (37%, 70 mL) was added and the mixture was heated to 100 °C while stirring. The reaction mixture was kept at this temperature for 24 hrs and afterwards cooled down to room temperature. Water (800 mL) was added and the insoluble white solid (CB[6] and CB[8]) was removed via filtration. To the filtrate, 800 mL methanol was added to precipitate CB[5] and CB[7], which were isolated by centrifugation. The precipitate was dried, dissolved in approximately 250 mL water, filtered and again precipitated with 800 mL of methanol followed by centrifugation. The residue, containing mostly CB[7] as well as some CB[5] was dried and dissolved in 20% hot glycerol (100 mL) to remove any residual CB[5] from the mixture, filtered and precipitated with methanol followed by centrifugation. The precipitate was dried, dispersed in dimethyl sulfoxide, centrifuged and washed with methanol to remove residual impurities. As last step, the dried precipitate was again dissolved in water, filtered and precipitated in methanol, followed by centrifugation and 2x washing with methanol. Finally, the product was dried at

110°C overnight under vacuum. Yield: 4.56 g (8 %). ^{1}H NMR (400 MHz, D_2O): δ = 5.71 (d, *J* = 15.3 Hz, 14H), 5.44 (s, 14H), 4.15 (d, *J* = 15.3 Hz, 14H) ppm (**Figure S27**). ^{13}C NMR (151 MHz, D_2O): δ = 156.49, 71.19, 52.47 ppm (**Figure S28**). MALDI-TOF: m/z calcd. for $C_{42}H_{42}N_{28}O_{14}H[M+H]^+$: 1163.35, found: 1163.35 (**Figure S29)**.

Monohydroxy-cucurbit[7]uril (CB[7](OH)$_1$). CB[7](OH)$_1$ was prepared using a modification of the protocol by Ahn et al.[9] First, CB[7] (2.5 g, 1.75 mmol, 1 eq) and potassium sulfate (1.85 g, 10.5 mmol, 6 eq) were dissolved in 250 mL water, and potassium peroxodisulfate (375 mg, 1.4 mmol, 0.8 eq) was added. The mixture was degassed with three freeze-pump-thaw cycles. Afterwards, the reaction mixture was stirred at 85° C for 12 hrs. After cooling down to room temperature, the solvent was removed under reduced pressure and the solid residue was extracted with 50 mL HCl (37 %). After removal of the insoluble material via filtration, the product, containing unreacted CB[7] and CB[7](OH)$_n$, was precipitated from the filtrate with 200 mL methanol. After centrifugation and washing with methanol, the crude product was dried under vacuum. Pure CB[7](OH)$_1$ was separated from CB[7] and other CB[7](OH)$_n$ via column chromatography using water/acetic acid/formic acid 10:10:1.5 (v/v) as eluent and silica gel as stationary phase. Per column, around 1.5 g of crude product was dissolved in a minimum amount of eluent. The combined CB[7](OH)$_1$ fractions were dried under reduced pressure, and the pure product was triturated with methanol followed by centrifugation and drying under high vacuum. Yield: 322 mg (12%). ^{1}H NMR (400 MHz, D_2O + NaCl): δ = 5.77 (dd, J = 28.9, 15.5 Hz, 12H), 5.63 – 5.46 (m, 14H), 5.34 (s, 1H), 4.54 (d, J = 15.6 Hz, 2H), 4.29 (dd, J = 15.8, 10.7 Hz, 12H) ppm (**Figure S30**). ^{13}C NMR (151 MHz, D_2O + NaCl): δ = 156.83, 155.23, 93.58, 77.62, 71.19, 52.49, 46.62 ppm (**Figure S31**). MALDI-TOF: m/z calcd. for $C_{42}H_{42}N_{28}O_{15}Na[M+Na]^+$: 1201.33, found 1201.33; m/z calcd. for $C_{42}H_{42}N_{28}O_{15}K[M+K]^+$: 1217.30, found: 1217.13 (**Figure S32**).

Monopropargyl-cucurbit[7]uril ($CB[7](OPr)_1$). $CB[7](OPr)_1$ was prepared using a modification of the protocol by Zhang et al.[10] $CB[7](OH)_1$ (60 mg, 51 µmol, 1 eq) was dissolved in 12 mL anhydrous dimethyl sulfoxide, and sodium hydride (60 % dispersion in mineral oil, 57 mg, 1.37 mmol, 27 eq) was added under nitrogen atmosphere and the mixture was stirred for 3 h at room temperature. Then, the reaction mixture was cooled with an ice bath to 0 °C, propargyl bromide (80 % solution in toluene, 1.92 mL, 25.3 mmol, 500 eq) was added, and the mixture was stirred at room temperature for 12 hrs. After that, 90 mL diethyl ether and 10 mL methanol were added, the mixture was ultrasonicated for 3 minutes and the resulting precipitate was separated from the supernatant via centrifugation. The brown solid was triturated twice with 50 mL methanol, and once with 50 mL diethyl ether for several hours and finally dried under vacuum. The product, a light-brown powder, was exposed to the same reaction conditions for a second time, not using 12 mL anhydrous dimethyl sulfoxide, but 7.5 mL instead. The final product was obtained in quantitative yield in form of a light-brown powder. ^1H NMR (600 MHz, D_2O + NaCl): δ = 5.77 (dd, J = 28.9, 15.5 Hz, 12H), 5.63 – 5.46 (m, 14H), 5.34 (s, 1H), 4.54 (d, J = 15.6 Hz, 2H), 4.29 (dd, J = 15.8, 10.7 Hz, 12H) ppm (**Figure S33**. MALDI-TOF: m/z calcd. for $C_{45}H_{44}N_{28}O_{15}Na[M+Na]^+$: 1239.34, found 1239.30; m/z calcd. for $C_{45}H_{44}N_{28}O_{15}K[M+K]^+$: 1255.31, found 1255.27 (**Figure S34**).

Mono-6^A-(p-tolylsulfonyl)-β-cyclodextrin (β-$CD(OTs)_1$). The synthesis was performed following the protocol reported by Guo et al.[11] The product (6.4 g, yield 28%) was obtained in form of a colorless powder. ^1H NMR (400 MHz, DMSO-d6): δ = 7.87 – 7.65 (m, 2H), 7.43 (t, J = 9.0 Hz, 2H), 5.90 – 5.55 (m, 14H), 4.89 – 4.70 (m, 7H), 4.47 (dtd, J = 23.6, 5.6, 3.3 Hz, 5H), 4.39 – 4.25 (m, 2H), 4.19 (dd, *J* = 11.2, 6.6 Hz, 1H). 3.82 – 3.40 (m, 27H), 3.33 (m, 25H (including water peak)), 2.43 (d, J = 7.0 Hz, 3H) ppm (**Figure S35**). ^{13}C NMR (151 MHz, DMSO-d_6): δ = 145.29, 133.10, 133.06, 133.02, 130.40, 130.36, 130.34, 128.06, 128.03, 128.01, 102.69, 102.49, 102.44, 102.39, 102.37, 102.30, 101.74, 73.52, 73.41, 73.19, 73.14,

72.93, 72.90, 72.88, 72.82, 72.63, 72.54, 72.52, 72.48, 72.45, 72.32, 70.19, 69.35, 60.35, 60.24, 59.95, 59.69, 21.68, 21.66 ppm (**Figure S36**).

Mono-6^A-*N*-propargylamino-6^A-deoxy-β-cyclodextrin (β-CD(NHPr)$_1$). The synthesis was performed following the a modification of the protocol reported by Guo et al.[11] Mono-6A-(p-tolylsulfonyl)-β-cyclodextrin (1.0 g, 0.8 mmol, 1 eq), used without further purification, was stirred in 2 mL propargylamine (1.72 g, 31 mmol, 39 eq) at 68 °C under N_2 atmosphere for 24 hrs. Then, the mixture was poured into 10mL acetonitrile (ACN) to precipitate the product, which was then recrystallized in methanol, and further purified using column chromatography with silica gel as stationary phase and acetonitrile / H_2O / NH_3 (25% in H_2O) 10:5:1.5 as mobile phase to afford 178 mg product (yield 19 %). ^1H NMR (600 MHz, DMSO-d6): δ = 5.87 – 5.62 (m, 14H), 4.83 (p, J = 4.0 Hz, 7H), 4.61 – 4.39 (m, 6H), 3.76 – 3.51 (m, 26H), 3.41 – 3.24 (m, 26H (including water peak)), 3.03 (d, J = 2.4 Hz, 1H), 2.95 (d, J = 12.0 Hz, 1H), 2.72 (dd, J = 12.5, 6.8 Hz, 1H) ppm (**Figure S37**). ^{13}C NMR (151 MHz, DMSO): δ = 102.74, 102.41, 102.28, 84.13, 83.72, 82.03, 81.92, 81.82, 81.76, 74.05, 73.49, 73.35, 72.93, 72.86, 72.55, 72.48, 72.40, 71.11, 60.35, 60.16, 48.62, 38.17 ppm (**Figure S38**). MALDI-TOF: m/z calcd. for $C_{45}H_{72}NO_{34}Na[M+Na]^+$: 1194.03, found 1194.54. m/z calcd. for $C_{45}H_{72}NO_{34}K[M+K]^+$: 1210.14, found 1210.50 (**Figure S39**).

Surface functionalization of silicon wafers with 3-azidopropyltriethoxysilane. 22 silicon wafers, placed in a Teflon holder, were washed with ethanol, acetone, water and again ethanol for 5 min respectively in an ultrasonic bath and dried for 1 hr under vacuum. Afterwards, they were treated with oxygen plasma using a Femto plasma cleaner at 200W (Diener Electronic) for 20 min, and then the 22 wafers were immediately immersed into a 30 mM solution of (3-azidopropyl)triethoxysilane in 20 mL dry toluene, and stirred at 400 rpm under nitrogen atmosphere for 24 hrs at room temperature. Finally, the wafers were washed with toluene and ethanol and dried under a flow of nitrogen and under vacuum.

Surface immobilization of β-CD(NHPr)$_1$ and CB[7](OPr)$_1$. β-CD-(NHPr)$_1$ (56 mg, 48 μmol, 1 eq) or CB[7](OPr)$_1$ (62 mg, 48 μmol, 1 eq) was dissolved in 16 mL anhydrous dimethyl sulfoxide, and TBTA (64 mg, 120 μmol, 2.4 eq) was added. The solution was degassed with three freeze-pump-thaw cycles, copper(I)bromide (17.0 mg, 120 μmol, 2.4 eq) was added and the Cu(I)-TBTA complex was formed while ultrasonicating for 3 min. The solution was transferred into the reactor containing 22 azide-modified wafers in a Teflon holder, which was previously purged with nitrogen for 30 min. The solution was stirred at 400 rpm at 60 °C for 24 hrs and additionally ultrasonicated for 10 s every few hours (in total 4 times). Afterwards, the solution was cooled down and the wafers were washed with dimethyl sulfoxide, ultrasonicated in a 10 mM solution of CB[7] in the case of the immobilization of CB[7](OPr)$_1$. Afterwards the wafers were ultrasonicated in water for 10 seconds, washed with water and ethanol and dried under a flow of nitrogen.

Formation of surface-immobilized Ada-Flu@CB[7] and Ada-Flu@β-CD complexes. In the case of CB[7], one CB[7]-modified wafer was immersed into 1 mL of a 1 mM aqueous solution of Ada-Flu, ultrasonicated in this solution for 20 s and rinsed with water and ultrasonicated in pure water for 3 s and dried under a flow of nitrogen and under vacuum. In the case of β-CD, one β-CD-modified wafer was immersed into 1 mL of a 1 mM aqueous solution of Ada-Flu, ultrasonicated in this solution for 20 s and rinsed with water and ultrasonicated in pure water for 3 s and dried under a flow of nitrogen and under vacuum.

Formation of surface-immobilized Ada-ATRP@CB[7] and Ada-ATRP@β-CD complexes. In the case of CB[7], the Teflon holder containing 22 CB[7]-modified wafers was immersed into 20 mL of a 5 mM aqueous solution of Ad-ATRP for 45 min, ultrasonicated in this solution for 20 s and rinsed with water and ultrasonicated in pure water for 20 s and dried under a flow of nitrogen and under vacuum. In the case of β-CD, one β-CD-modified wafer was immersed into 1 mL of a 1, 5, 10, 20 or 80 mM aqueous solution of Ada-ATRP, respectively,

for 45 min, ultrasonicated in this solution for 20 s and subsequently rinsed with water and dried under a flow of nitrogen and under vacuum.

SI-ARGET-ATRP. SI-ARGET-ATRP experiments were carried out using the following molar ratios monomer/copper(II)bromide/2,2′-bipyridine (bipy)/ascorbic acid = 1000 : 0.2 : 2.4 : 5.3 and with a monomer concentration of 2.56 M in water. First, the monomer (31.9 mmol, 1000 eq) and 12 mg bipy (76 µmol, 2.4 eq) were dissolved in 10.4 mL water. Simultaneously, in a separate vial, 7.15 mg copper(II)bromide (32 µmol) were dissolved in 10 mL water, and 2 mL of this solution was added to the solution containing the monomer. For a monomer concentration of 1.92 M, 1.28 M and 0.62 M, a monomer/copper(II)bromide ratio of 750 : 0.2, 500 : 0.2 and 250 : 0.2 was used. The resulting solution was degassed with two freeze-pump-thaw cycles (three in the case of HEMA) and finally, ascorbic acid (30 mg, 170 µmol, 5.3 eq) was added. Of this solution, 2 mL was then transferred into a nitrogen-purged vial containing one initiator-modified wafer, while still purging nitrogen through the vial and the reaction was allowed to run for up to 16 hrs at room temperature. If multiple wafers were used, each wafer was placed in a separate vial and 2 mL of polymerization solution was used per wafer. To stop the reaction, the wafers were rinsed with water and dried under a flow of nitrogen and under vacuum.

Patterning experiments. One drop (~30 µL) of a 20 mM aqueous solution of Ada-ATRP was drop-casted on one half of a flat lying β-CD-modified wafer using a Pasteur pipette. After 45 min, the drop was again removed with the Pasteur pipette and the wafer was extensively rinsed with water. Afterwards, the wafer was exposed to the previously described SI-ARGET-ATRP conditions using PEGMEMA as monomer. The polymerization was stopped after 16 hrs by rinsing the wafer with water and drying under a flow of nitrogen and under vacuum.

Evaluation of binding constants of surface-attached host-guest complexes. The Langmuir model describes the surface coverage as a function of concentration and association and dissociation rate constants k_{on} and k_{off} at equilibrium with equation (**1**). Insertion of the expression for the association constant K_A (**2**) into (**1**) gives the Langmuir isotherm (**3**) which was used to fit the data in **Figure 2B** to obtain K_A. [12]

$$k_{off}\theta = k_{on}c(1-\theta) \tag{1}$$

$$K_A = \frac{k_{on}}{k_{off}} \tag{2}$$

$$\theta = \frac{K_A c}{1 + K_A c} \tag{3}$$

2. SUPPORTING FIGURES

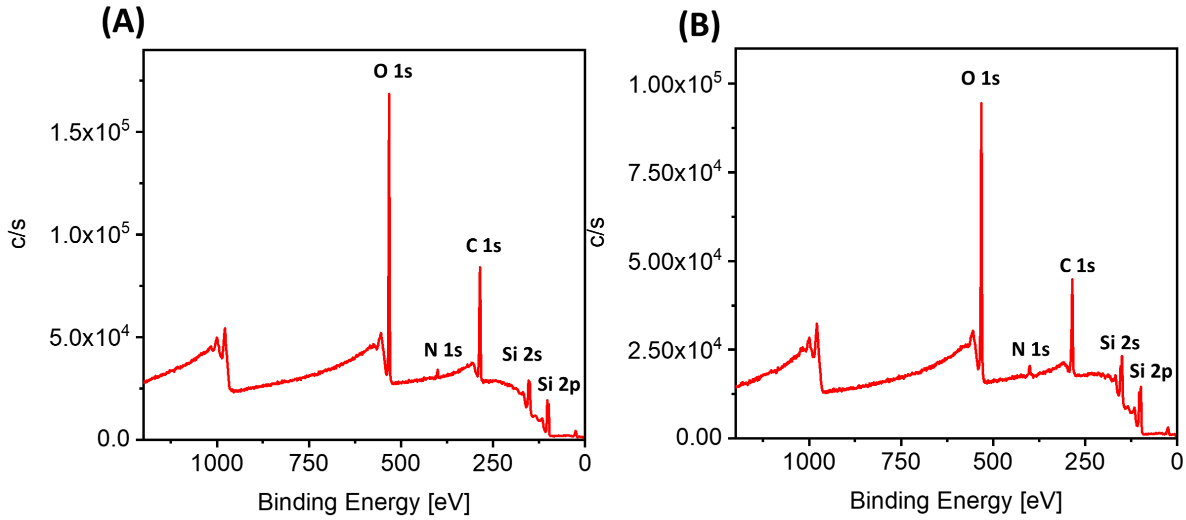

Figure S1. Survey XPS spectra of **(A)** a β-CD-modified silicon wafer, **(B)** an Ada-ATRP@ β-CD-modified silicon wafer.

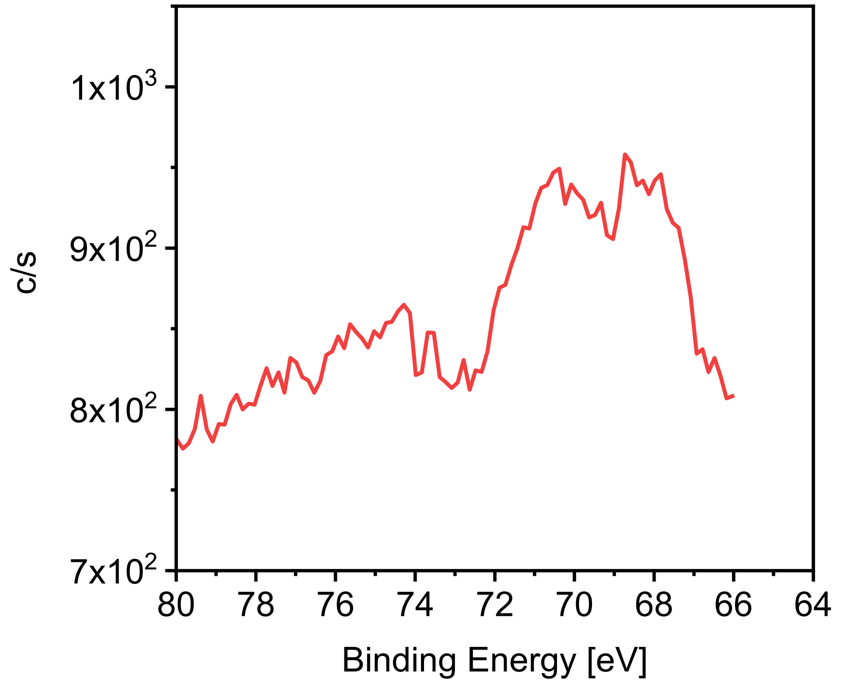

Figure S2. Br 3d XPS signal of a surface modified with CB[7]@Ada-ATRP.

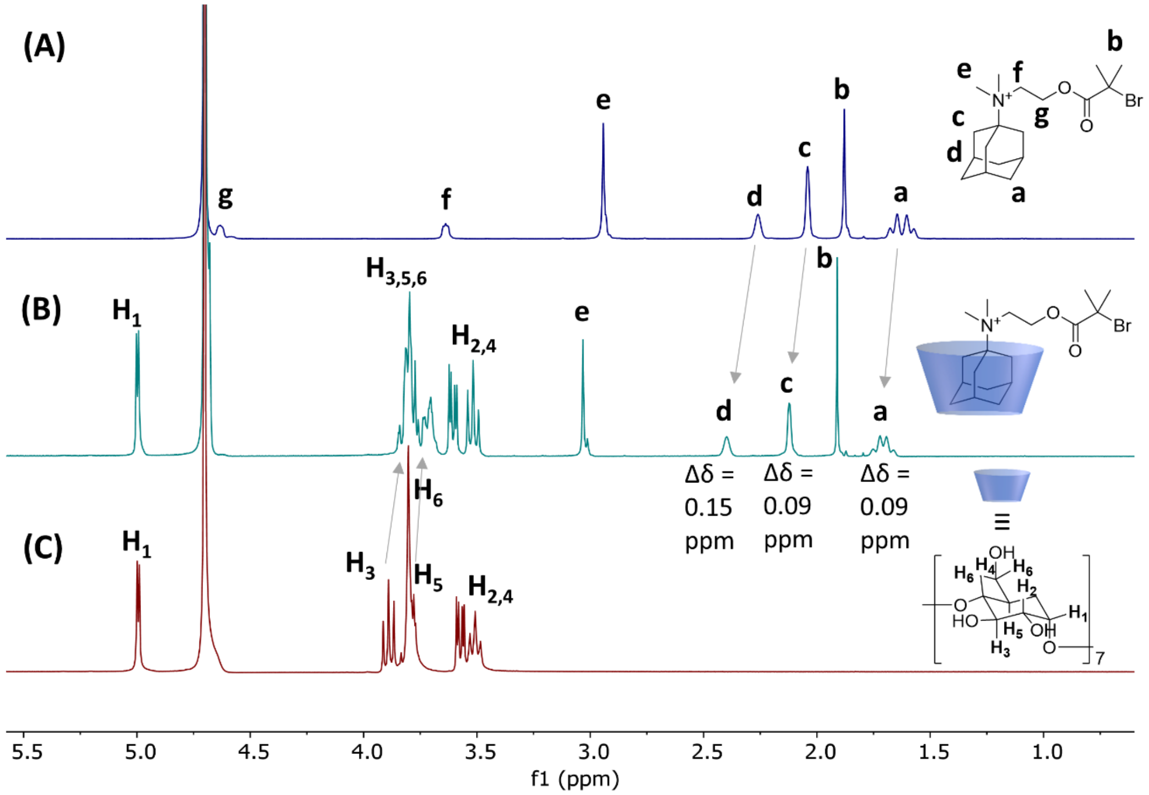

Figure S3. (A) ^{1}H NMR spectrum of Ada-ATRP in D_2O; **(B)** ^{1}H-NMR spectrum of Ada-ATRP (1 mM) with 1.5 eq of β-CD in D_2O and **(C)** ^{1}H-NMR spectrum of β-CD in D_2O.

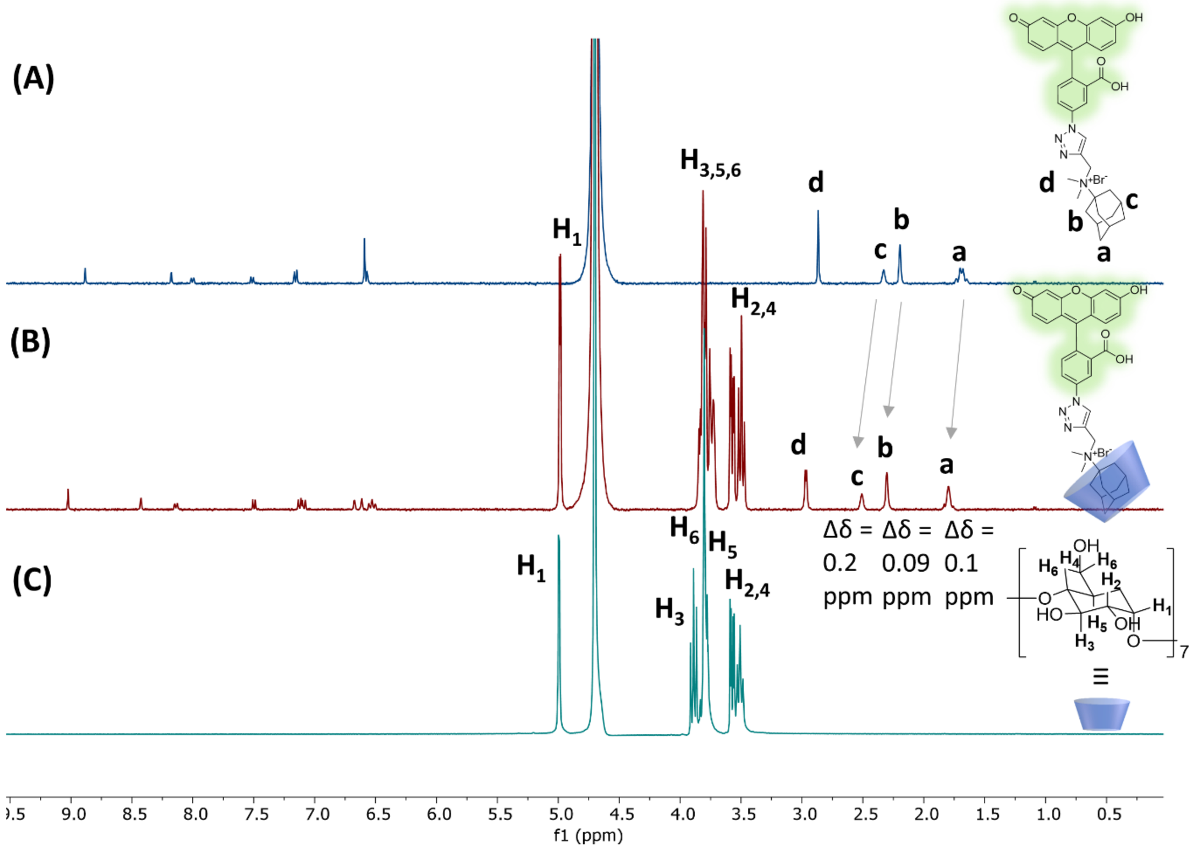

Figure S4. (A) ^{1}H NMR spectrum of Ada-Flu in D_2O; **(B)** ^{1}H-NMR spectrum of Ada-Flu (1 mM) with 1.5 eq of β-CD in D_2O, and **(C)** ^{1}H-NMR spectrum of β-CD in D_2O.

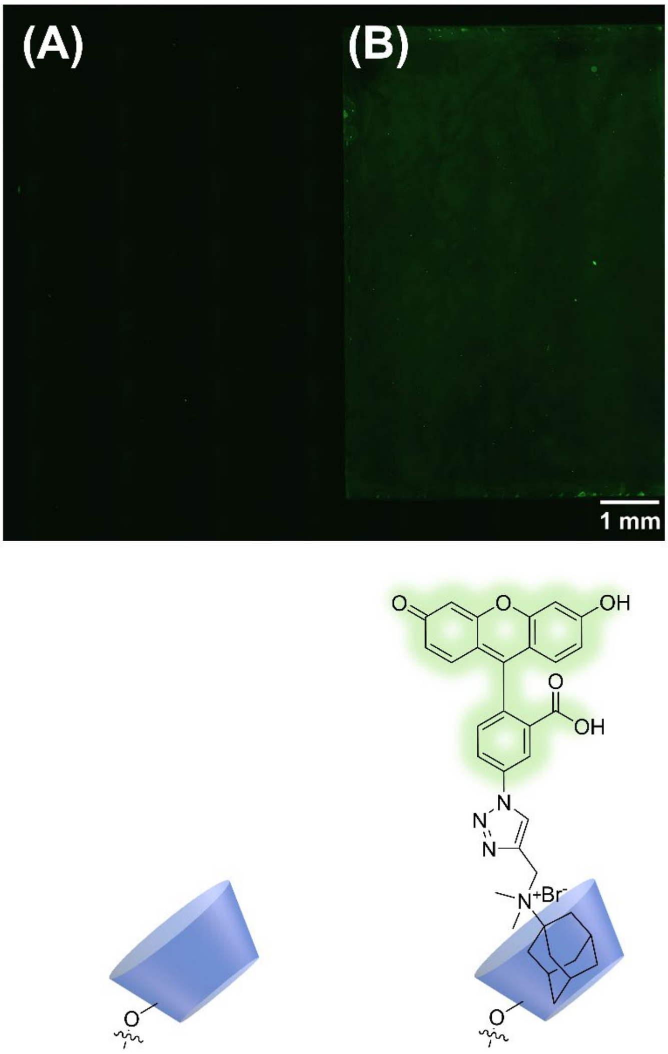

Figure S5. Fluorescence microscopy images of (**A**) a fused silica wafer modified with β-CD; (**B**) a fused silica wafer modified with β-CD@Ada-Flu.

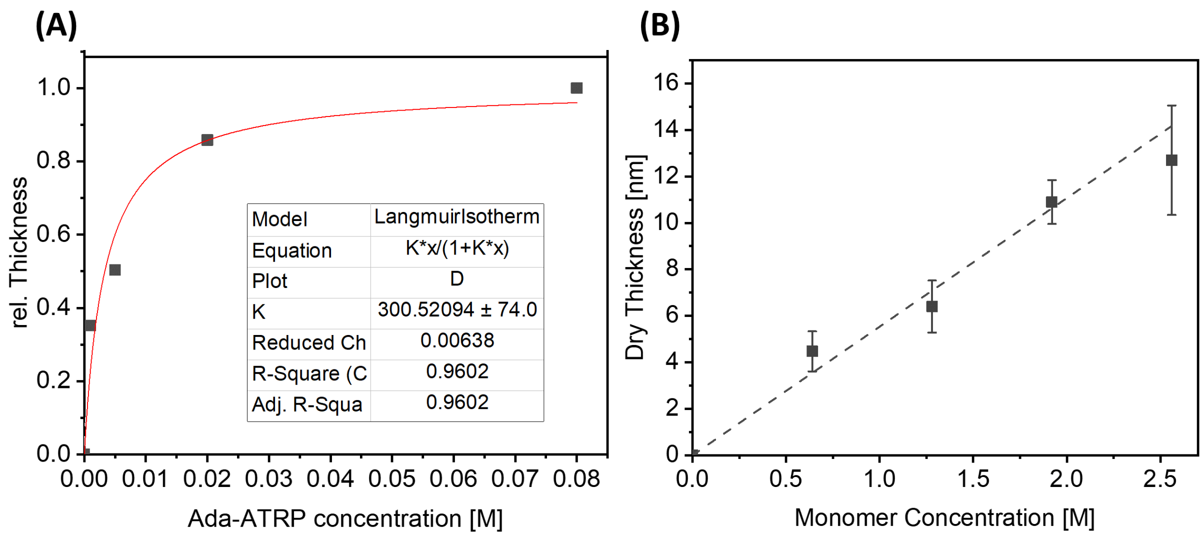

Figure S6. (A) Relative film thickness of PMPC brushes grown from β-CD-modified silicon substrates at a monomer concentration of 2.56 M and after a polymerization time of 4 h, as a function of the Ada-ATRP concentration that was used to form the surface-bound non-covalent ATRP initiator modified host-guest complex; **(B)** Dry film thicknesses of PMPC brushes after 4 hrs of polymerization time as a function of monomer (MPC) concentration grafted from CD-functionalized surfaces that were treated with a 20 mM Ada-ATRP solution to form the surface-bound initiator.

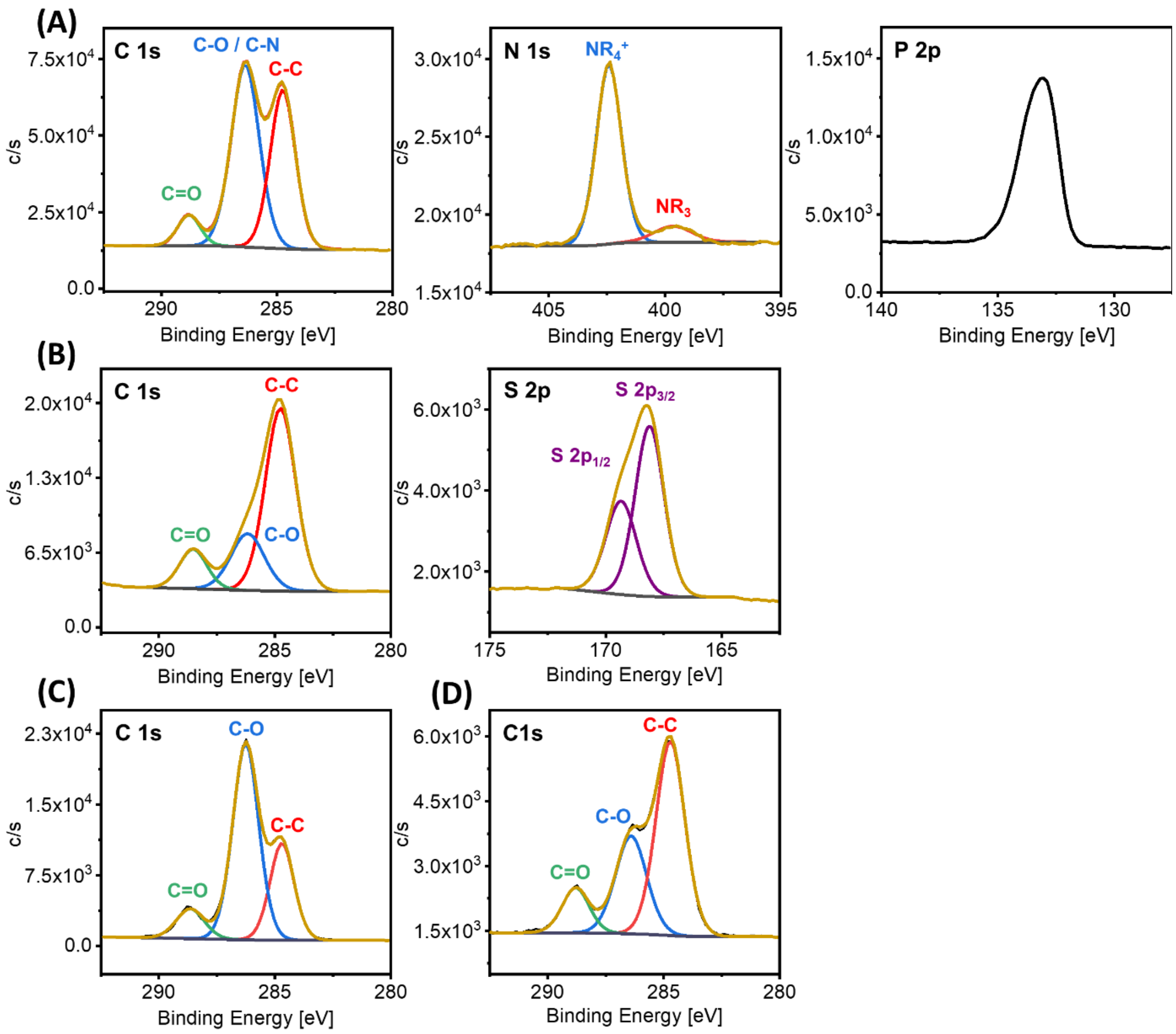

Figure S7. XPS high resolution scans of **(A)** the C 1s, N 1s and P 2s regions of a 15 nm thick PMPC brush, **(B)** the C 1s and S 2p regions of a 12 nm thick PSPMA brush, **(C)** the C 1s region of a 40 nm thick PPEGMEMA brush, and **(D)** the C 1s region of a 10 nm thick PHEMA brush.

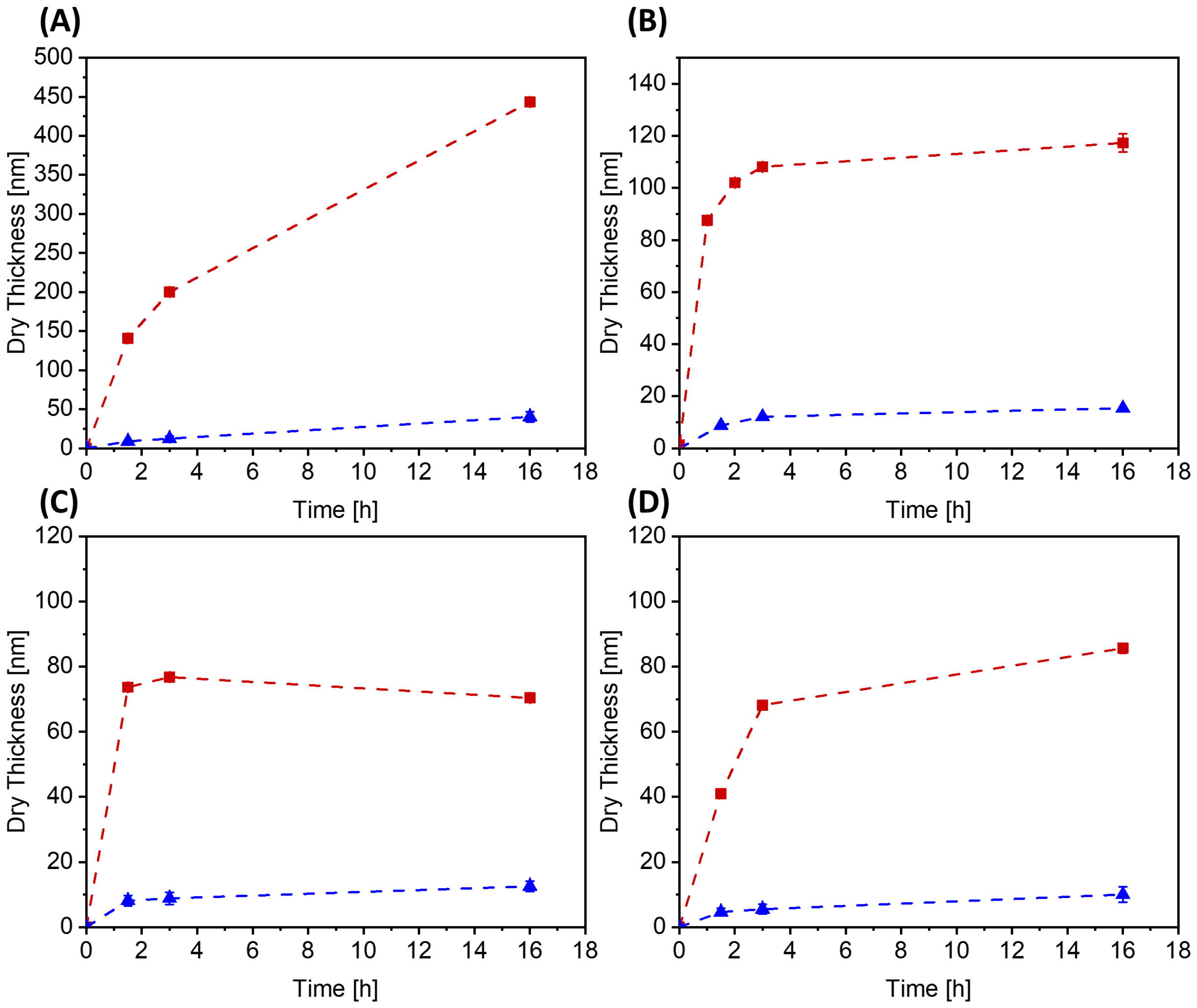

Figure S8. Growth profiles of CB[7]- (■) and β-CD (▲) tethered **(A)** PPEGMEMA, **(B)** PMPC, **(C)** PSPMA and **(D)** PHEMA brushes.

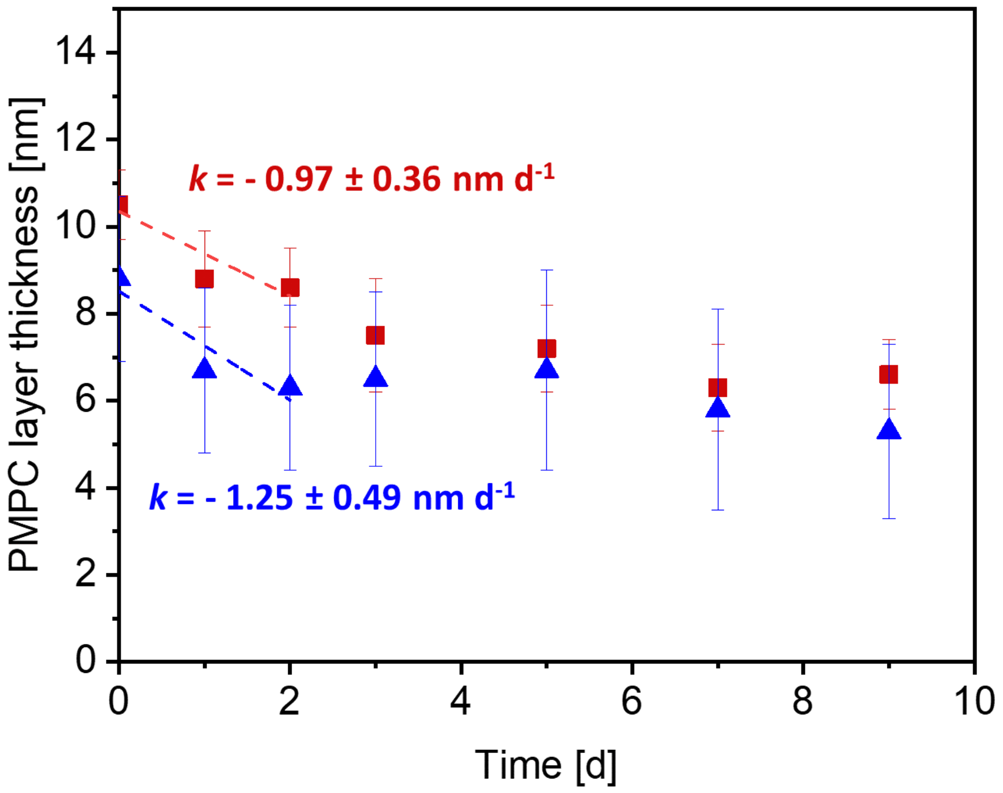

Figure S9. Stability of CB[7] (■)- and β-CD(▲)-tethered supramolecular polymer brushes in MilliQ water. Polymer brushes were incubated in 10 L MilliQ water that was replaced daily.

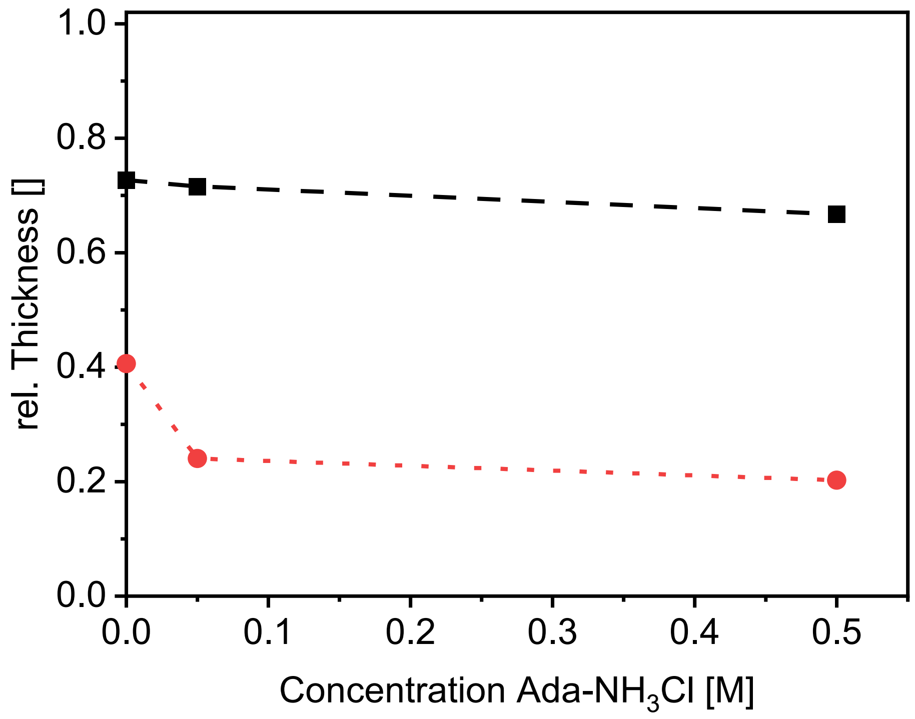

Figure S10. Relative thickness of CB[7]-tethered supramolecular polymer brushes (PMPC) as a function of the concentration of ammonium adamantane (Ada-NH_3Cl) in PBS, after 24 hrs of incubation at room temperature (■) or at 60°C (●).

3. NMR- AND MASS SPECTRA

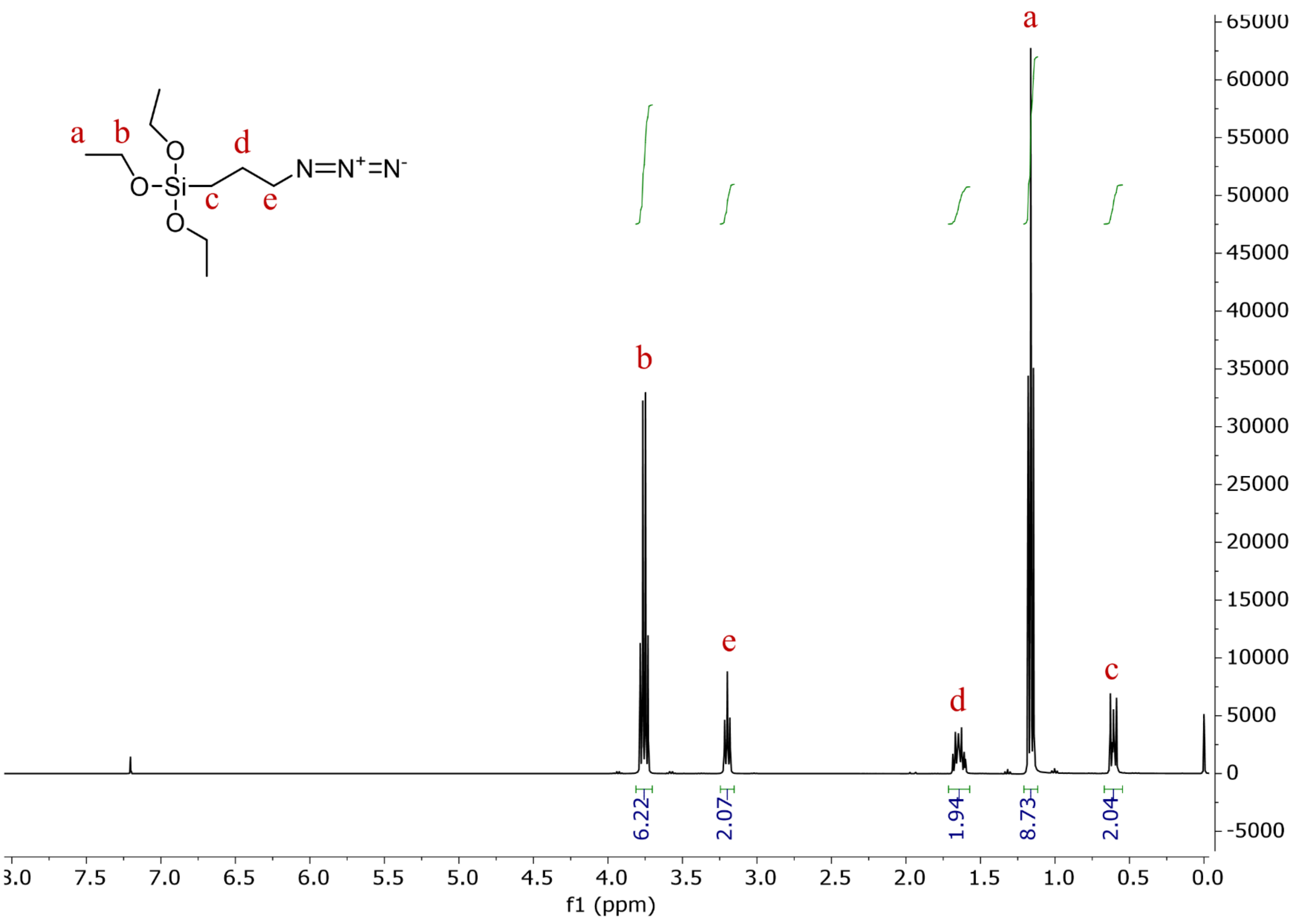

Figure S11. ^{1}H NMR spectrum of (3-azidopropyl)triethoxysilane ($CDCl_3$).

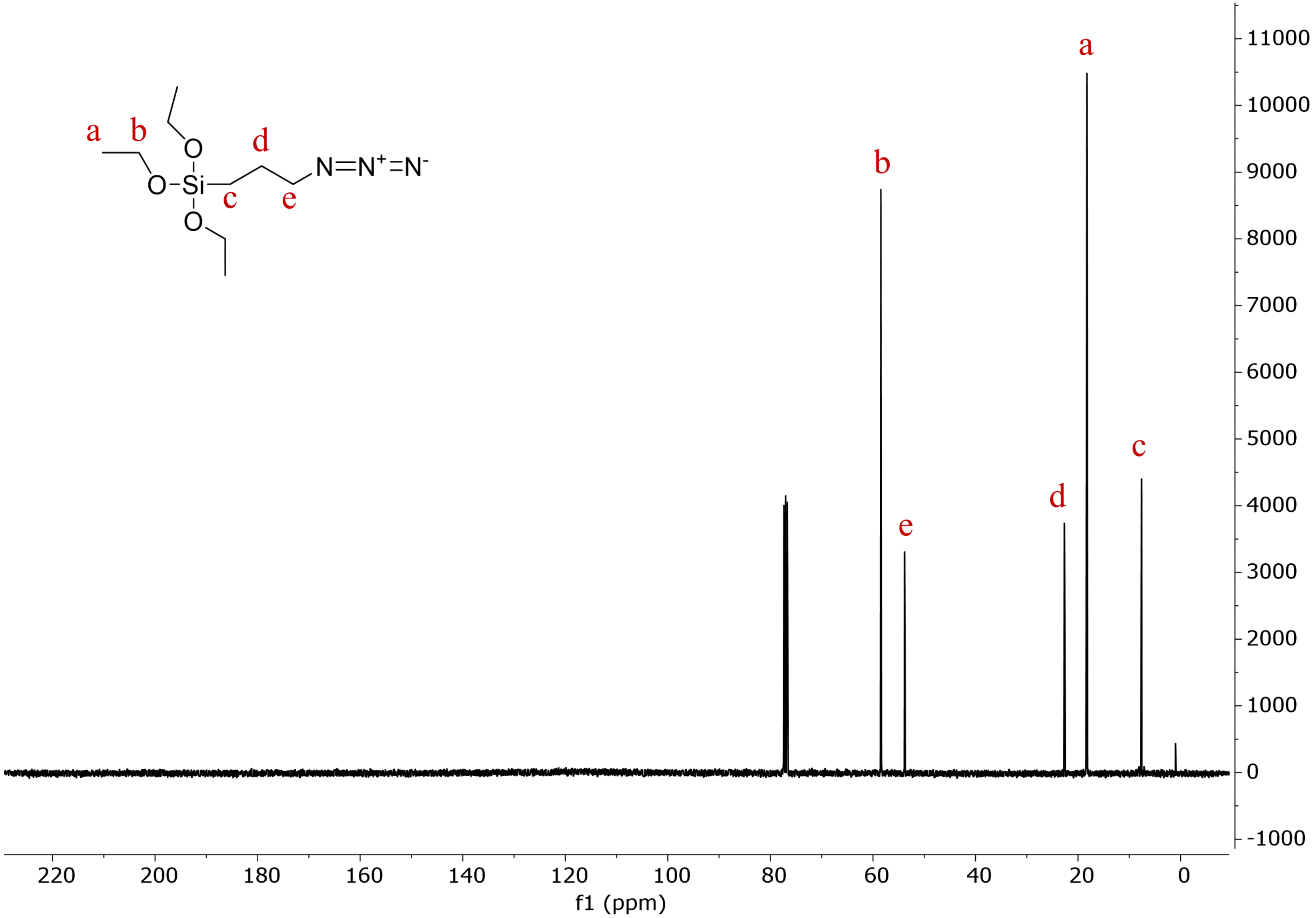

Figure S12. ^{13}C NMR spectrum of (3-azidopropyl)triethoxysilane ($CDCl_3$).

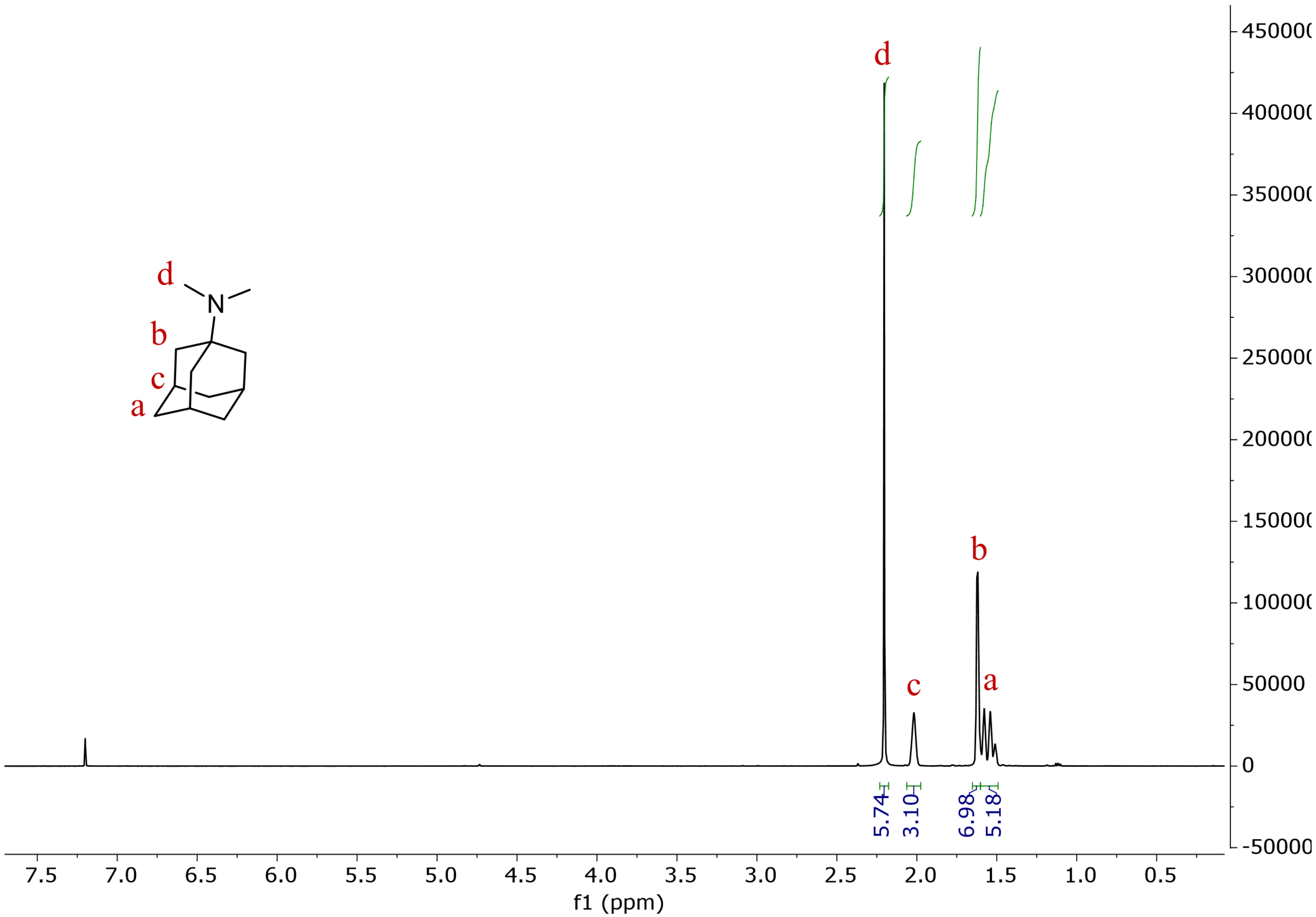

Figure S13. ^{1}H NMR spectrum of *N,N*-dimethyladamantan-1-amine ($CDCl_3$).

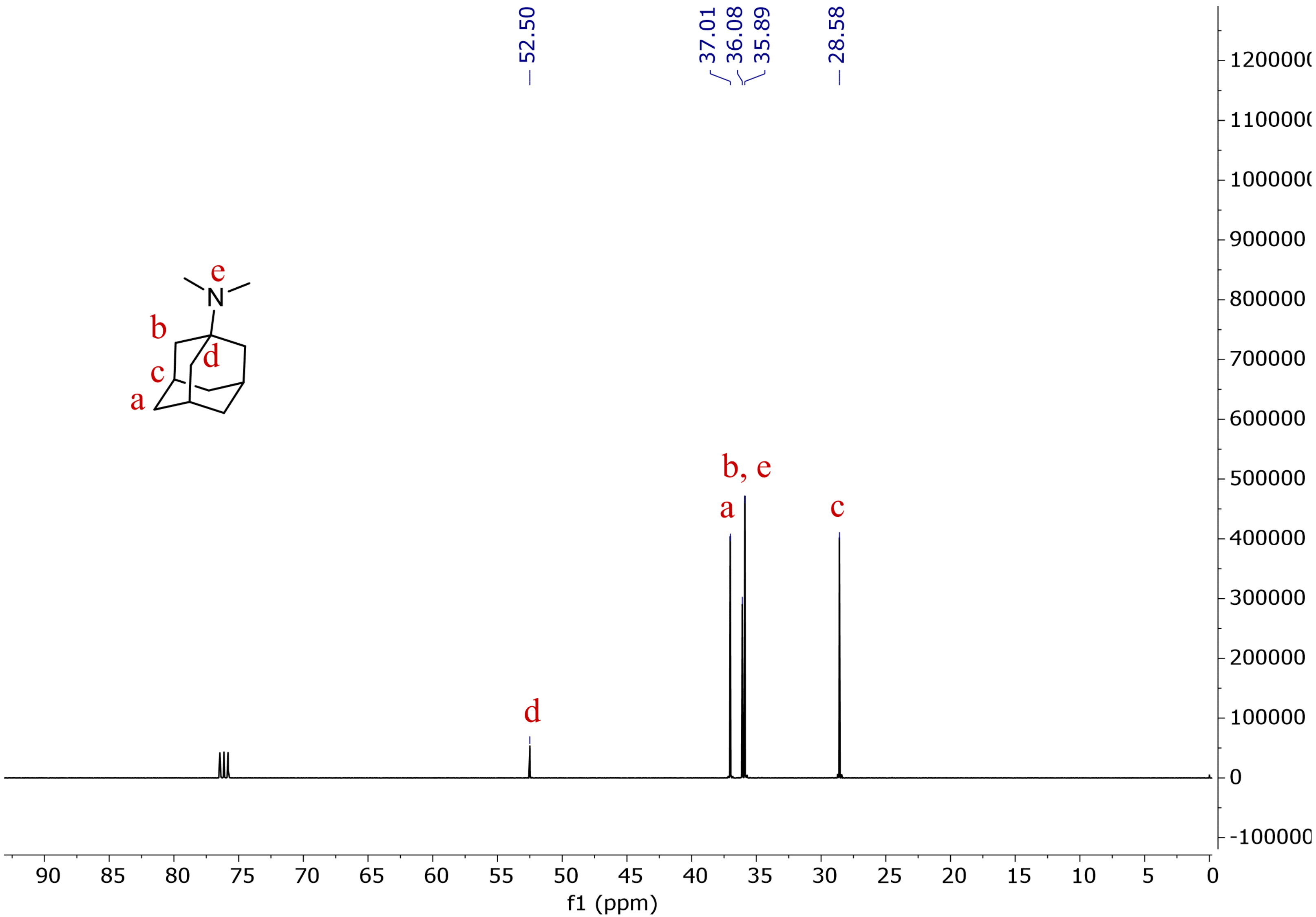

Figure S14. ^{13}C NMR spectrum of *N,N*-dimethyladamantan-1-amine ($CDCl_3$).

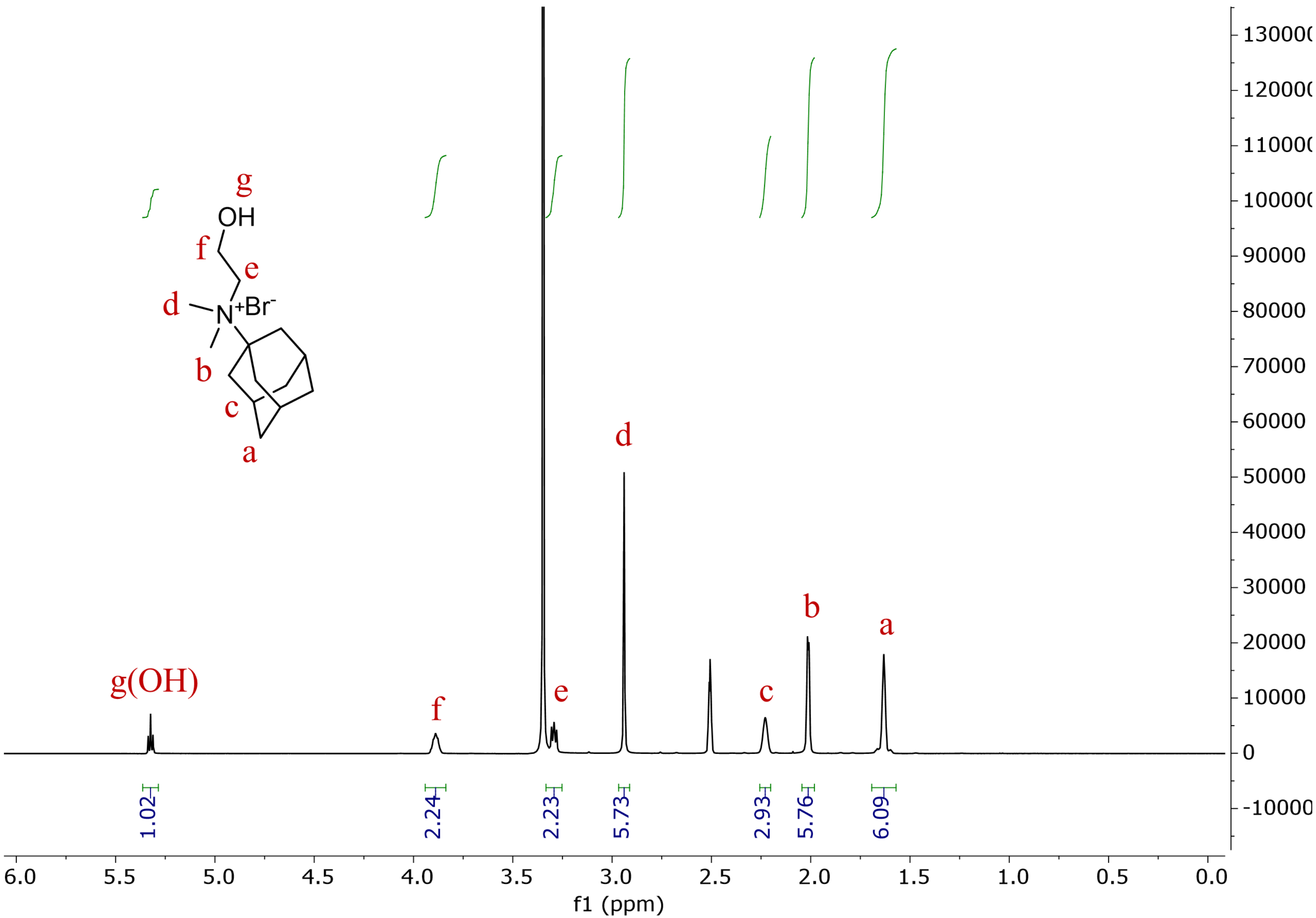

Figure S15. ^{1}H NMR spectrum of *N*-(2-hydroxyethyl)-*N,N*-dimethyladamantan-1-aminium (Ada-OH) (DMSO-*d6*).

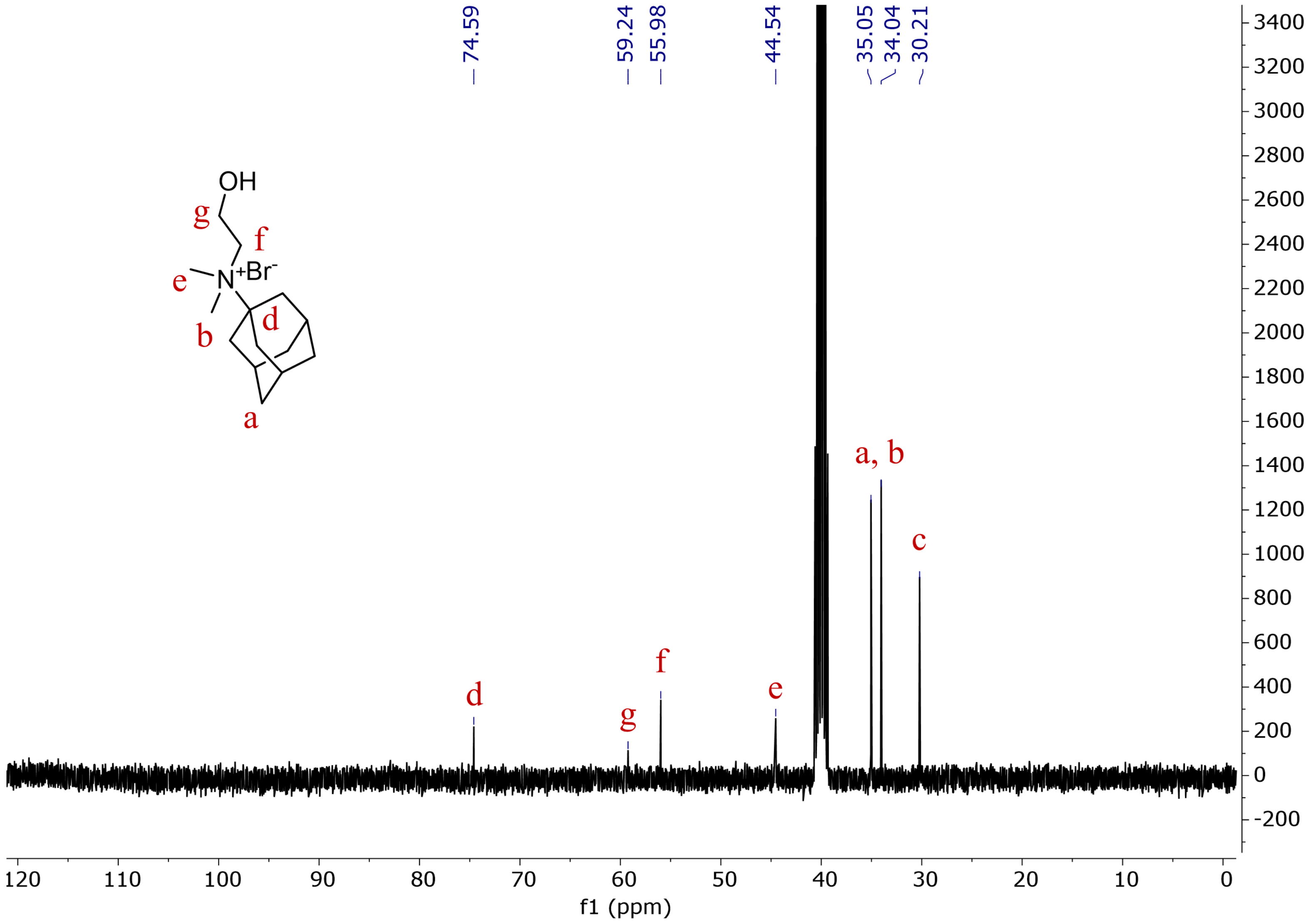

Figure S16. ^{13}C NMR spectrum of *N*-(2-hydroxyethyl)-*N,N*-dimethyladamantan-1-aminium (Ada-OH) (DMSO-*d6*).

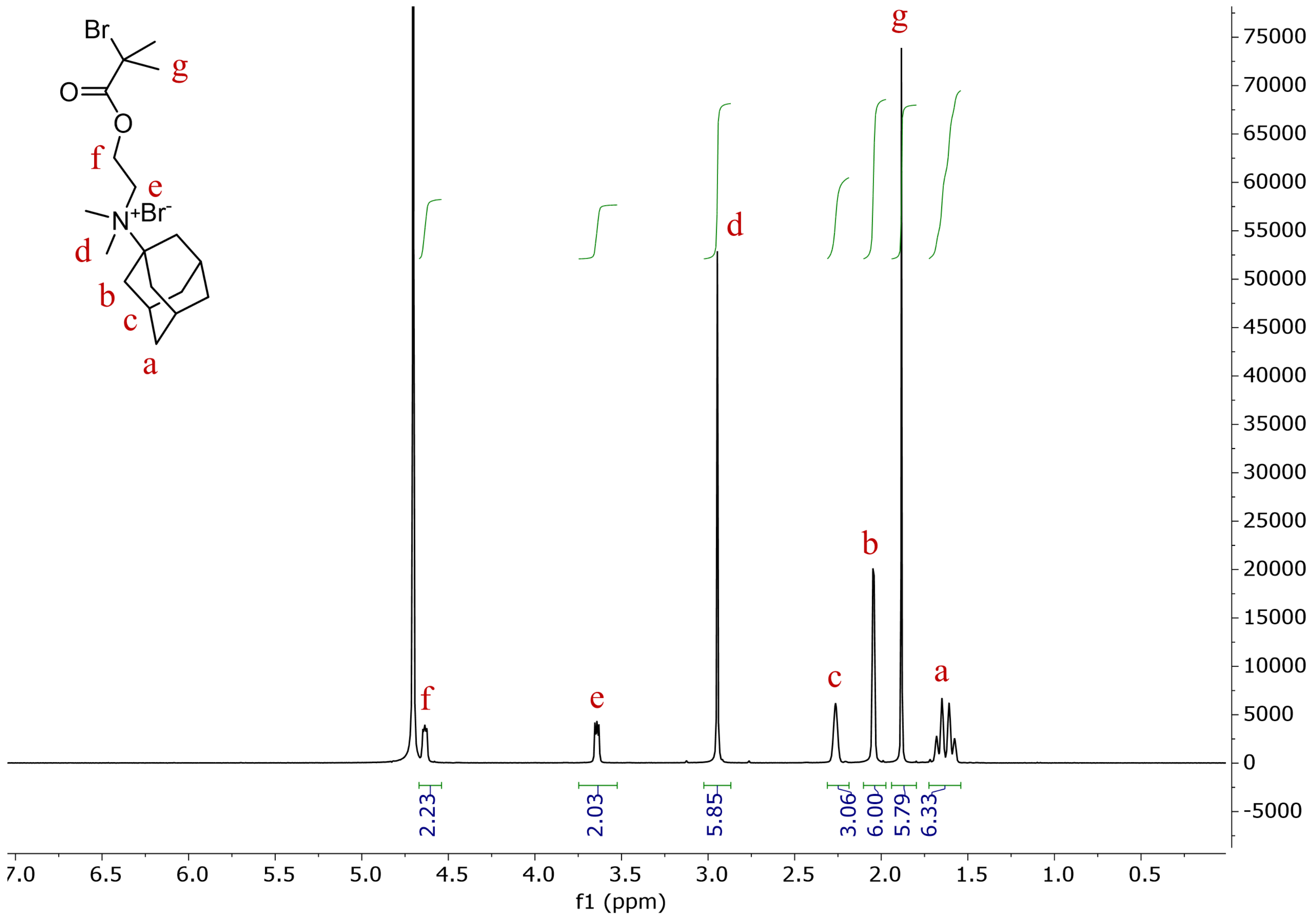

Figure S17. ^{1}H NMR spectrum of *N*-(2-((2-bromo-2-methylpropanoyl)oxy)ethyl)-*N,N*-dimethyladamantan-1-aminium (Ada-ATRP) (D_2O).

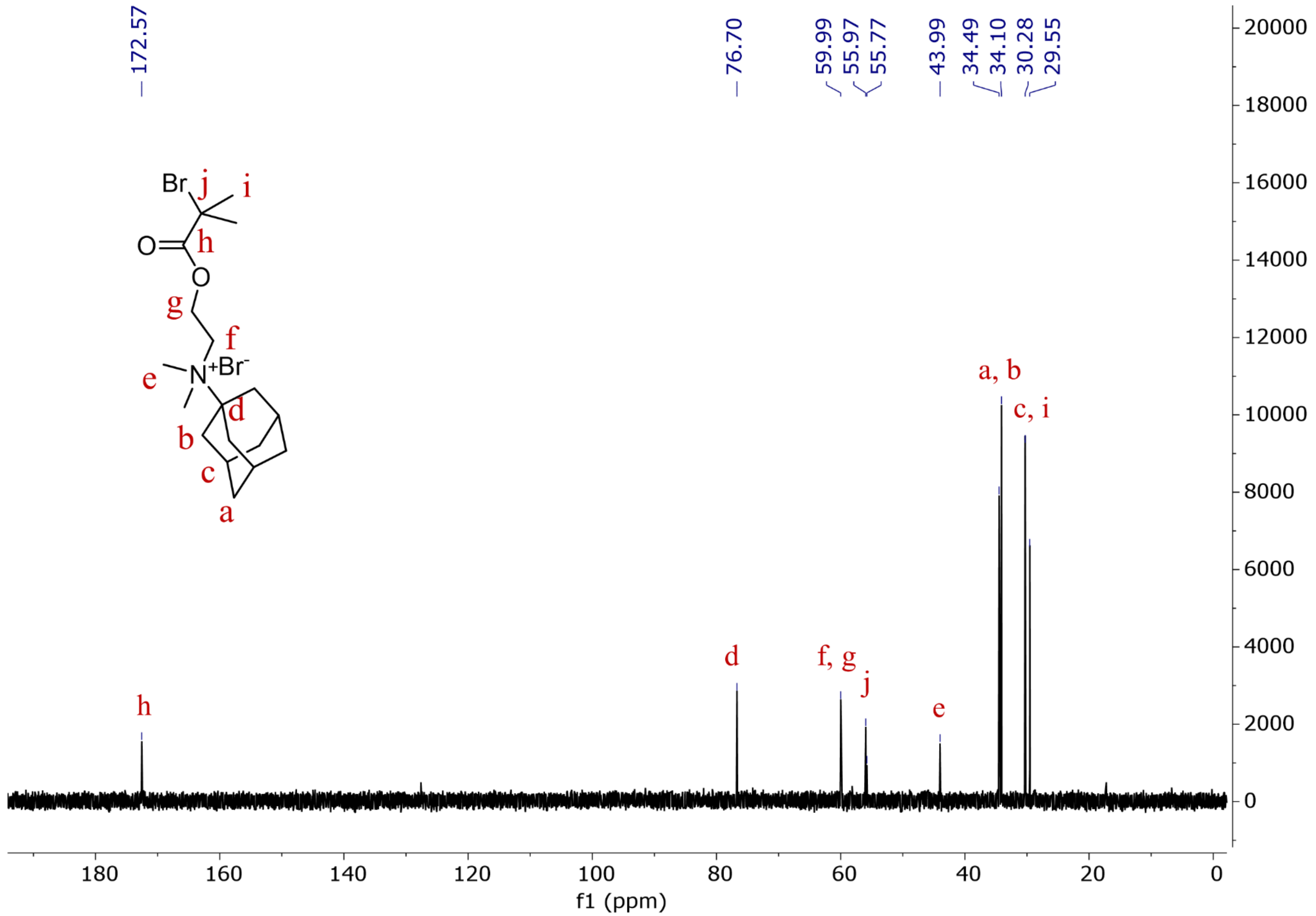

Figure S18. ^{13}C NMR spectrum of *N*-(2-((2-bromo-2-methylpropanoyl)oxy)ethyl)-*N,N*-dimethyladamantan-1-aminium (Ada-ATRP) (D_2O).

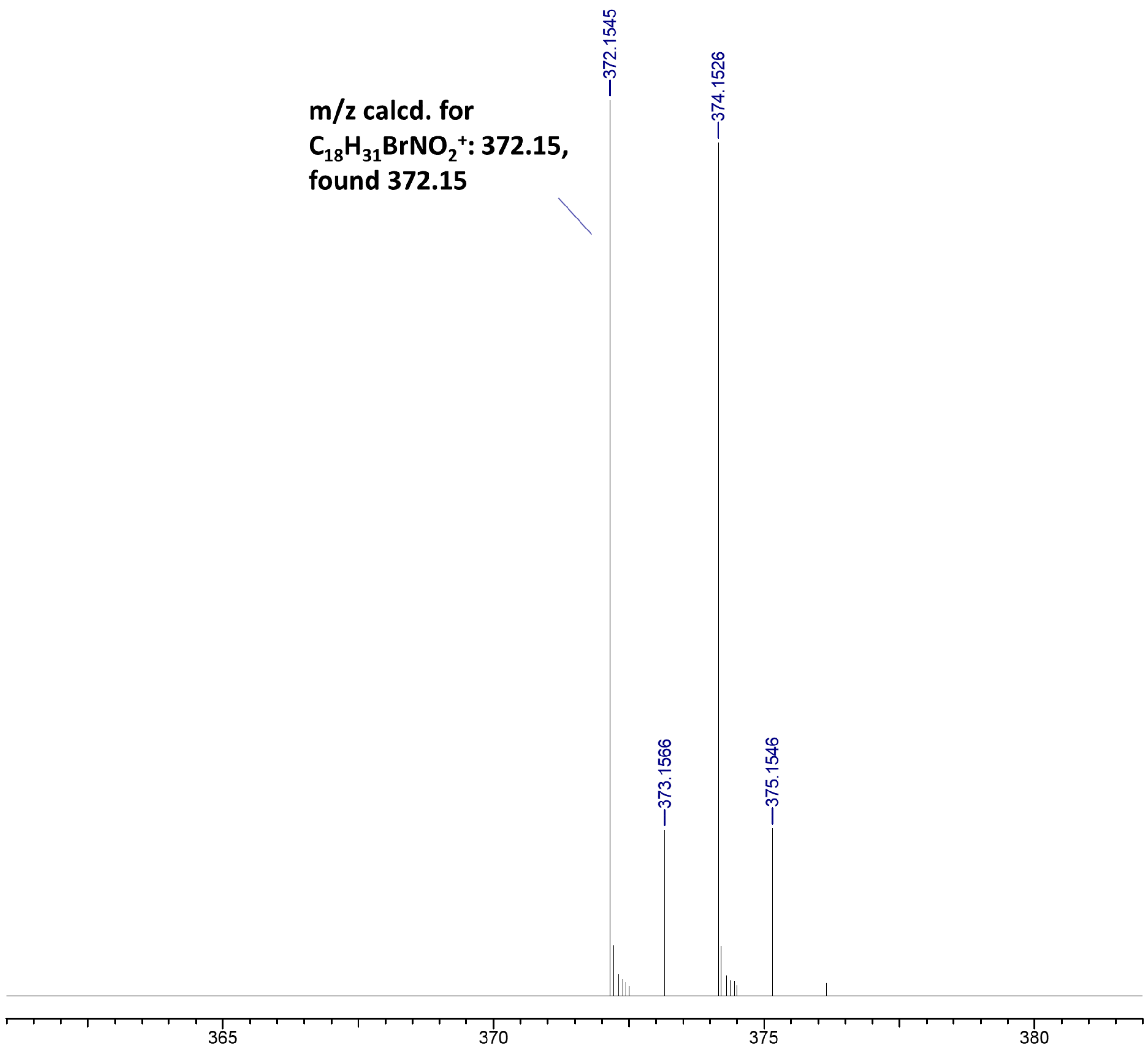

Figure S19. HR-MS (ES+) spectrum of Ada-ATRP.

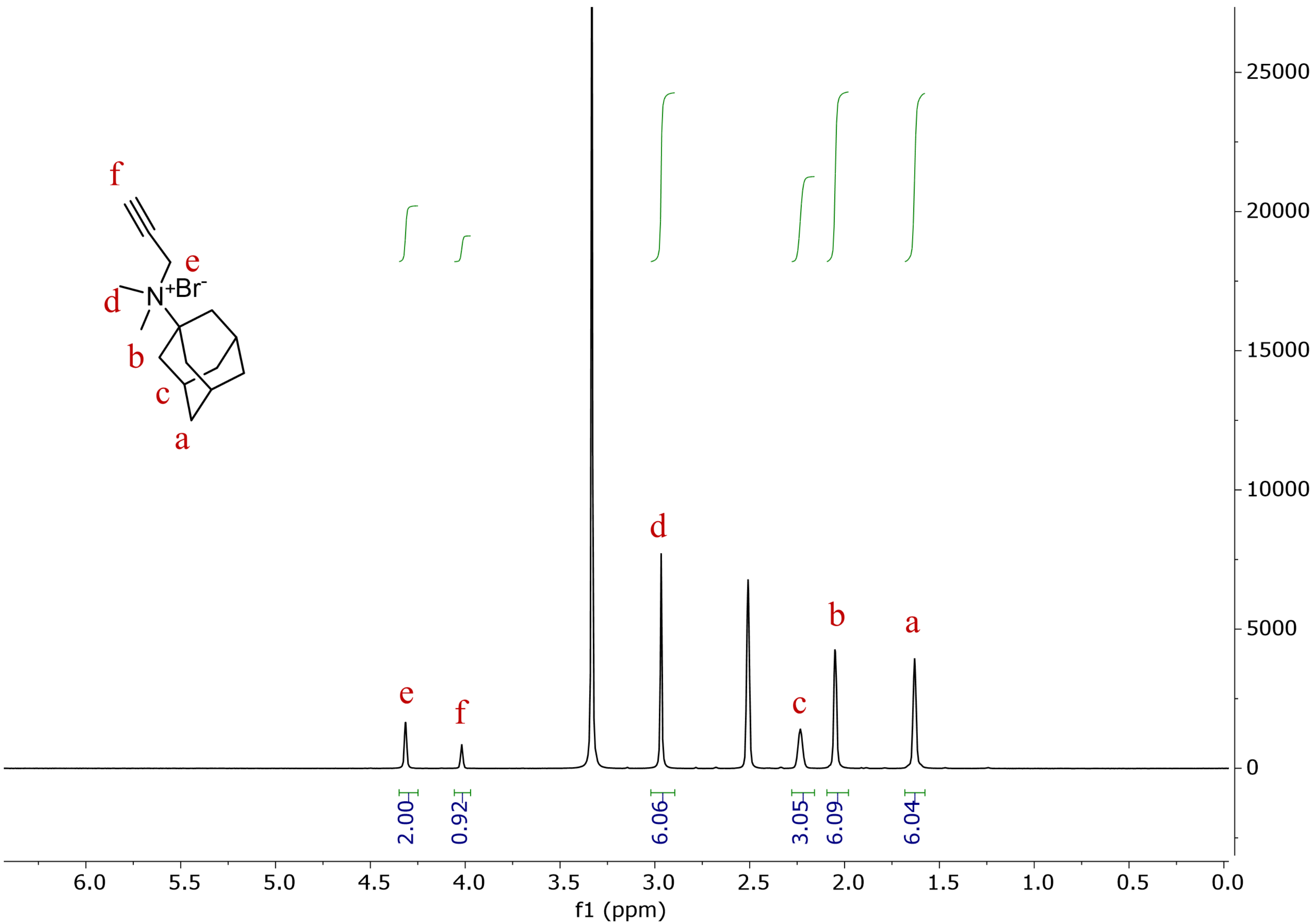

Figure S20. ^{1}H NMR spectrum of *N,N*-dimethyl-*N*-(prop-2-yn-1-yl)adamantan-1-aminium (DMSO-*d*6).

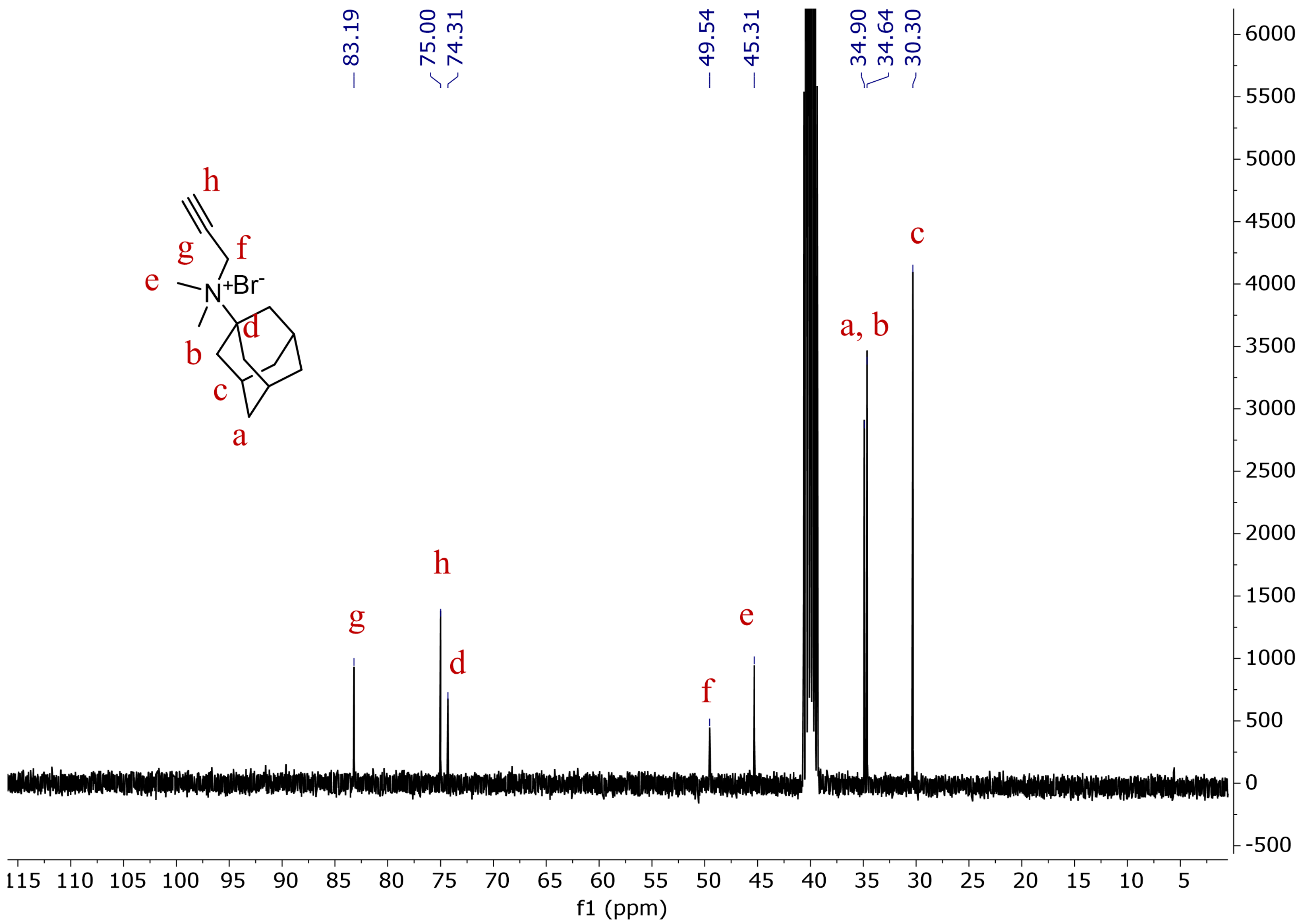

Figure S21. ^{13}C NMR spectrum of *N,N*-dimethyl-*N*-(prop-2-yn-1-yl)adamantan-1-aminium (DMSO-*d*6).

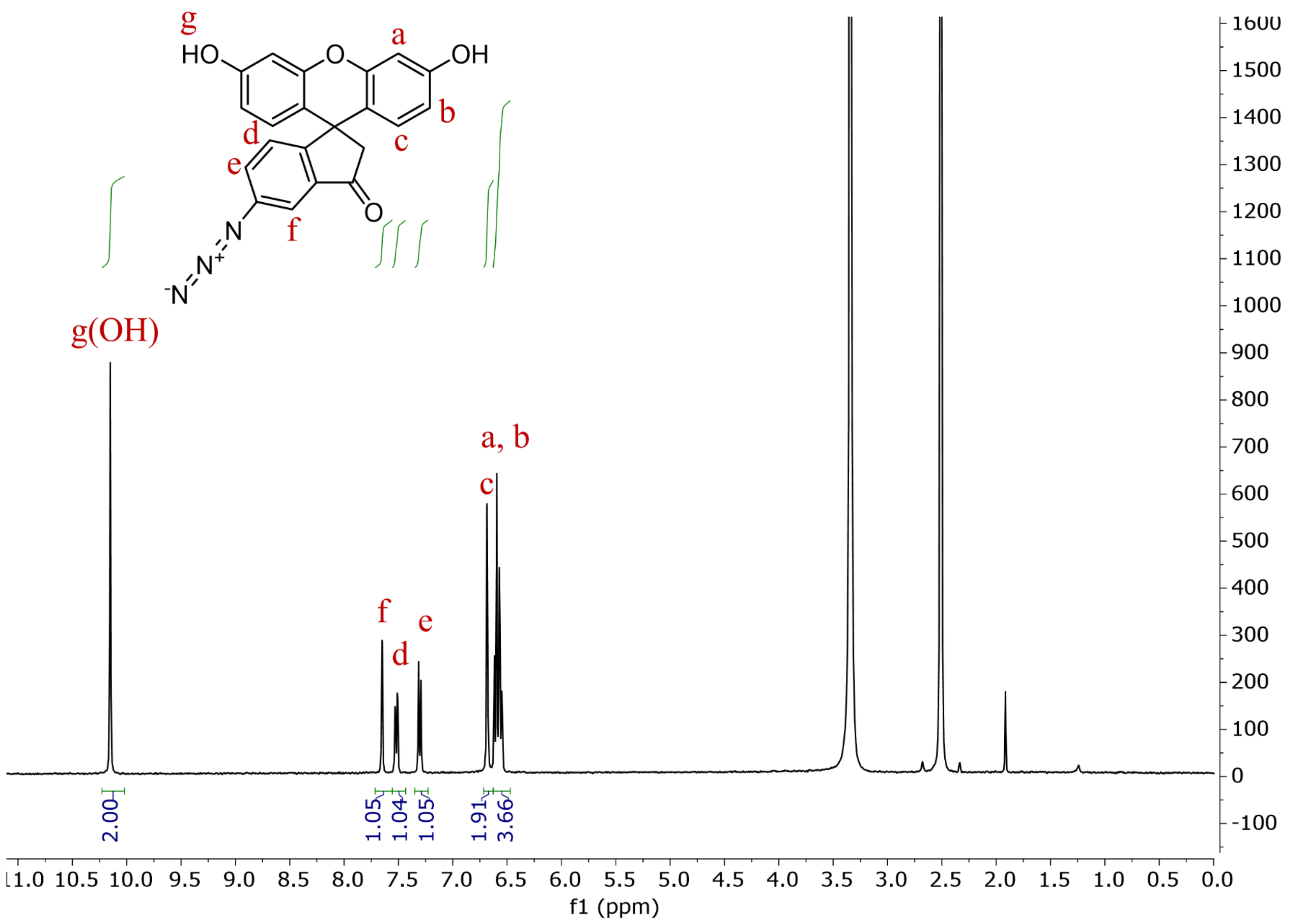

Figure S22. ^{1}H NMR spectrum of 5-azidofluorescein (DMSO-*d*6).

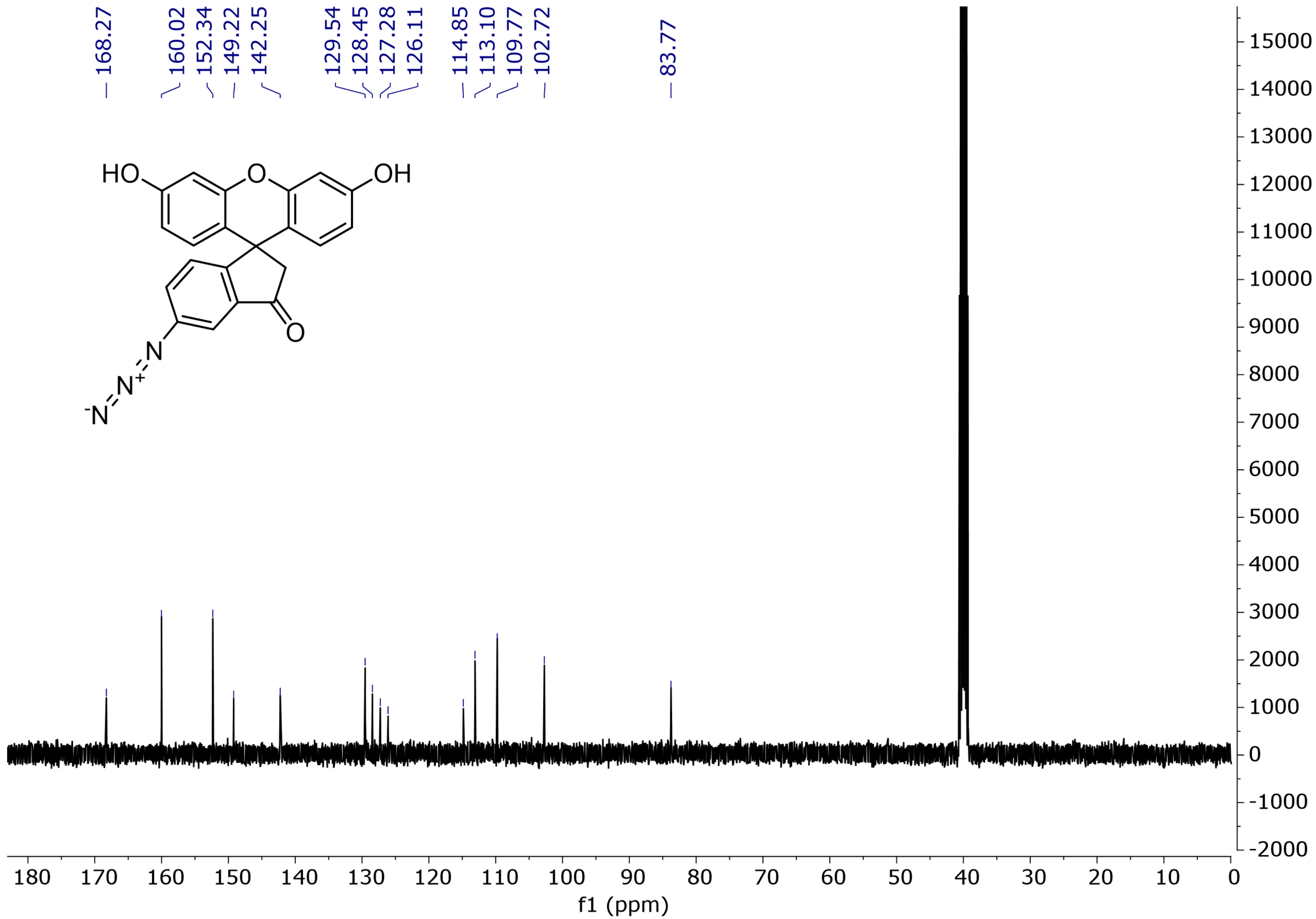

Figure S23. ^{13}C NMR spectrum of 5-azidofluorescein (DMSO-*d6*).

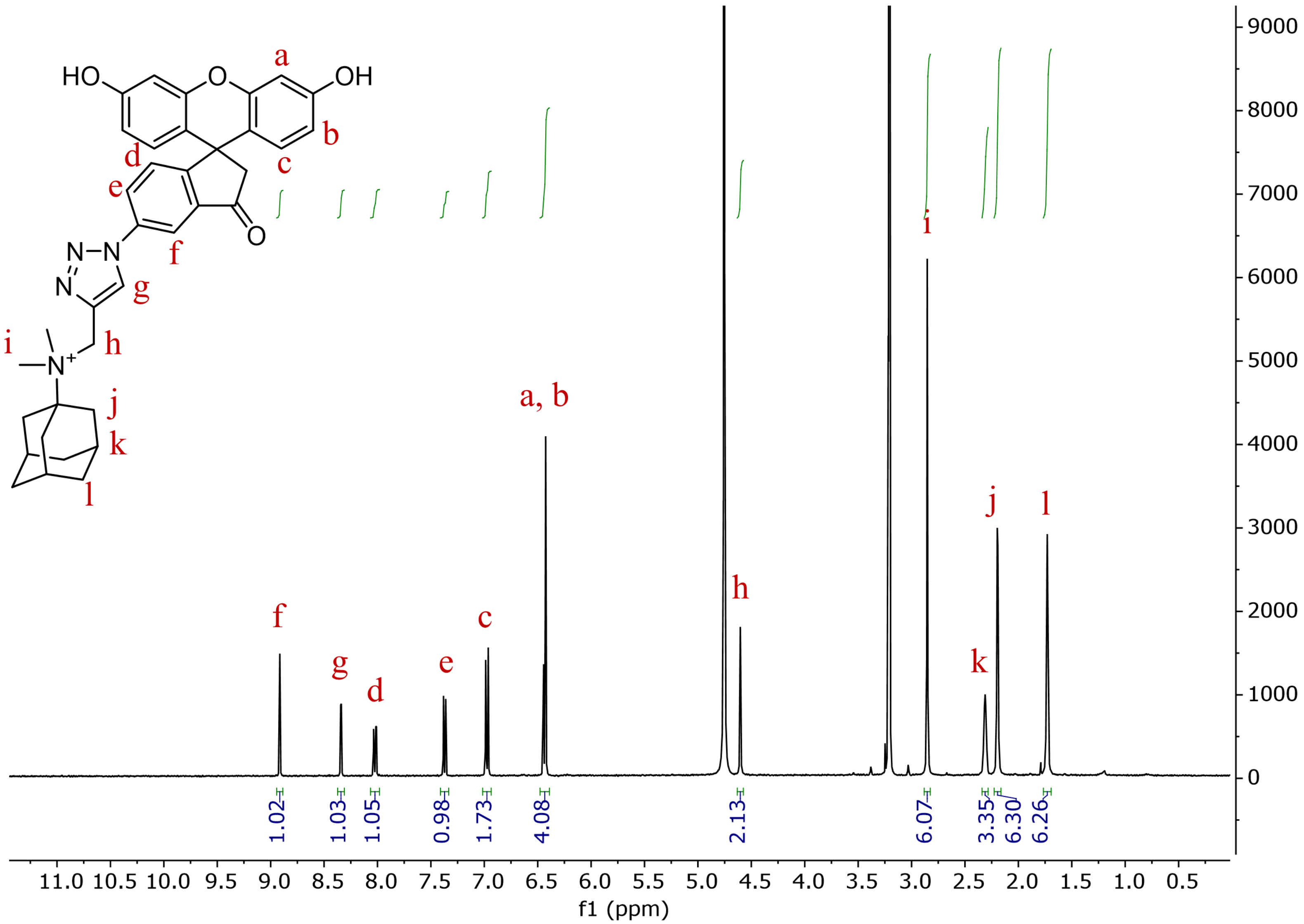

Figure S24. ^{1}H NMR spectrum of Ada-Flu (MeOD-*d*4).

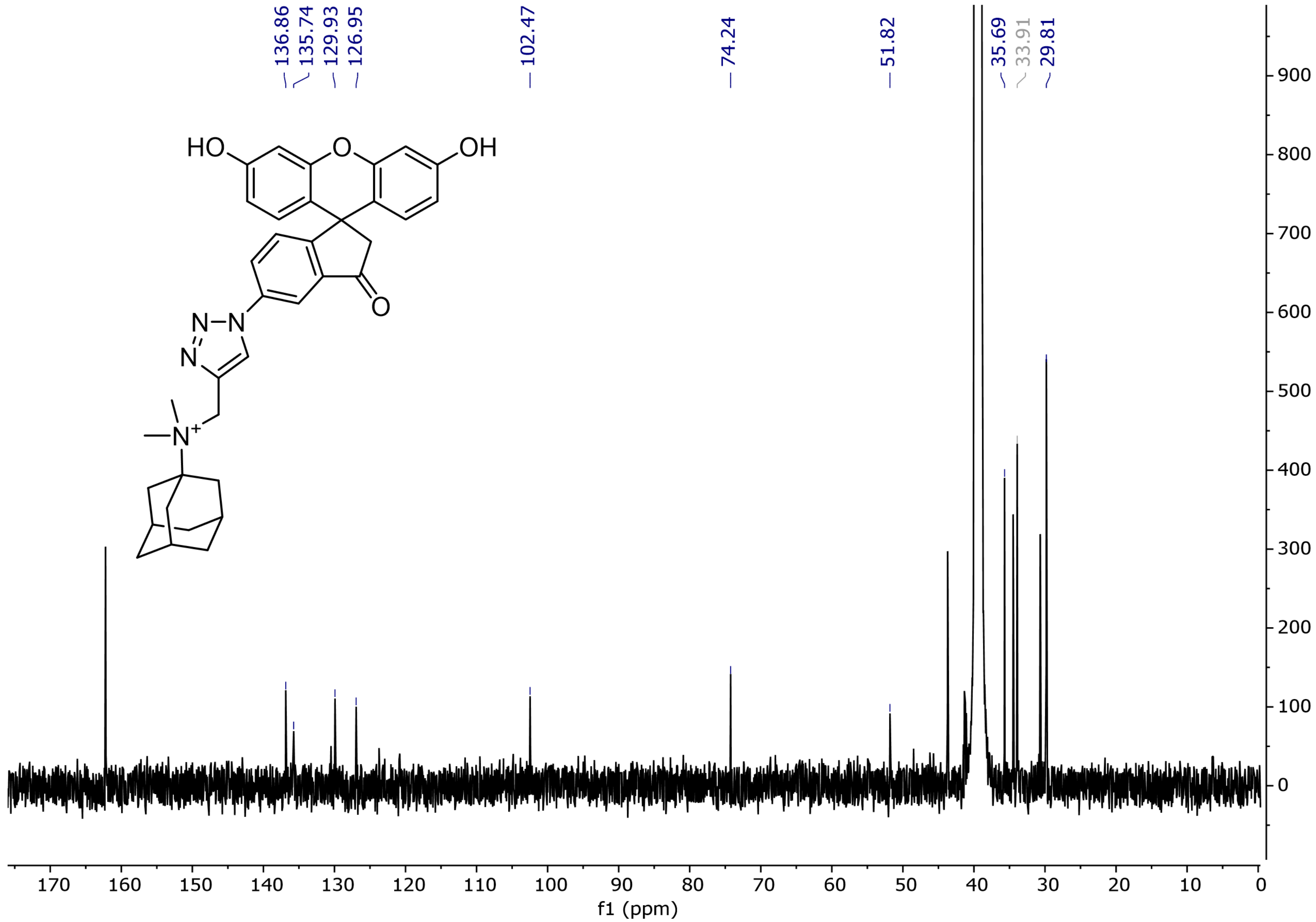

Figure S25. ^{13}C NMR spectrum of Ada-Flu (MeOD-*d*4).

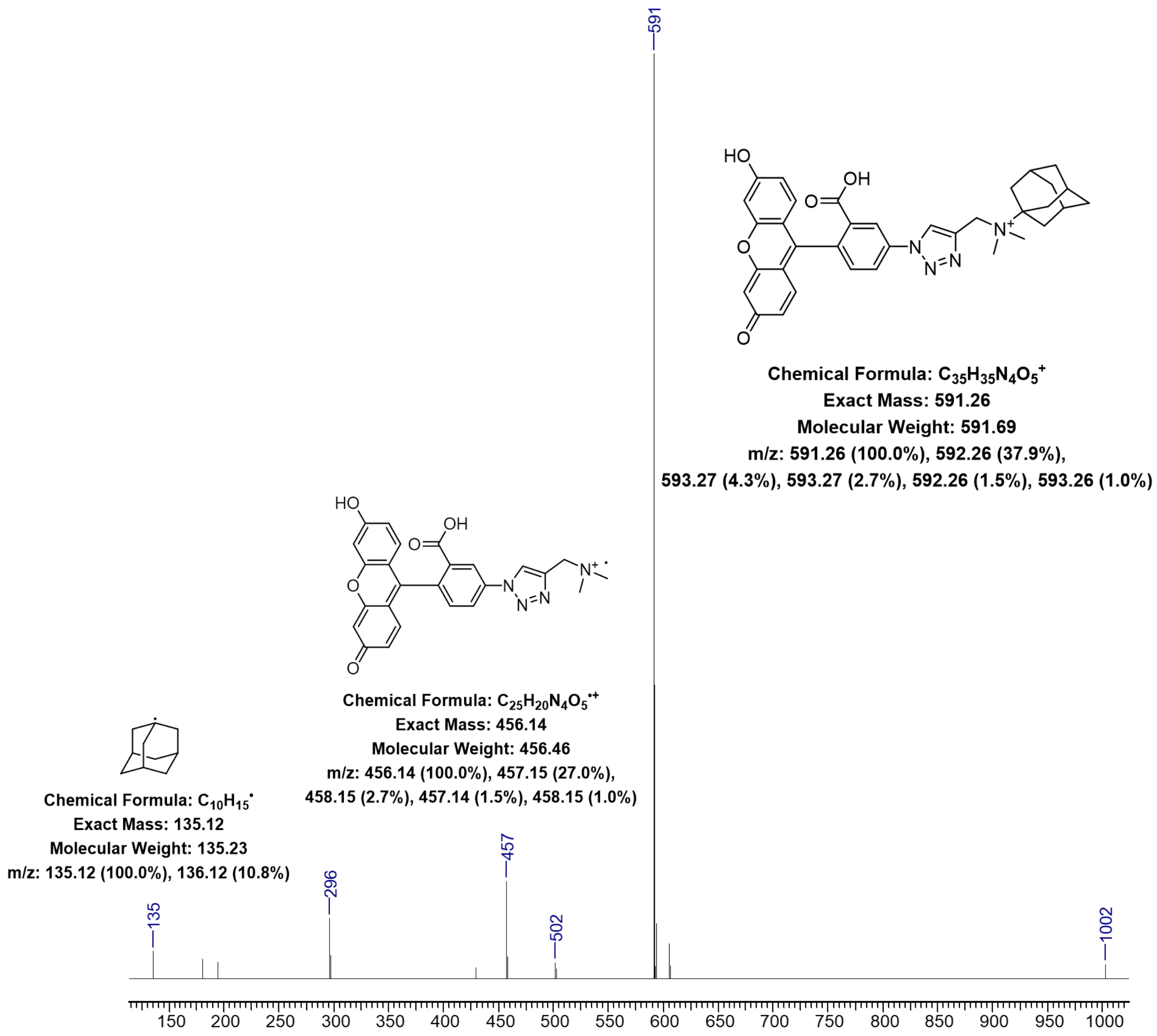

Figure S26. HR-MS (ES+) spectrum of Ada-Flu.

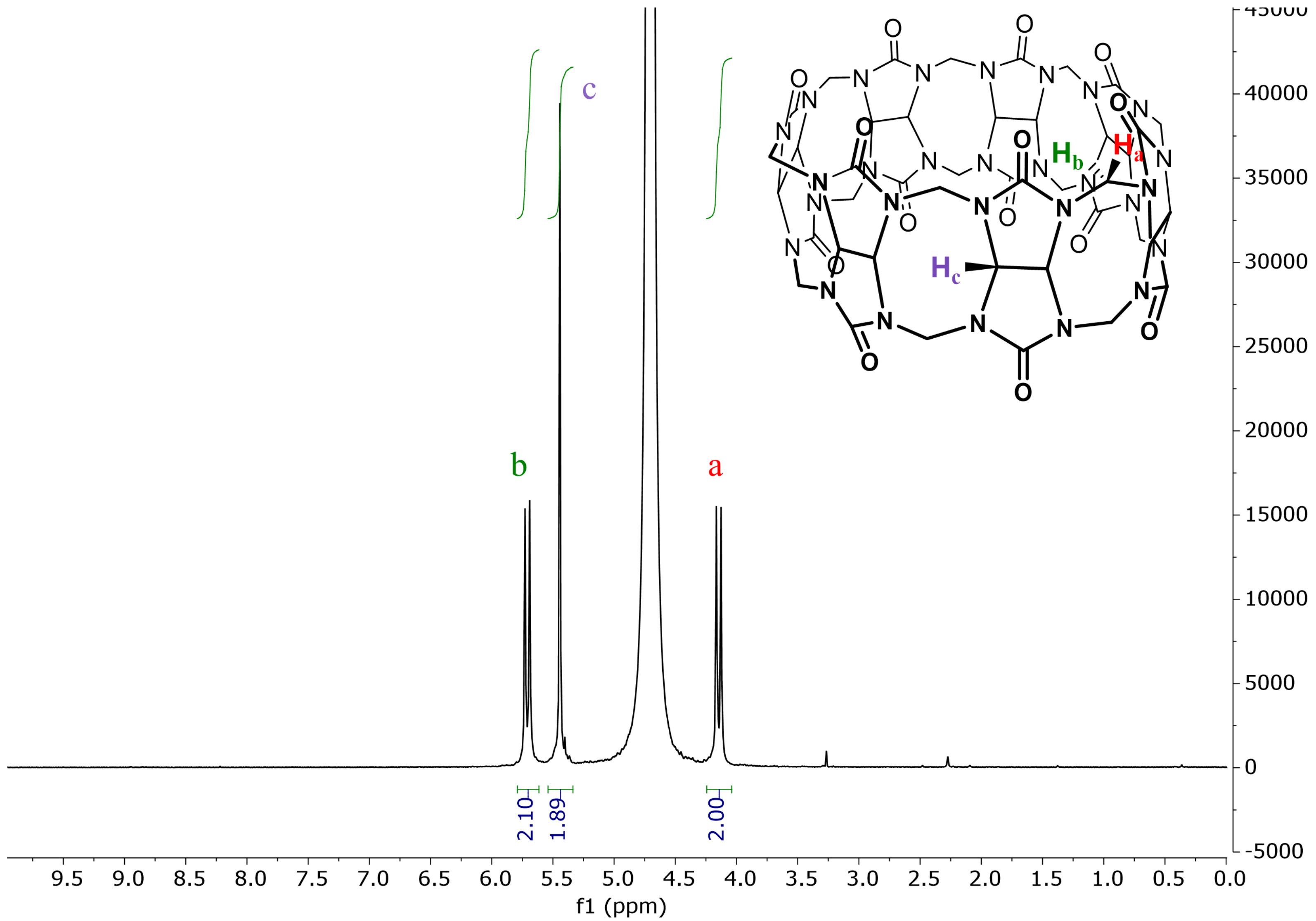

Figure S27. ^{1}H NMR spectrum of cucurbit[7]uril (CB[7]) (D_2O).

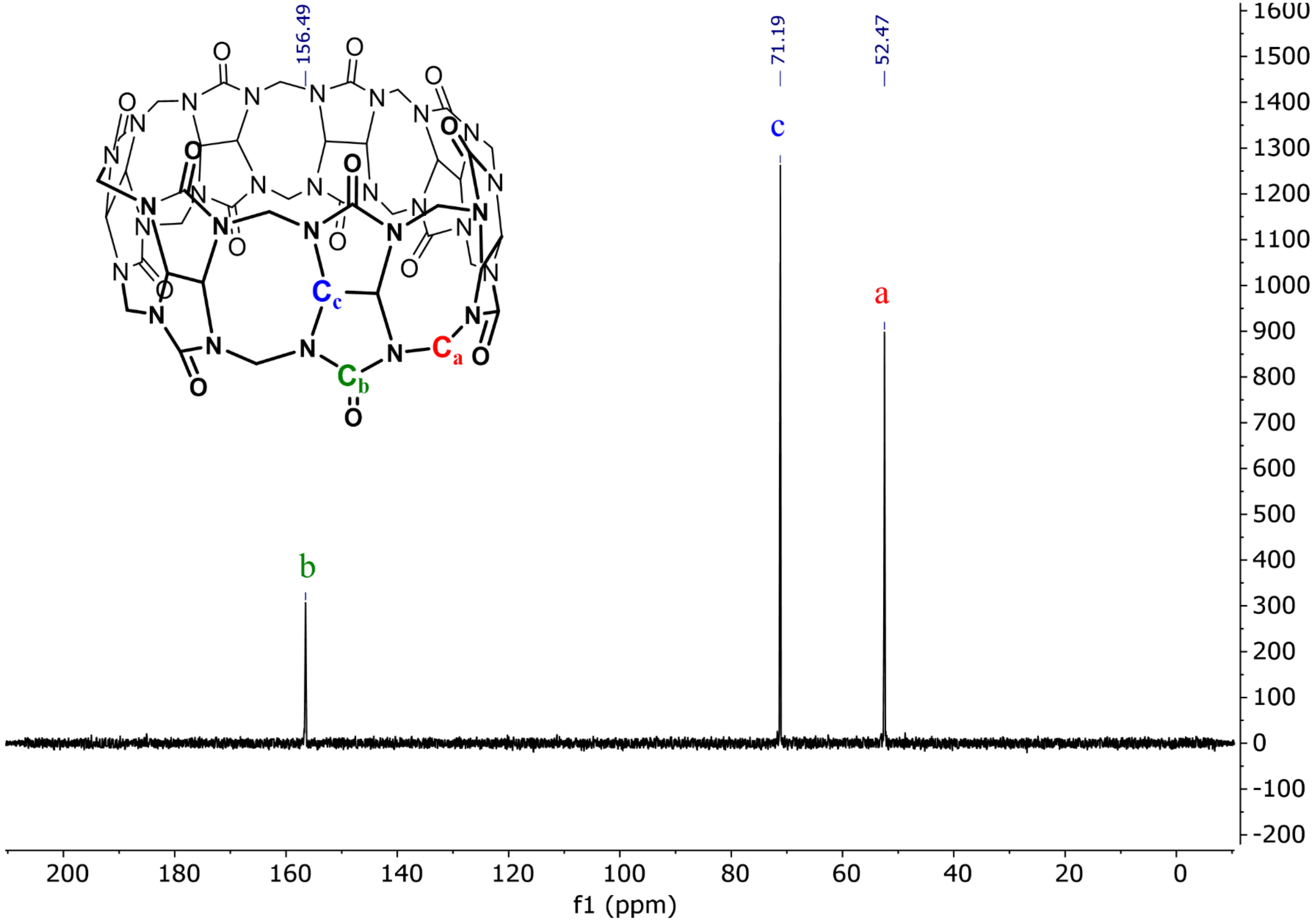

Figure S28. ^{13}C NMR spectrum of cucurbit[7]uril (CB[7]) (D_2O).

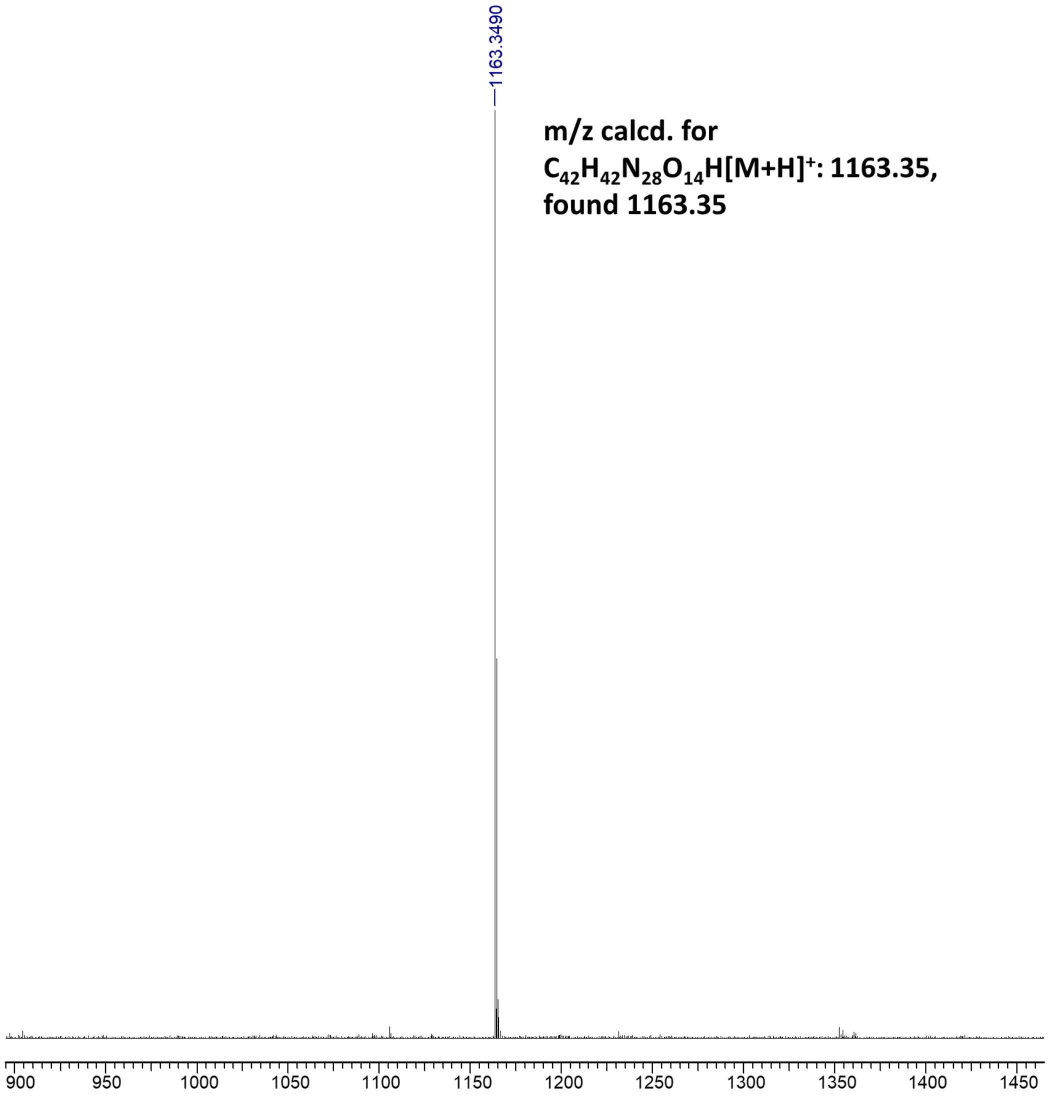

Figure S29. MALDI-TOF-MS spectrum of cucurbit[7]uril (CB[7]).

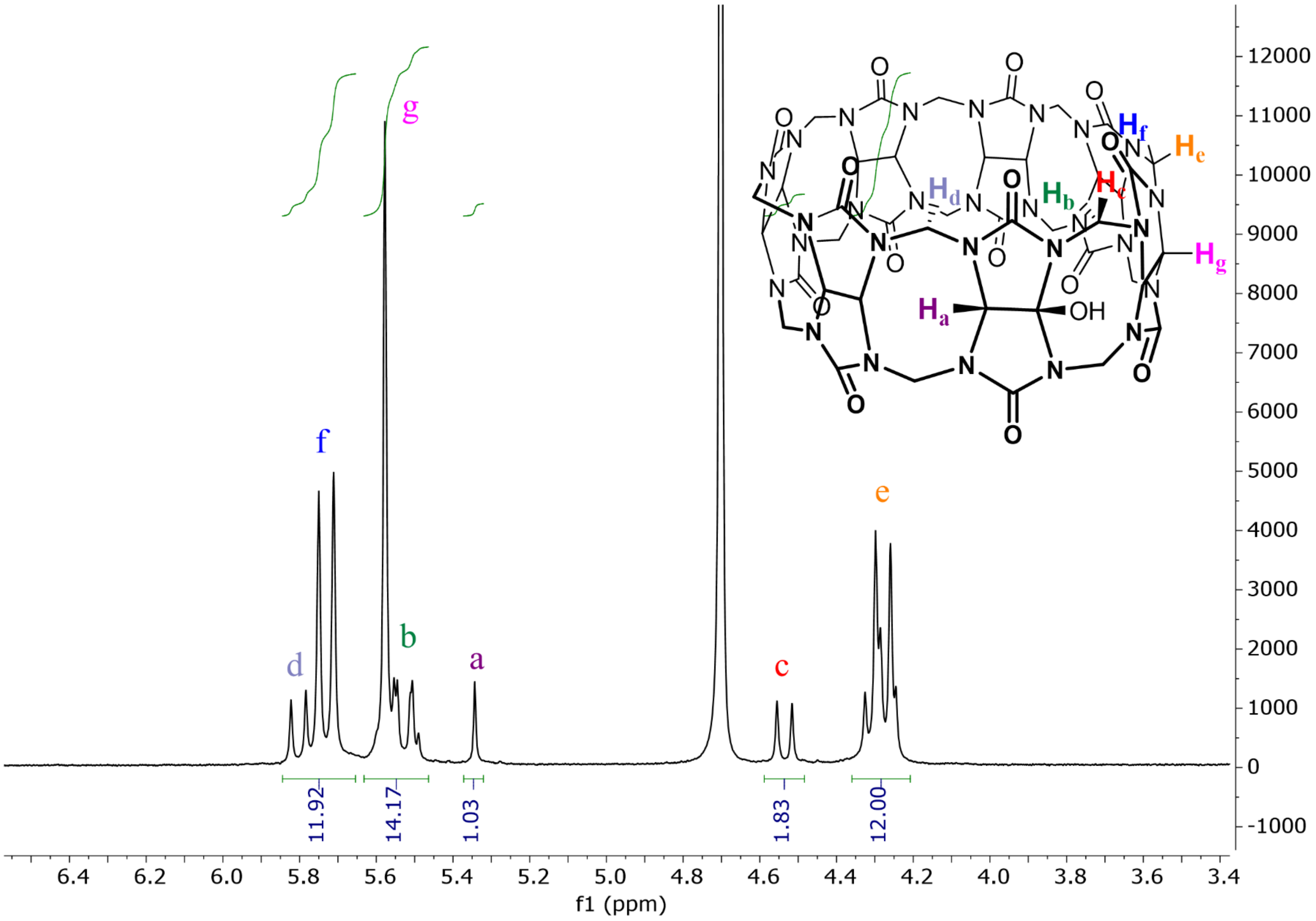

Figure S30. ^{1}H NMR spectrum of monohydroxy-cucurbit[7]uril (CB[7](OH)$_1$) (D_2O + NaCl).

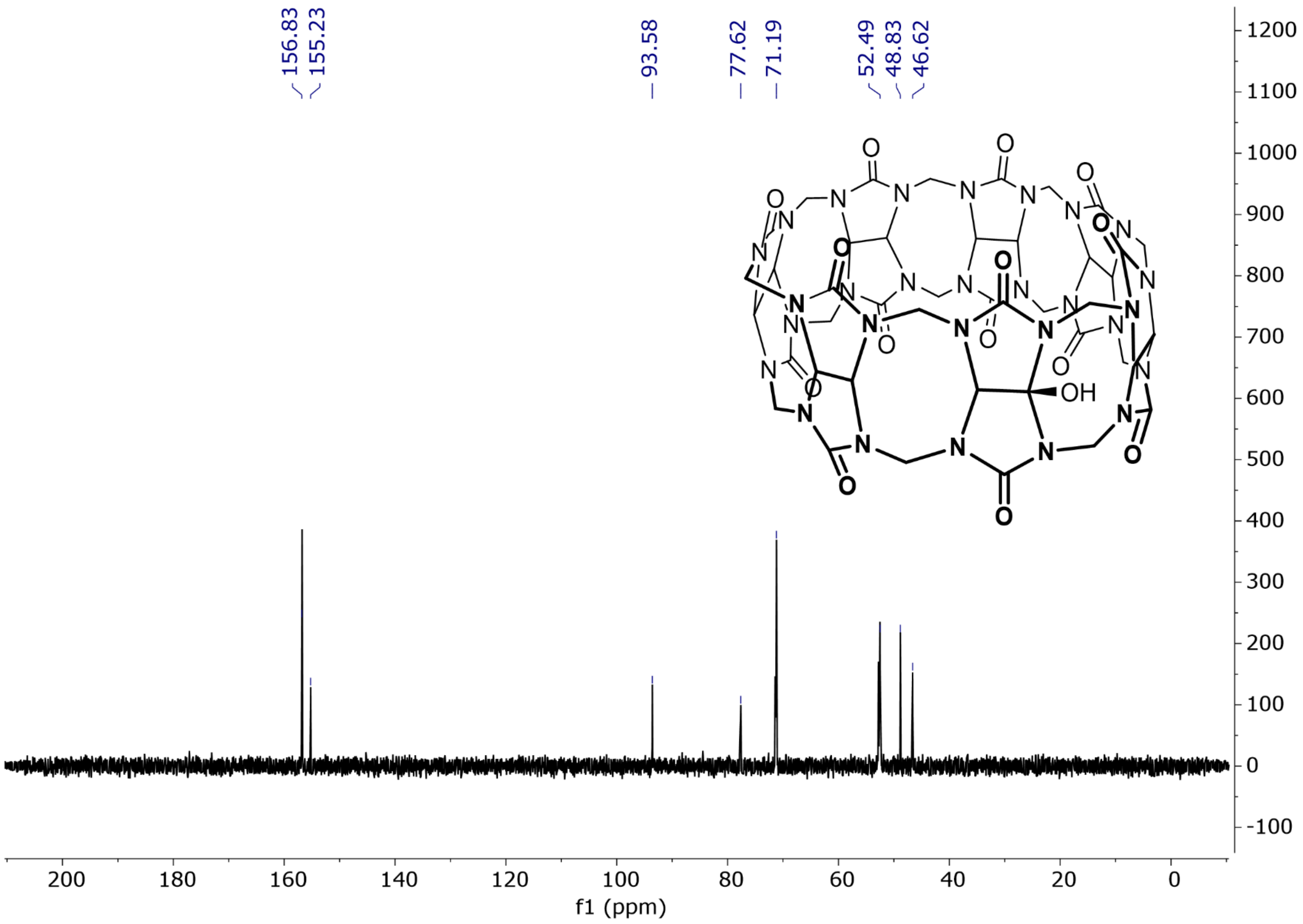

Figure S31. ^{13}C NMR spectrum of monohydroxy-cucurbit[7]uril (CB[7](OH)$_1$) (D_2O + NaCl).

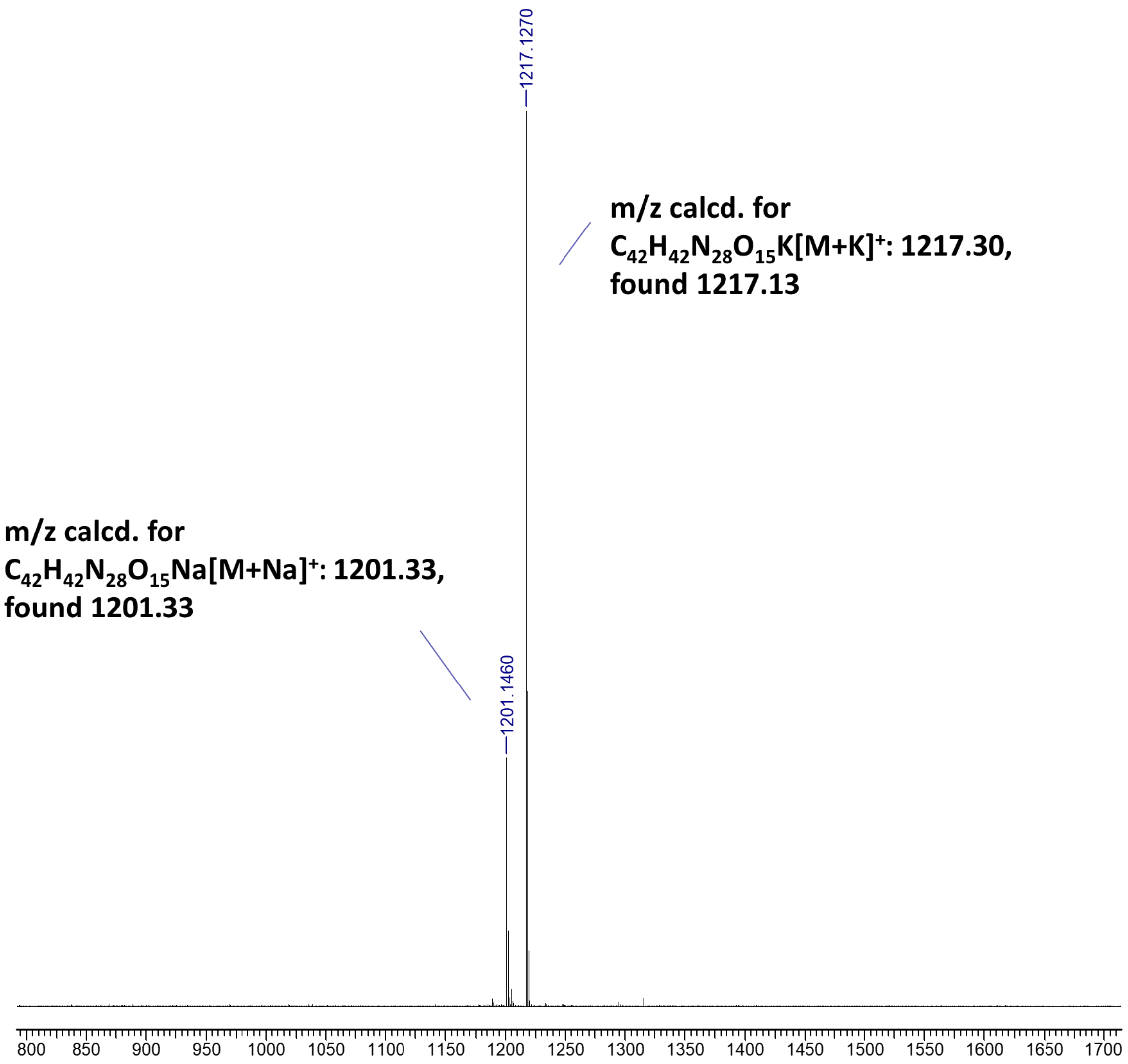

Figure S32. MALDI-TOF-MS spectrum of monohydroxy-cucurbit[7]uril (CB[7](OH)$_1$).

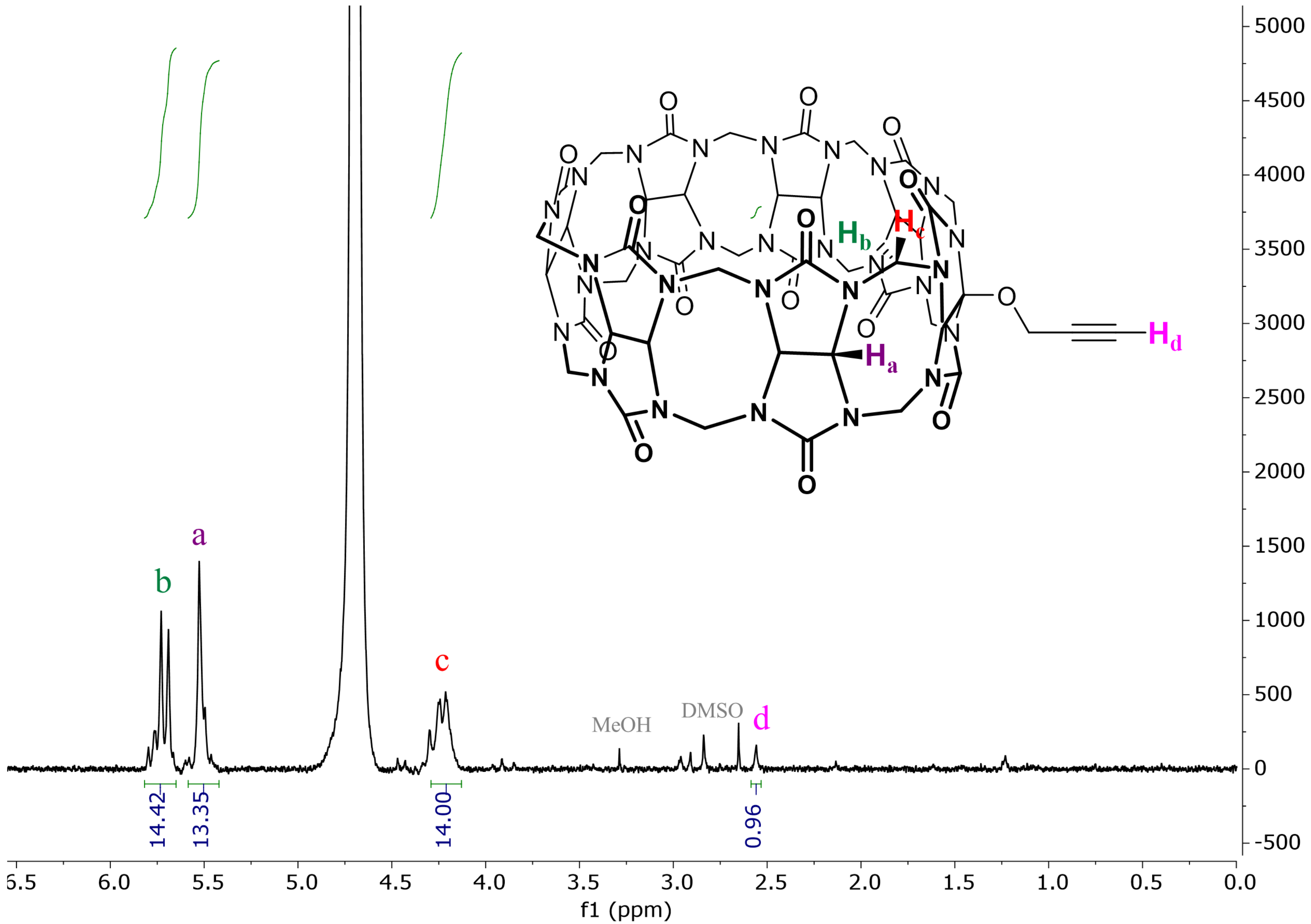

Figure S33. ^{1}H NMR spectrum of monopropargyl-cucurbit[7]uril (CB[7](OPr)$_1$) (D_2O + NaCl).

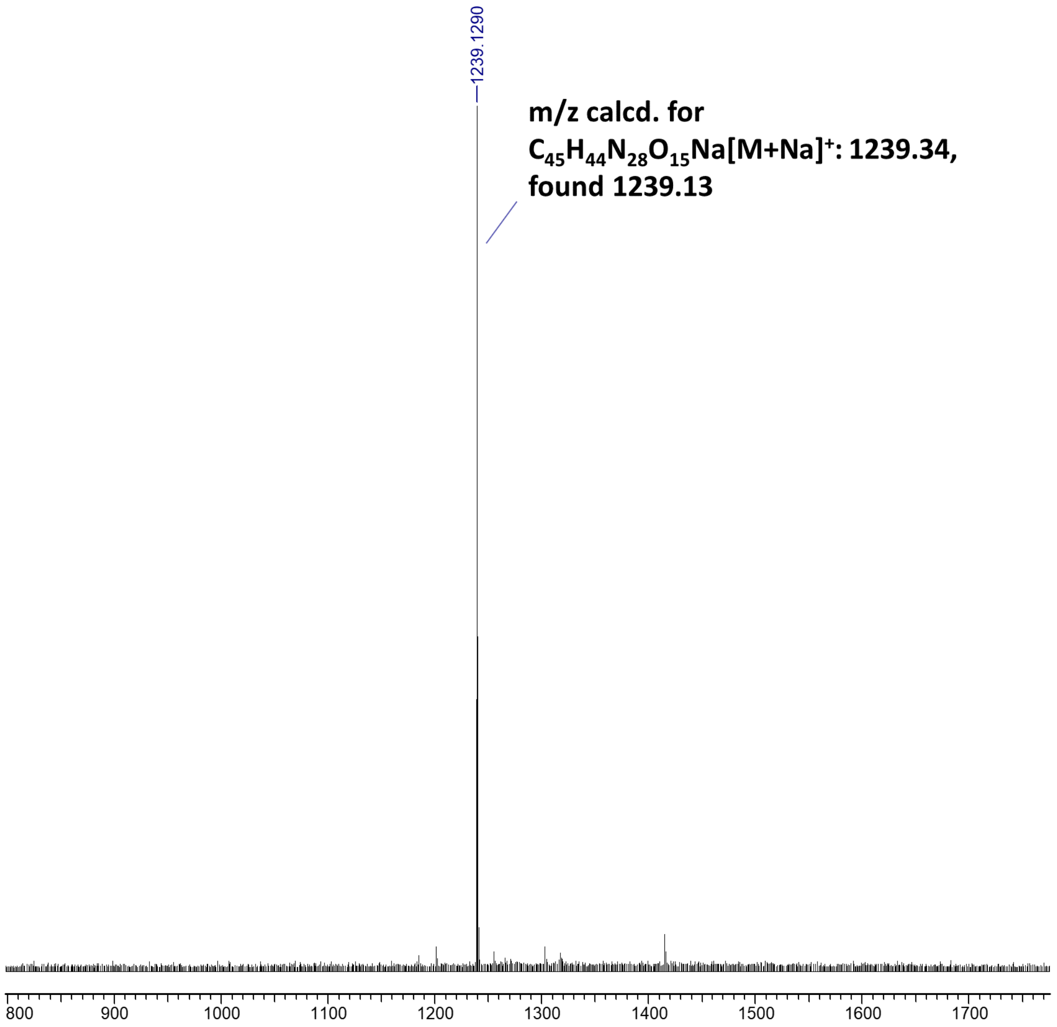

Figure S34. MALDI-TOF-MS spectrum of monopropargyl-cucurbit[7]uril (CB[7](OPr)$_1$).

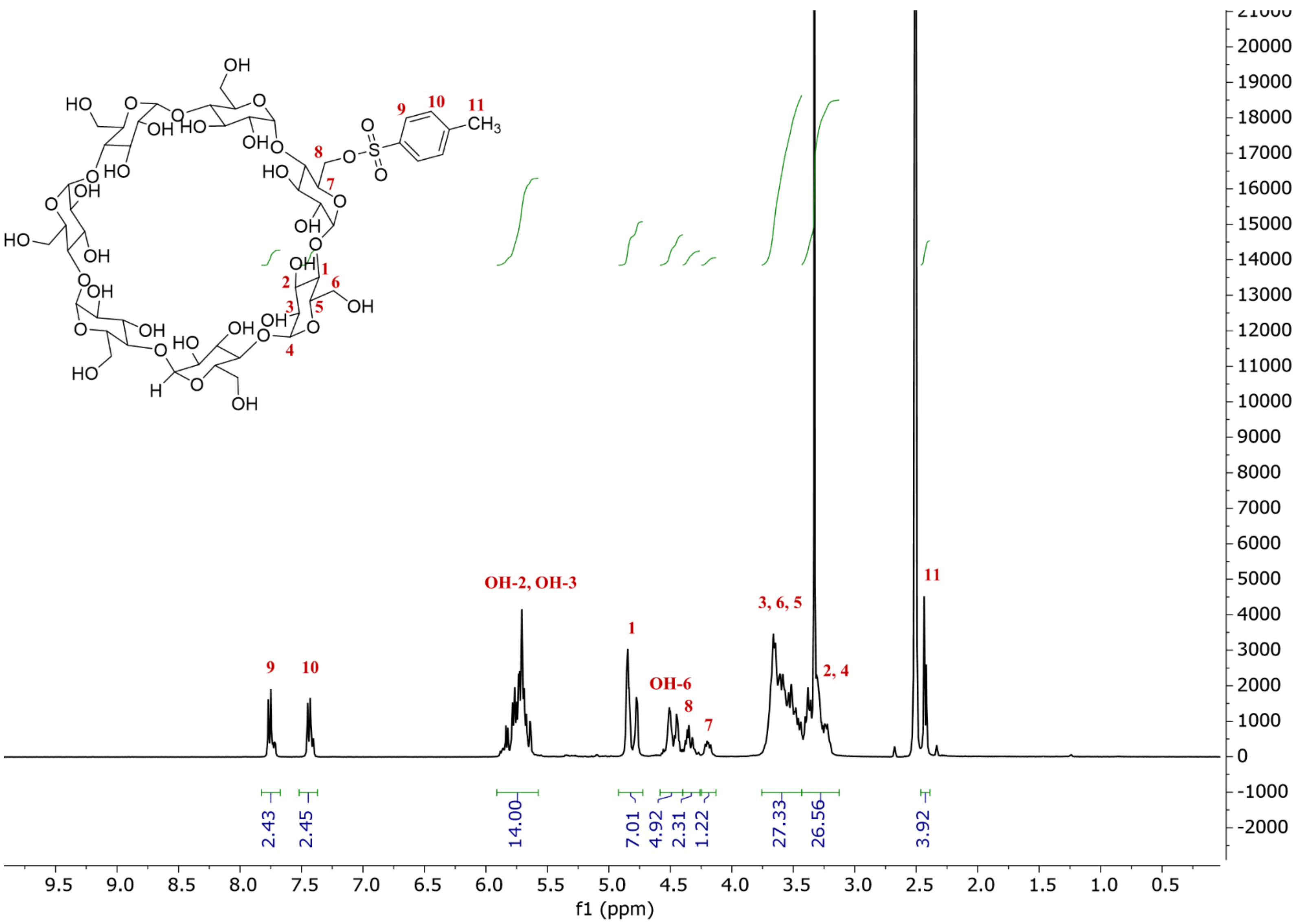

Figure S35. ^{1}H NMR spectrum of β-CD(OTs)$_1$ (DMSO-d6).

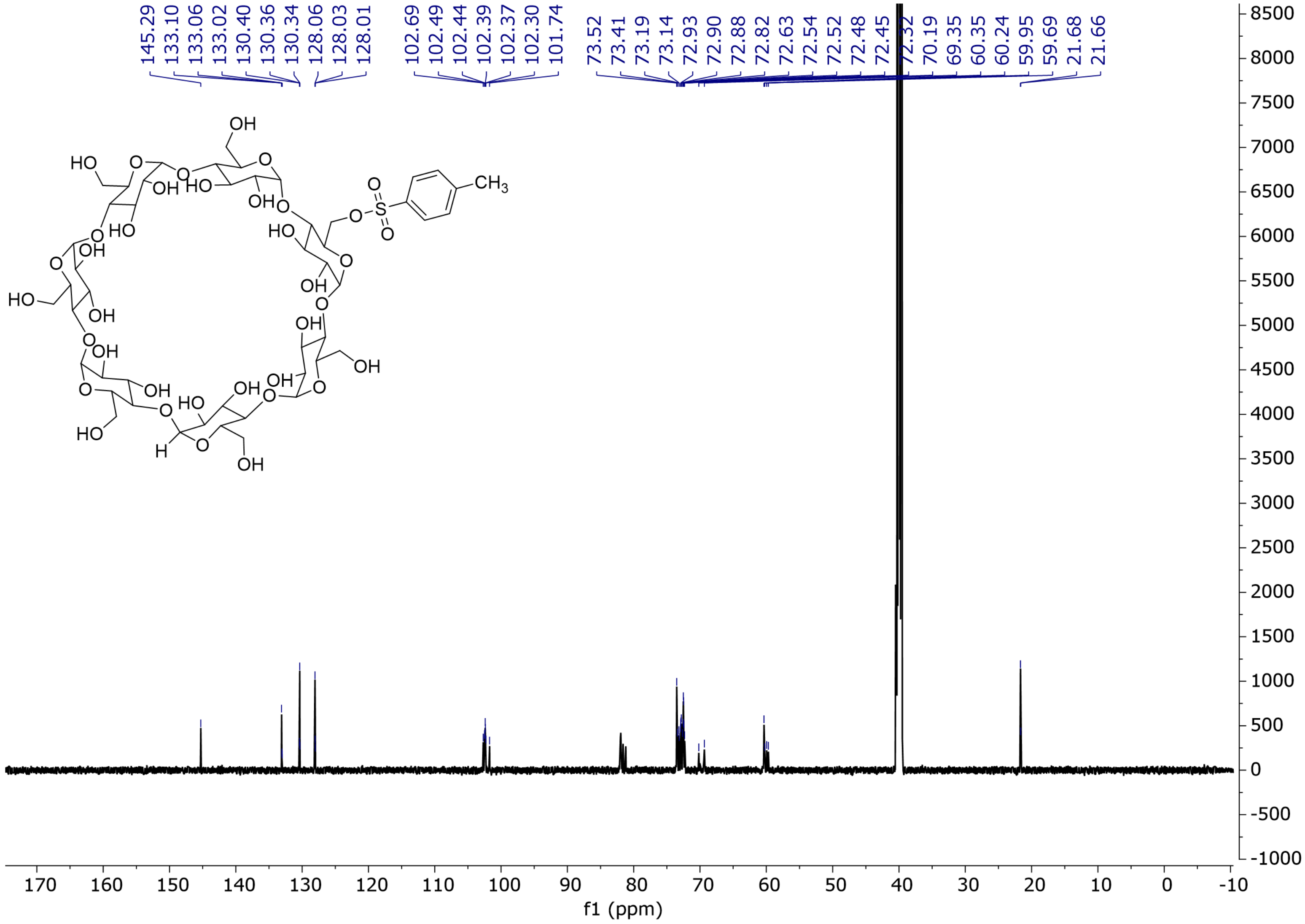

Figure S36. ^{13}C NMR spectrum of β-CD(OTs)$_1$ (DMSO-*d6*).

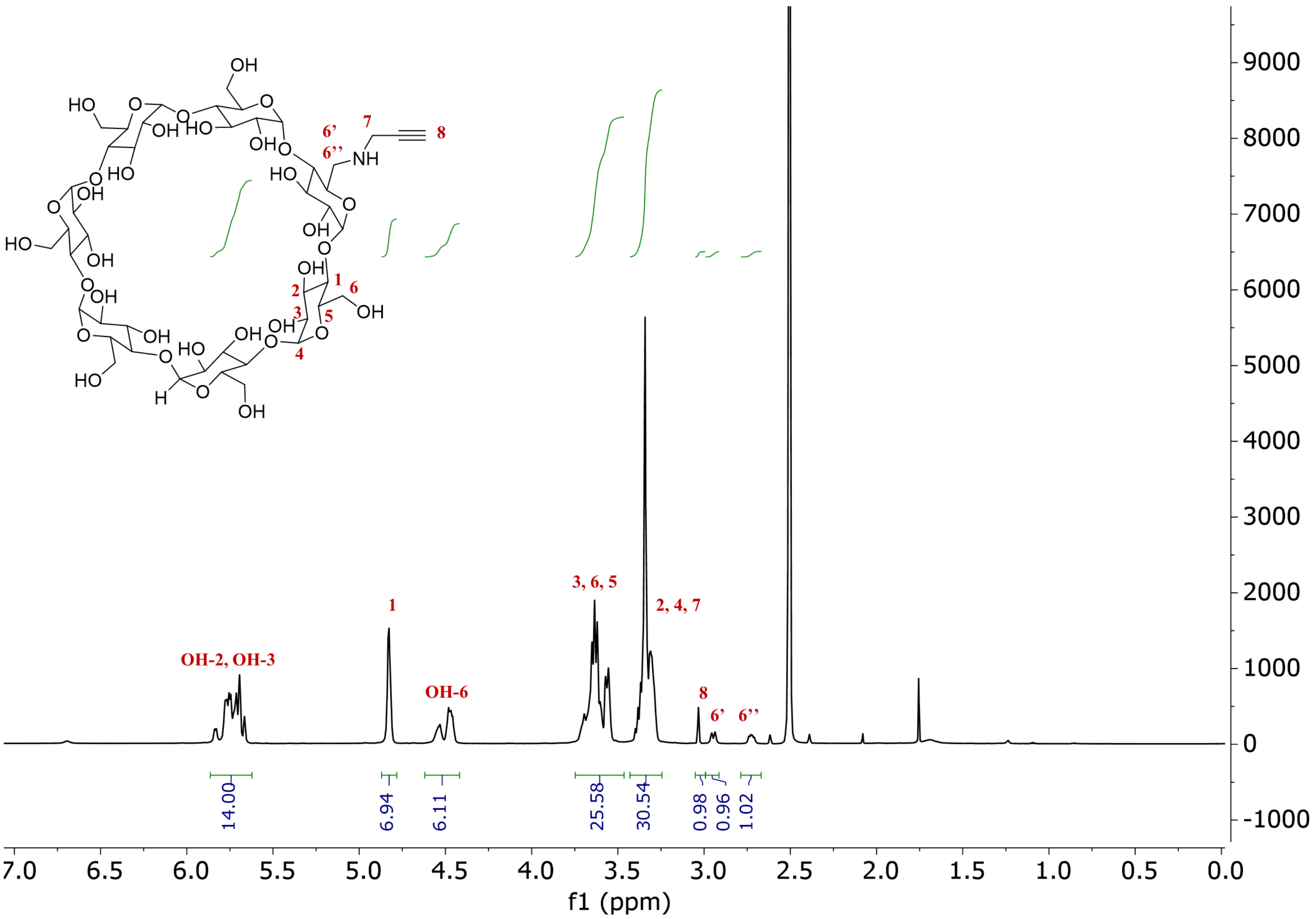

Figure S37. ^{1}H NMR spectrum of β-CD(NHPr)$_1$ (DMSO-*d6*).

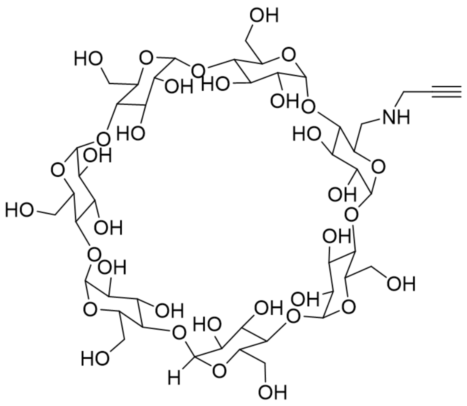

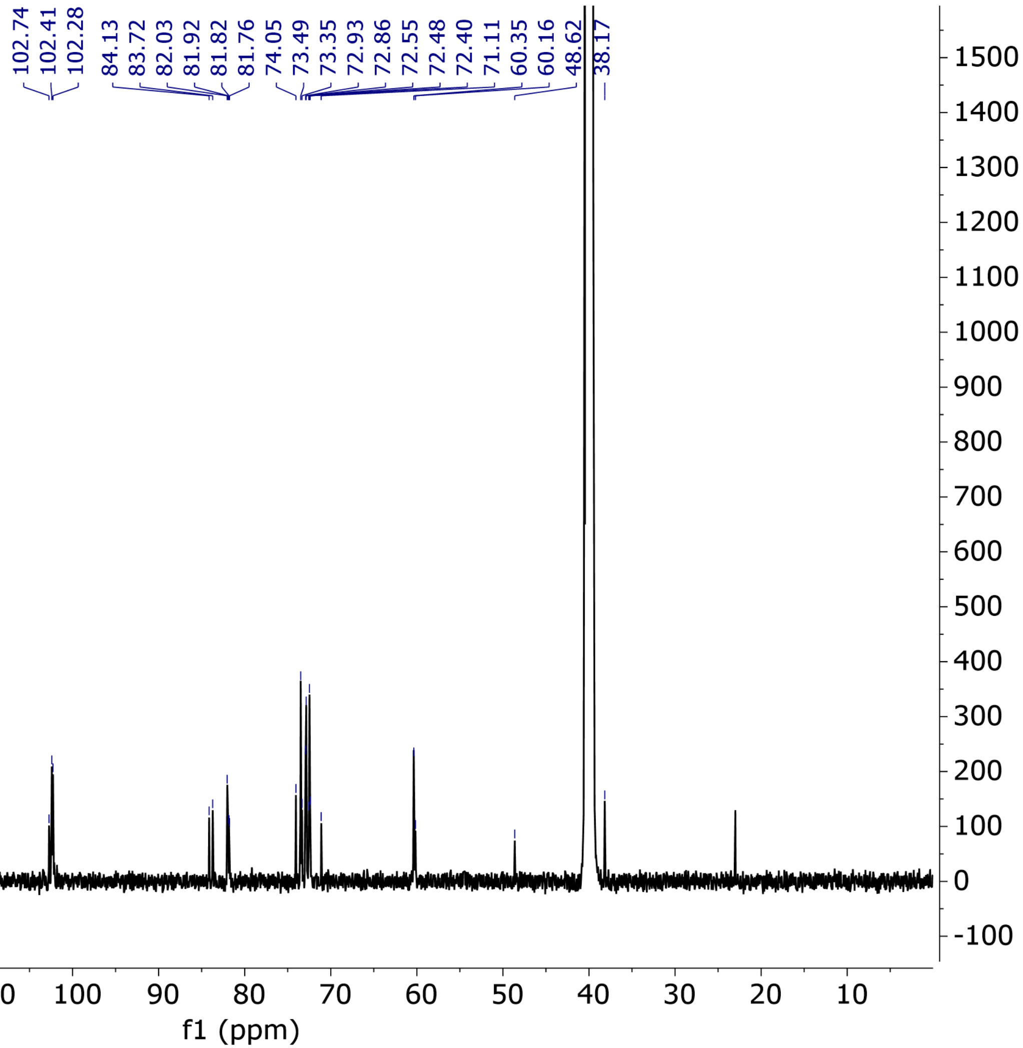

Figure S38. ^{13}C NMR spectrum of β-CD(NHPr)$_1$ (DMSO-*d6*).

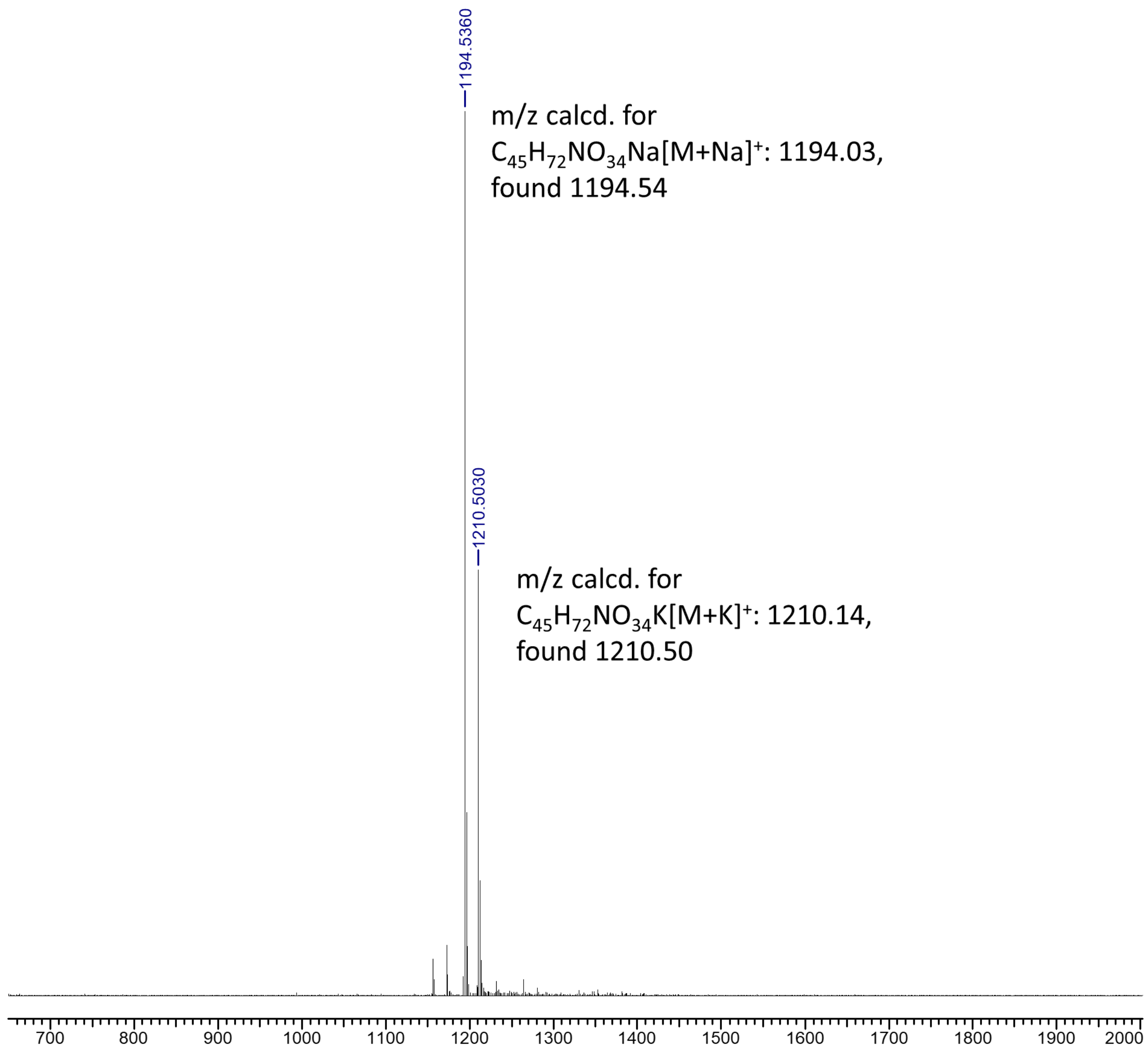

Figure S39. MALDI-TOF-MS spectrum of β-CD(NHPr)$_1$.

4. REFERENCES

(1) Zhang, Z.; Moxey, M.; Alswieleh, A.; Morse, A. J.; Lewis, A. L.; Geoghegan, M.; Leggett, G. J. Effect of Salt on Phosphorylcholine-Based Zwitterionic Polymer Brushes. *Langmuir* **2016**, *32* (20), 5048–5057. https://doi.org/10.1021/acs.langmuir.6b00763.

(2) Jin, Z.; Brash, J. L.; Zhu, S. ATRP Grafting of Oligo(Ethylene Glycol) Methacrylates from Gold Surface - Effect of Monomer Size on Grafted Chain and EO Unit Densities. *Can. J. Chem.* **2010**, *88* (5), 411–417. https://doi.org/10.1139/V10-010.

(3) Gleason, K. K.; Karaman, M.; Kooi, S. E. Vapor Deposition of Hybrid Organic-Inorganic Dielectric Bragg Mirrors Having Rapid and Reversibly Tunable Optical Reflectance. *Chem. Mater.* **2008**, *20* (6), 2262–2267. https://doi.org/10.1021/cm703107d.

(4) Wu, B.; Wang, X.; Yang, J.; Hua, Z.; Tian, K.; Kou, R.; Zhang, J.; Ye, S.; Luo, Y.; Craig, V. S. J.; Zhang, G.; Liu, G. Reorganization of Hydrogen Bond Network Makes Strong Polyelectrolyte Brushes PH-Responsive. *Sci. Adv.* **2016**, *2* (8). https://doi.org/10.1126/sciadv.1600579.

(5) Grim, J. C.; Brown, T. E.; Aguado, B. A.; Chapnick, D. A.; Viert, A. L.; Liu, X.; Anseth, K. S. A Reversible and Repeatable Thiol-Ene Bioconjugation for Dynamic Patterning of Signaling Proteins in Hydrogels. *ACS Cent. Sci.* **2018**, *4* (7), 909–916. https://doi.org/10.1021/acscentsci.8b00325.

(6) Guo, J. W.; Guan, N. Y.; Liu, S.; Yang, C. F.; Zhu, L. J. Synthesis and Characterizaion of Adamantane-Containing Quaternary Ammonium Salts. *Adv. Mater. Res.* **2011**, *233–235*, 238–241. https://doi.org/10.4028/www.scientific.net/AMR.233-235.238.

(7) Shieh, P.; Hangauer, M. J.; Bertozzi, C. R. Fluorogenic Azidofluoresceins for Biological Imaging. *J. Am. Chem. Soc.* **2012**, *134* (42), 17428–17431. https://doi.org/10.1021/ja308203h.

(8) Gomes, A. C.; Magalhães, C. I. R.; Oliveira, T. S. M.; Lopes, A. D.; Gonçalves, I. S.; Pillinger, M. Solid-State Study of the Structure and Host-Guest Chemistry of Cucurbituril-Ferrocene Inclusion Complexes. *Dalt. Trans.* **2016**, *45* (42), 17042–17052. https://doi.org/10.1039/c6dt02811j.

(9) Ahn, Y.; Jang, Y.; Selvapalam, N.; Yun, G.; Kim, K. Supramolecular Velcro for Reversible Underwater Adhesion. *Angew. Chemie Int. Ed.* **2013**, *52* (11), 3140–3144. https://doi.org/10.1002/anie.201209382.

(10) Zhang, S.; Domínguez, Z.; Assaf, K. I.; Nilam, M.; Thiele, T.; Pischel, U.; Schedler, U.; Nau, W. M.; Hennig, A. Precise Supramolecular Control of Surface Coverage Densities on Polymer Micro- and Nanoparticles. *Chem. Sci.* **2018**, *9* (45), 8575–8581. https://doi.org/10.1039/C8SC03150A.

(11) Guo, Z.; Jin, Y.; Liang, T.; Liu, Y.; Xu, Q.; Liang, X.; Lei, A. Synthesis, Chromatographic Evaluation and Hydrophilic Interaction/Reversed-Phase Mixed-Mode Behavior of a “Click β-Cyclodextrin” Stationary Phase. *J. Chromatogr. A* **2009**, *1216* (2), 257–263. https://doi.org/10.1016/j.chroma.2008.11.071.

(12) Su, Q.; Vogt, S.; Nöll, G. Langmuir Analysis of the Binding Affinity and Kinetics for Surface Tethered Duplex DNA and a Ligand–Apoprotein Complex. *Langmuir* **2018**, *34* (49), 14738–14748. https://doi.org/10.1021/acs.langmuir.7b04347.
